# The *Physalis floridana* genome provides insights into the biochemical and morphological evolution of *Physalis* fruits

**DOI:** 10.1038/s41438-021-00705-w

**Published:** 2021-11-18

**Authors:** Jiangjie Lu, Meifang Luo, Li Wang, Kunpeng Li, Yongyi Yu, Weifei Yang, Pichang Gong, Huihui Gao, Qiaoru Li, Jing Zhao, Lanfeng Wu, Mingshu Zhang, Xueyang Liu, Xuemei Zhang, Xian Zhang, Jieyu Kang, Tongyuan Yu, Zhimin Li, Yuannian Jiao, Huizhong Wang, Chaoying He

**Affiliations:** 1 State Key Laboratory of Systematic and Evolutionary Botany, Institute of Botany, Chinese Academy of Sciences, Nanxincun 20, 100093 Xiangshan, Beijing China; 2 Zhejiang Provincial Key Laboratory for Genetic Improvement and Quality Control of Medicinal Plants, College of Life and Environmental Science, Hangzhou Normal University, 310036 Hangzhou, China; 3 University of Chinese Academy of Sciences, Yuquan Road 19, 100049 Beijing, China; 4 Annoroad Gene Technology (Beijing) Co, Ltd, 100176 Beijing, China; 5 The Innovative Academy of Seed Design, Chinese Academy of Sciences, Beijing, China

**Keywords:** Plant evolution, Comparative genomics

## Abstract

The fruits of *Physalis* (Solanaceae) have a unique structure, a lantern-like fruiting calyx known as inflated calyx syndrome (ICS) or the Chinese lantern, and are rich in steroid-related compounds. However, the genetic variations underlying the origin of these characteristic traits and diversity in *Physalis* remain largely unknown. Here, we present a high-quality chromosome-level reference genome assembly of *Physalis floridana* (~1.40 Gb in size) with a contig N50 of ~4.87 Mb. Through evolutionary genomics and experimental approaches, we found that the loss of the *SEP*-like MADS-box gene *MBP21* subclade is likely a key mutation that, together with the previously revealed mutation affecting floral *MPF2* expression, might have contributed to the origination of ICS in Physaleae, suggesting that the origination of a morphological novelty may have resulted from an evolutionary scenario in which one mutation compensated for another deleterious mutation. Moreover, the significant expansion of squalene epoxidase genes is potentially associated with the natural variation of steroid-related compounds in *Physalis* fruits. The results reveal the importance of gene gains (duplication) and/or subsequent losses as genetic bases of the evolution of distinct fruit traits, and the data serve as a valuable resource for the evolutionary genetics and breeding of solanaceous crops.

## Introduction

The family Solanaceae is an important source of nutritional and culinary diversity. Along with several well-characterized model crops, such as potato (*Solanum tuberosum*), tomato (*Solanum lycopersicum*), and peppers (*Capsicum* spp.), the nightshade family contains many neglected orphan crops^1–3^. Members of the genus *Physalis* include well-known species such as Cape gooseberry (*Physalis peruviana*) and tomatillo or husk tomato (*Physalis philadelphica*). *Physalis* is one of the largest genera within Solanaceae, with 70–90 species^4^. Species in this genus have emerged as a new class of solanaceous horticultural crops as well as new model plants for studies in ecology, evolution, and development^3,5^, as the genus is characterized by novel morphological traits and biochemical diversity in fruits. Understanding how such complex traits originated during evolutionary history is a fundamental question, since fruit evolution has long been considered a key contributor to the success of angiosperms^6^. Moreover, the origin of these evolutionary novelties is a fascinating subject in itself, and the processes giving rise to them are largely unresolved evolutionary mysteries.

**Fig. 1:**
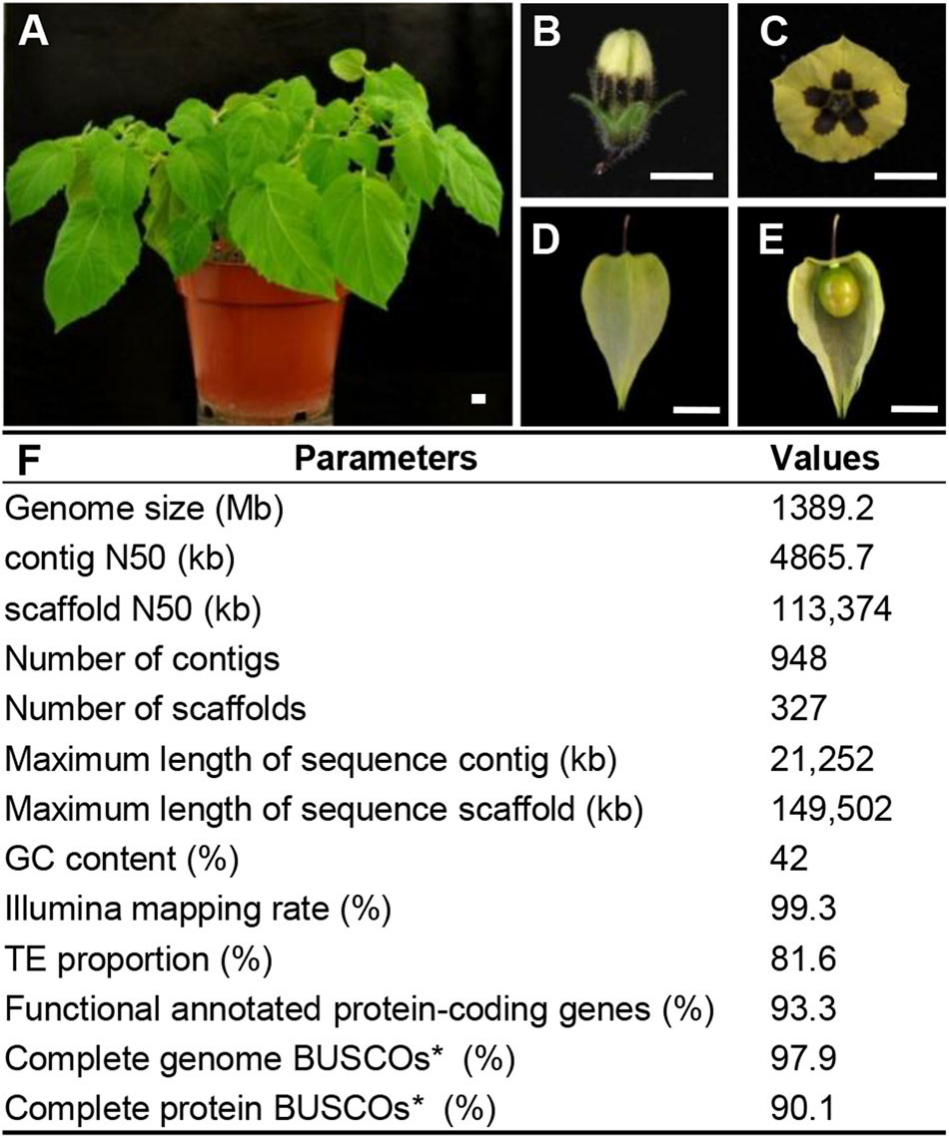
Morphology and genome assembly of *P. floridana*. **A** A *P. floridana* plant. **B** A flower bud. **C** A flower. **D** A mature fruit. **E** A berry. The Chinese lantern was partially removed to show the berry inside. Bar = 1.0 cm. **F** Characteristics of the *P. floridana* genome assembly. *indicates BUSCO using the embryophyta_odb10 database with 1375 core proteins

Most *Physalis* species have 12 chromosomes, as observed in most solanaceous species^7^. Members of the genus have a distinct fruit morphology with a papery husk as an accessory trait^8,9^; this characteristic structure is known as inflated calyx syndrome (ICS) or Chinese lantern^5,8^. Within Solanaceae, at least five genera (*Physalis*, *Withania*, *Przewalskia*, *Margaranthus*, and *Nicandra*) of Physaleae share this morphological novelty^10^. *Physalis floridana* is a representative species of *Physalis* characterized by solitary flowers with dark maculations and berries that are enveloped by an inflated fruiting calyx (Fig. 1A–E). The adaptive advantages of this novelty have been well documented at developmental, physiological, mechanical, and ecological levels, and selection apparently favors the fixation of this trait to improve plant fitness^11,12^. The lantern trait appears as an inflated fruiting calyx; fertilization/hormonal signals trigger its formation in *Physalis* and *Withania*^5,11,13^. The question of how this novelty arose has attracted the attention of botanists and evolutionary biologists. Previous studies revealed that the origin of the Chinese lantern is associated with the heterotopic expression of *Physalis* MADS-box gene 2 (*MPF2*) in floral organs^5^. Moreover, floral calyx identity and Chinese lantern size are determined by *Physalis* MADS-box gene 3 (*MPF3*), which interacts with *MPF2* both physically and genetically^14^. The Darwinian selection of *MPF2*-like genes involved directional selection for ICS^15,16^. ICS is considered a plesiomorphic trait based on the evaluation of *MPF2*-like expression in flowers^10^; however, the recently elucidated evolutionary history of Physalideae, which contains the vast majority of Solanaceae species with inflated calyces (146 of 222 in total), using four neutral genetic markers (*ITS*, *LEAFY*, *trnL-F*, and *waxy*) suggested strong directionality in the origin of ICS^17^. To better understand the origin and evolutionary and developmental mechanisms of the Chinese lantern within Solanaceae, further investigation at the genomic level is needed.

As a result of adaptive evolution, plants produce secondary metabolites as protective agents^18^ or to attract pollinators^19^. Some of the secondary metabolites in *Physalis* fruits and plants render them commercially valuable, and many such compounds have antibacterial, anti-inflammatory, and anticancer activities that are useful in medical applications. The whole herb of *Physalis* is used in traditional medicine to treat fever, diabetes, pharyngitis, boils, coughs, and mastitis^20–22^. Fruits, including the fruiting calyces of a few *Physalis* species, often have curative and culinary uses^1,23^, and berries are used as both medicine and food. Increasing attention has been paid to *Physalis* in phytochemical and pharmacological studies, as its chemical constituents include withanolides, sucrose esters, flavonoids, labdane diterpenes, ceramides, and chlorogenic acids^24^, which are crucial for plant development and growth^25^. To date, a total of 351 natural ergostane steroid compounds with novel and unique structures, including withanolides, have been identified in the genus *Physalis*^26^. The two general five-carbon (C_5_) isoprene units of phytosterols and subsequent steroid biosynthesis are isopentenyl diphosphate (IPP) and dimethylallyl diphosphate (DMAPP) that are produced via the cytoplasmic mevalonate (MVA) pathway and the chloroplastic methylerythritol phosphate (MEP) pathway^27^. The biosynthetic pathways of these phytosterols and the structure-activity correlations of all isolated biochemical compounds have been proposed, all of which seem to be conserved in *Solanum* and *Physalis*^28,29^. However, the diversity, biosynthesis, and metabolic pathways of steroids and steroid derivatives in *Physalis* fruits are largely unexplored.

In the present study, we generated a chromosome-level genome assembly of *P. floridana* through the combined application of Pacific Biosciences (PacBio) sequencing and chromosome conformation capture (Hi-C) technologies. Using this resource combined with evolutionary and functional analyses, we obtained new insights into the evolutionary genetic basis of the iconic fruit morphological novelty (the Chinese lantern) and characteristic chemical composition (physalins) of *Physalis* fruits, pinpointing the roles of gene gains (duplication) and/or subsequent losses in the origin and evolution of the morphological novelty and biochemical variation. This first high-quality reference genome of *P. floridana* will serve as a valuable resource for breeding and improving the horticultural, nutritional, and medicinal value of *Physalis* crops.

## Results

### High-quality genome assembly of *P. floridana*

We selected the diploid and self-compatible *P. floridana* P106 accession for the first trial aimed at decoding the genome of a *Physalis* species because this accession shares the typical flower and fruit morphology of *Physalis* species, characterized by a solitary flower and a berry covered with a lantern-like fruiting calyx (Fig. 1A–E; see Supplementary Table 1 for details). Chromosomal karyotype analysis revealed that the P106 genome contained 12 pairs of chromosomes (2*n* = 24) (Supplementary Fig. 1). First, we generated 153 Gb of whole-genome data from shotgun sequences of P106 via the Illumina sequencing of genomic libraries with an insert size of 500 bp (Supplementary Fig. 2), from which 149 Gb of clean data was yielded after removing low-quality reads. The main Poisson-shaped distribution peak (depth = 91), with a minor peak and long tail (depth >163), represented the unique 21-mers found in the *P. floridana* genome (Supplementary Fig. 3), suggesting that the genome is nearly homozygous, with substantial repeat sequences. The genome size was estimated to be ~1.40 Gb by K-mer analysis and flow cytometry (Supplementary Figs. 3 and 4; Supplementary Table 2). Then, two PacBio libraries were constructed and sequenced on five cells using the PacBio Sequel platform, yielding 125 Gb of subreads corresponding to ~90-fold coverage of the genome (Supplementary Fig. 5 and Supplementary Fig. 6A, B). The PacBio reads were assembled into 922 contigs with a contig N50 of 4.87 Mb (Supplementary Table 3). Furthermore, ~215 million uniquely mapped paired-end reads (25% of 858 million cleaned Hi-C reads) were generated, among which ~196 million (91.35%) were valid paired-end reads and were used to generate chromosome contact information (Supplementary Table 4). Finally, a total of 1.37 Gb of data were clustered into 12 pseudochromosomes, which contained 98.82% of the total assembly length (Supplementary Figs. 7 and 8).

We then remapped the Illumina reads to the assemblies (mapping rate ~ 99.93%; mean depth 86.75x) (Supplementary Table 5). Single nucleotide polymorphisms were called to further estimate the level of heterozygosity, which was ~0.19% for the P106 genome (Supplementary Table 6). A guanine-cytosine (GC) depth analysis revealed that the *Physalis* genome had a mean GC content of 42% (Supplementary Fig. 6C, D).

We also generated the full-length transcriptome of *P. floridana* based on PacBio long reads and Illumina transcriptomes from various biological tissues. By mapping the transcriptome reads to the genome assembly, we found that the mapping rates were ~89.44%–99.61% (Supplementary Table 7), indicating the high completeness of the genome assembly. We further evaluated the completeness of the scaffold assembly using the BUSCO (v.3) plant datasets and identified 97.9% of the 1,375 single-copy core sets of orthologous genes in the *Physalis* genome assembly (Supplementary Table 8). Taken together, the genome assembly assessments (Fig. 1F) suggest that we obtained a high-quality reference genome for *Physalis*.

### Genome annotation and gene prediction

**Fig. 2:**
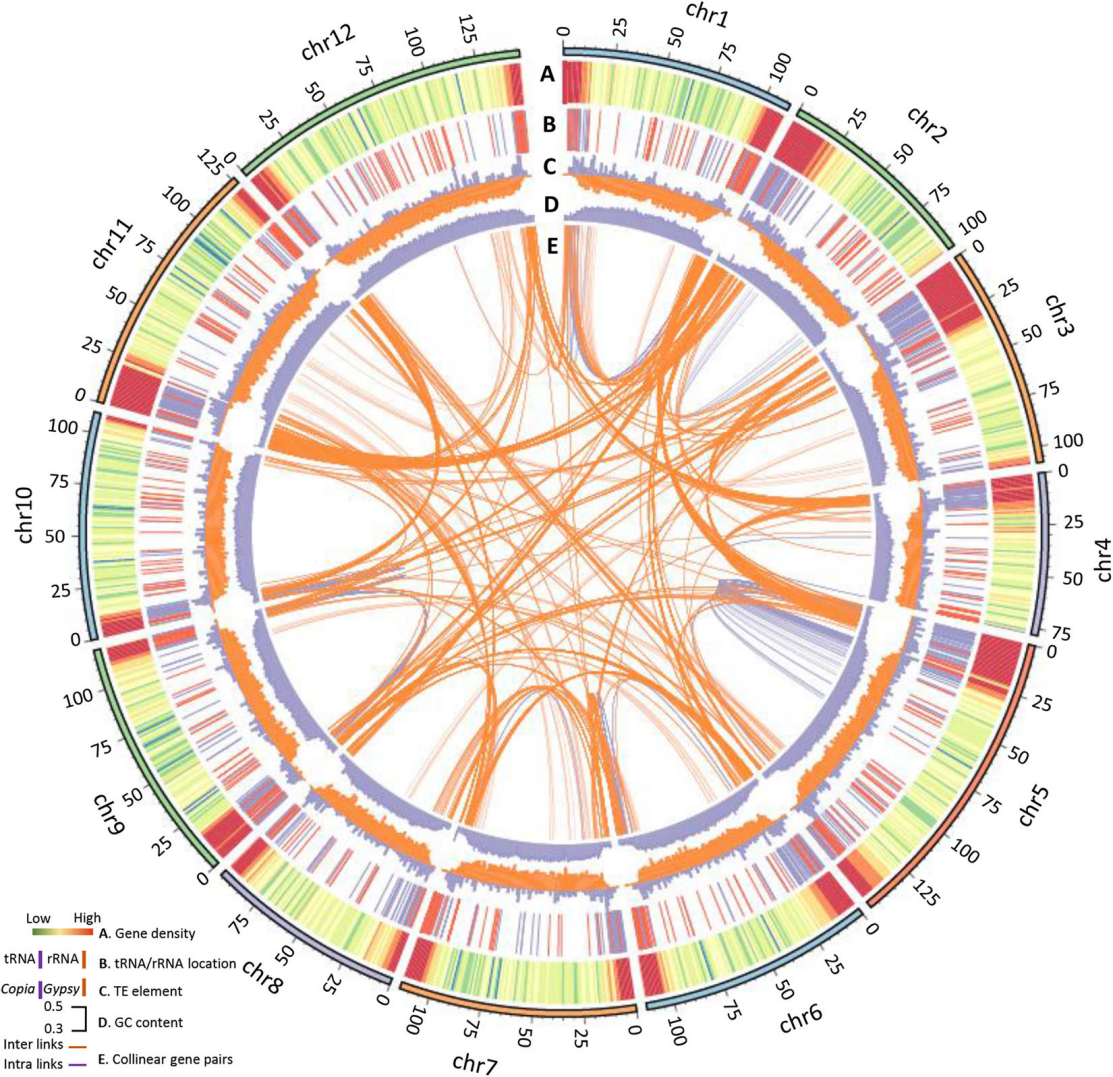
High-quality genome of *P. floridana* integrated with the indicated genetic data. **A** Gene density plotted in 1000-kb sliding windows. **B** tRNA (purple) and rRNA (orange) locations. **C** TE/*Copia* (purple) and TE/*Gypsy* (orange) content per Mb. **D** GC content per 300-kb block. **E** Genome segmental duplication and internal rearrangement events. Orange, intercollinear gene pairs; purple, intracollinear gene pairs

The prediction and density of genes (Fig. 2A), including protein-coding genes and the tRNA and rRNA genes (Fig. 2B); transposable elements (TEs), i.e., *Copia* and *Gypsy* (Fig. 2C); and other information, such as the GC distribution (Fig. 2D) and genome rearrangement events of collinear blocks (Fig. 2E), were evaluated and integrated into the assembled *Physalis* genome. For this purpose, ab initio and homology-based methods were combined to annotate protein-coding sequences aided by the transcriptome sequences. In total, 32,075 complete protein-coding genes were predicted in the current genome version, with an average gene length, coding sequence length, and exon number of 4,023 bp, 1,134 bp, and 4.63, respectively (Fig. 2A and Supplementary Figs. 9 and 10; Supplementary Table 9). Among these annotated genes, 90.1% of the 1,375 single-copy core sets of orthologous amino acid sequences could be identified (Fig. 1F), and 93.34% (29,938) could be annotated based on publicly available databases (Supplementary Fig. 11). We further predicted 3,655 ribosomal RNA (rRNA), 997 transfer RNA (tRNA), 375 microRNA (miRNA), and 3,047 small nuclear RNA (snRNA) genes in the *P. floridana* genome (Fig. 2B and Supplementary Table 10).

The total length of the identified repetitive sequences in the *P. floridana* genome was 1142.5 Mb, occupying 82.24% of the assembled genome sequences (Supplementary Table 11). This proportion was similar to that observed in *S. pennellii* (82%)^30^ and higher than that in the *C. annuum* genome (76.4%)^31^, the *S. lycopersicum* genome (61.3%)^32^, and the *S. tuberosum* genome (61.6%)^33^, while TEs accounted for 81.6% of the assembled sequence (Fig. 1F). Among the identified TEs, LTR retrotransposons (LTR-RT), which usually play a substantial role in genome size variation^34,35^, represented 65.68% of the *P. floridana* genome (Fig. 2 and Supplementary Fig. 12A; Supplementary Table 12). This proportion of LTR-RTs in the *P. floridana* genome was lower than that in *C. annuum* (70%) and higher than that in *S. tuberosum* (47%), *S. lycopersicum* (50%), and *S. pennellii* (45%)^30–33^. We therefore assessed the recent activity of LTR-RTs in five Solanaceae species using a sequence alignment of full-length LTR-RTs and estimated their insertion times. The timing of the main LTR-RT burst was earliest in *C. annuum* (~2.0 MYA), while *S. lycopersicum* showed an intermediate insertion time (~0.8 MYA), and *S. pennellii*, *S. tuberosum*, and *P. floridana* exhibited many more recent LTR-RT bursts (~0.4 MYA) (Supplementary Fig. 12B). Similar to the *C. annuum* genome, the substantial proliferation of the *Gypsy* family (10-fold more than the *Copia* family) identified in the *Physalis* genome (Fig. 2C) might be the main cause of genome expansion, as previously proposed^31^. Unlike LTR retrotransposons, DNA transposons comprised only 0.71% of the *Physalis* genome, whereas 15.41% of the genome corresponded to uncharacterized repeats (Supplementary Table 12). Moreover, significant genome segmental duplication and rearrangements occurred during the evolution of the *P. floridana* genome (Fig. 2E). Thus, the different genome dynamics in these Solanaceae species might be largely due to repetitive sequences, particularly *Gypsy* TE variations, and to genome segmental duplication and internal rearrangements.

### Genome evolution analyses

Phylogenomic analysis using 7,553 single-copy gene families revealed the topology of *P. floridana* and 12 other representative species (Supplementary Fig. 13). We found that within this subclade, *P. floridana* and *C. annuum* diverged from each other ~23.8 (18.0–29.9) million years ago (MYA), while *S. lycopersicum, S. pennellii*, and *S. tuberosum* belonged to another subclade that diverged from the *P. floridana–C. annuum* subclade ~29.9 (23.9–36.6) MYA (Supplementary Fig. 14). These results were in line with recent molecular divergence estimates in which the fossils used were considerably older than the ~30 MYA crown of the entire Solanaceae family^36^ but were inconsistent with the report that *Physalis infinemundi* sp. nov. represents a derived lineage of Solanaceae from Gondwanan South America from 52.2 MYA, thereby considerably pushing back the timing of the evolutionary origin of this plant family^37^.

**Fig. 3:**
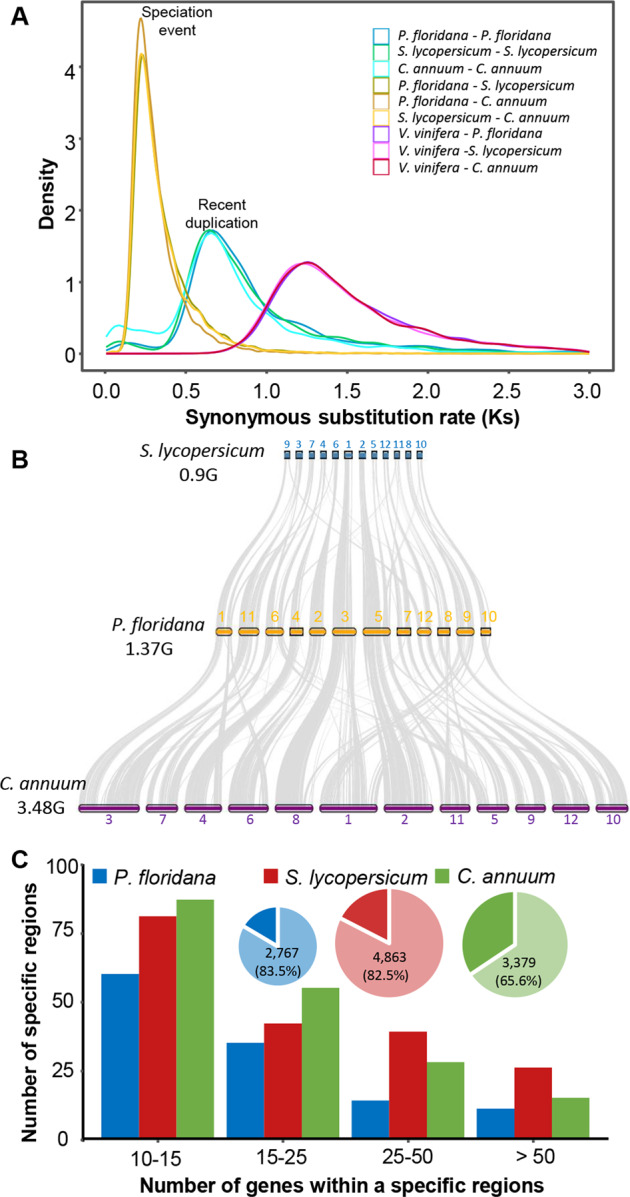
Genome evolution of solanaceous species. **A** Synonymous substitution rate (Ks) distributions of syntenic blocks for *P. floridana* paralogs and orthologs with other eudicots are indicated by colored lines. **B** Synteny analyses of three closely related genomes of *S. lycopersicum*, *P. floridana* and *C. annuum*. Lines between chromosomes indicate syntenic regions. Gray wedges in the background highlight syntenic blocks between two species. **C** Identification of genomic regions with *Physalis*-specific gains or losses compared with *Solanum*. Most genes within one nonsyntenic region have no orthologs in another species, suggesting that these genes have been lost. The numbers and ratios of these lost genes in each species are indicated in corresponding pie charts with a light color

To understand the whole-genome duplication (WGD) history of *P. floridana*, we investigated the distribution of the synonymous substitution rate (*Ks*) between syntenic gene pairs based on comparisons among *P. floridana, C. annuum*, *S. lycopersicum*, and *Vitis vinifera*. The *Ks* peaks of paralogous syntenic gene pairs in *P. floridana*, *C. annuum*, and *S. lycopersicum* were ~0.65, which was greater than the *Ks* peak of Solanaceae speciation (~0.21) and less than that of the Vitis-Solanaceae divergence (~1.25) (Fig. 3A). In intragenomic syntenic comparisons of *P. floridana* and *S. lycopersicum*, both showed clear 1:2 patterns (Supplementary Fig. 15A, B), suggesting a whole-genome triplication (WGT) event in the evolutionary history. Further comparison of the genomes of *P. floridana* and *V. vinifera* showed a clear 3:1 syntenic ratio, indicating that *P. floridana* WGT occurred after divergence from *V. vinifera* (Supplementary Fig. 15C). Moreover, syntenic comparisons clearly showed a 1:1:1 relationship among the *P. floridana*, *C. annuum*, and *S. lycopersicum* genomes (Fig. 3B and Supplementary Fig. 15D). All of these observations highlighted the occurrence of a recent WGT event before the divergence of Solanaceae species and after the core eudicot common hexaploidization event^31,32,38^.

Given the common WGD history of Solanaceae species, we further investigated genome structural evolution following the speciation of *P. floridana*, *S. lycopersicum*, and *C. annuum* (Fig. 3B). A clear one-to-one syntenic relationship among the three genomes was detected at the whole-chromosome level, and the overall gene collinearity among the three genomes was largely conserved, although their genome size varied (Fig. 3B and Supplementary Fig. 16), implying that these species did not experience large amounts of chromosome fusion or recent WGD events after species divergence. Specifically, we identified 367 and 529 large syntenic gene blocks in the *P. floridana* genome compared with *S. lycopersicum* (occupying 78% *Physalis* genes) and *C. annuum* (occupying 60% *Physalis* genes), respectively (Supplementary Table 13). Therefore, the genomes of three Solanaceae species were relatively conserved; only several small-scale chromosomal rearrangements and specific genomic regions were observed (Fig. 3B, C).

We investigated these nonsyntenic genomic regions among the three studied Solanaceae genomes as regions likely to provide insights into their specific genomic evolutionary history. In total, we found that 145 *P. floridana* genomic regions containing 3,314 genes of *P. floridana* and 211 *S. lycopersicum* genomic regions, including 5,891 genes and 226 *C. annuum* genomic regions comprising 5,149 genes, were nonsyntenic (Fig. 3C). Furthermore, an all-against-all BLAST search revealed that 2,767/3,314 genes in these nonsyntenic regions in *P. floridana* did not identify orthologous genes in the genomes of *S. lycopersicum* or *C. annuum* (Supplementary Table 14), indicating that they were likely *P. floridana-*specific genes. Notably, we found that several steroid biosynthesis-related squalene epoxidase (SQE) genes were located in these nonsyntenic regions in *P. floridana* and seemed to be specifically expanded in *P. floridana* (Supplementary Table 14). More strikingly, a few floral development-associated MADS-box genes were located in the *S. lycopersicum-*specific nonsyntenic regions (Supplementary Table 15), likely indicating key gene losses through segmental deletion in the *P. floridana* and *C. annuum* genomes.

### Evolutionary analyses of gene families

**Fig. 4:**
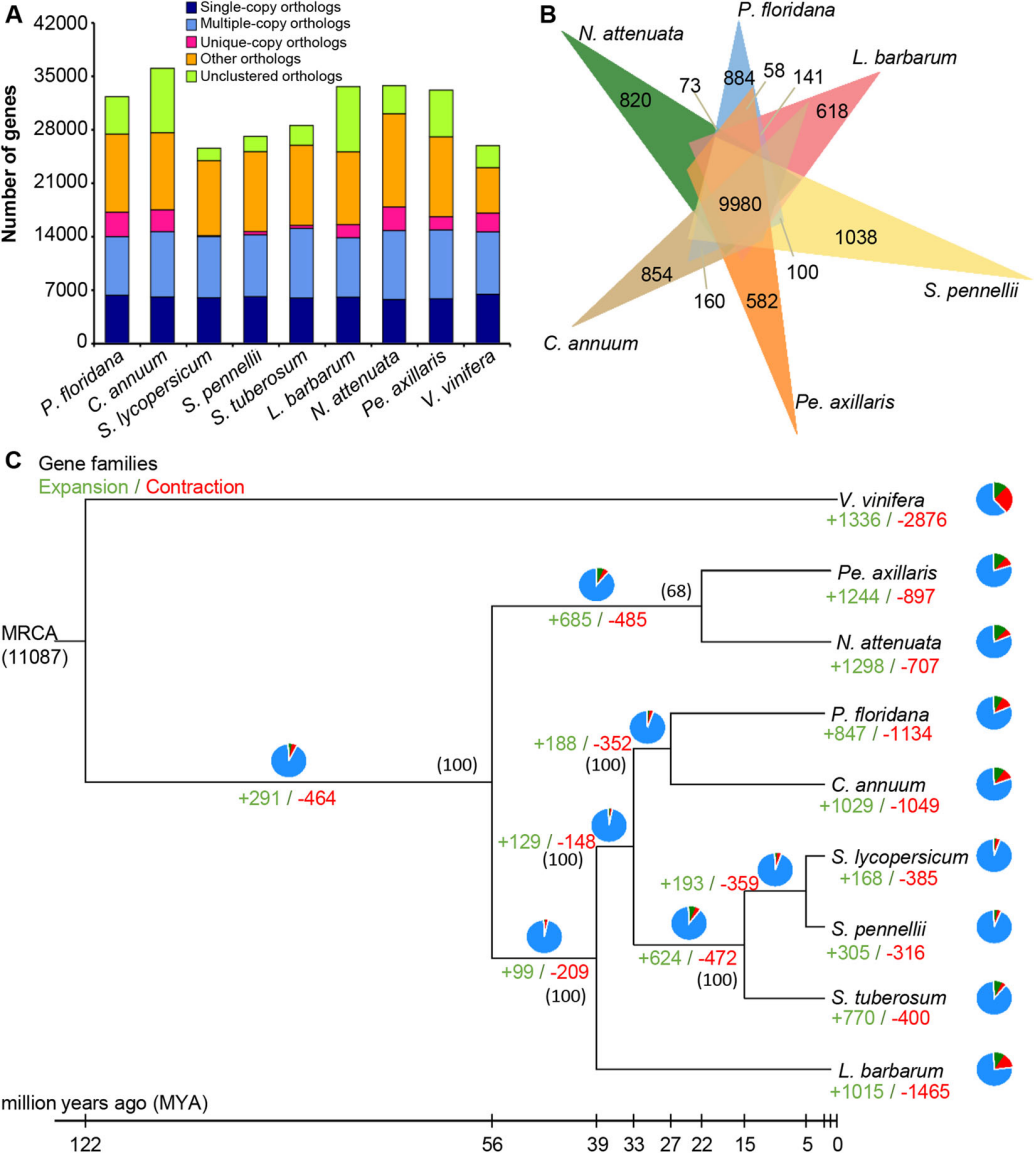
Gene family comparisons among representative angiosperm species. **A** Distribution of single-copy orthologs (dark blue), multiple-copy orthologs (pale blue), unique genes (red), other paralogs (orange), and other unclassified genes (green) in *P. floridana*, *C. annuum*, *S. lycopersicum*, *S. tuberosum*, *S. pennellii*, *L. barbarum*, *N. attenuata*, *Pe. axillaris*, and *V. vinifera*. **B** Venn diagram showing unique and shared gene families among *P. floridana* and the indicated species. The numbers of orthologous gene families shared by all species, gene families specific to each species, and specifically shared gene families between the *P. floridana* and other Solanaceae genomes are given. **C** Gene family expansion and contraction across a phylogenetic tree of 9 plant species. The number at the root (11,087) represents the number of gene families in the most recent common ancestor (MRCA). Bootstrap support numbers are given in parenthesis. Green numbers indicate the number of gene families that expanded in a species relative to the closest species during evolution, and red numbers indicate the number of gene families that contracted. The pie charts on the right show the proportions of these categories (green, expanded; red, contracted; blue, unchanged.)

Comparisons among multiple genomes can reveal the distribution of orthologous genes and the expansion and contraction of gene families. We first focused on gene family analyses during the evolutionary history of Solanaceae using grape as an outgroup. The genomes of eight solanaceous species, including *P. floridana*, *C. annuum*, *S. pennellii*, *S. lycopersicum*, *S. tuberosum*, *Nicotiana attenuata, Petunia axillaris*, and *Lycium barbarum*, together with *V. vinifera*, were employed to construct orthogroups using OrthoMCL. We found that most genes were clustered into orthologs or paralogs among these Solanaceae genomes (Fig. 4A and Supplementary Table 16), and a core set of 9,980 gene families were shared among these genomes (Fig. 4B). In addition, there were 884 families specific to *P. floridana*, 854 gene families specific to *C. annuum*, 820 specific to *N. attenuata*, 582 specific to *Pe. axillaris*, 1,038 specific to *S. pennellii*, and 618 specific to *L. barbarum* (Fig. 4B and Supplementary Table 17). We found 160 gene families specifically shared by *P. floridana* and *C. annuum*, which was greater than the number of specifically shared gene families between the *P. floridana* and any of the other Solanaceae genomes (Fig. 4B), further supporting the notion that *C. annuum* is the closest relative to *P. floridana*.

Expansions and contractions of certain gene families have occurred frequently during evolution. A total of 291 gene families expanded before the divergence of Solanaceae, whereas 464 families were found to have contracted when the representative genomes of six genera (*Physalis*, *Capsicum*, *Solanum*, *Nicotiana*, *Lycium*, and *Petunia*) within Solanaceae were included (Fig. 4C). Moreover, 847 gene families were expanded (360 by a significant margin, *P* ≤ 0.05), while 1,134 gene families were contracted (57 by a significant margin, *P* ≤ 0.05) in *P. floridana* (Supplementary Tables 18 and 19). We conducted Gene Ontology (GO) and Kyoto Encyclopedia of Genes and Genomes (KEGG) enrichment analyses of the 3,797 genes in the 360 significantly expanded gene families in *Physalis*. GO categories including flower morphogenesis, terpenoid biosynthetic process, and cytokinin metabolic process were significantly enriched in these expanded gene families (Supplementary Fig. 17A and Supplementary Table 20), while pathways including zeatin biosynthesis, steroid biosynthesis (squalene monooxygenase), sesquiterpenoid and triterpenoid biosynthesis, and brassinosteroid biosynthesis (CYP450 85A3-like proteins) were significantly enriched in the KEGG analysis (Supplementary Fig. 17B and Supplementary Table 21). Among the 57 significantly contracted gene families, we found that GO categories including plant−type hypersensitive response, proteolysis, signal transduction, and low−affinity nitrate transport (nitrate/nitrite transporter) were significantly enriched (Supplementary Fig. 18A and Supplementary Table 22), while pathways including plant hormone signal transduction, linoleic acid metabolism, and isoflavonoid biosynthesis were significantly enriched in the KEGG analysis (Supplementary Fig. 18B and Supplementary Table 23).

To further reveal the uniqueness of the *Physalis* genome, we constructed orthogroups using *P. floridana* with 12 other sequenced plant species. We found that orthologous genes, paralogous genes, and nonclustered genes were distributed in a similar manner among the 13 compared plant species (Supplementary Fig. 19). In total, 27,115 annotated protein-coding genes were classified into 15,724 gene families, 1,013 of which were *P. floridana* species specific relative to other investigated species (Supplementary Tables 24 and 25). We next conducted a GO enrichment analysis of the 1,013 *Physalis*-specific gene families and found that these genes participated in multiple biological and metabolic processes (Supplementary Fig. 20 and Supplementary Table 26), suggesting diverse roles of the *Physalis*-specific genes. By examining these genes, we found that some gene families of MADS-box genes and steroid biosynthesis-related genes were unique to *P. floridana*. Additional and unique copies of a few MADS-box genes, including several families from each of the AGL61-like, AGL80-like, *Arabidopsis* nitrate regulated 1 (ANR1)-like, MADS50-like, SOC1-like, and AGL29-like gene families, were found in *Physalis* (Supplementary Table 25), perhaps as a result of unequal gene duplications or losses among different species. Four steroid biosynthesis-related gene families were identified: gene family 12781 (12 genes) and gene family 24583 (2 genes), encoding SQE, as well as gene family 20969 (3 genes) and gene family 25195 (2 genes), related to brassinosteroid biosynthesis (Supplementary Table 25). In accordance with this, KEGG enrichment analysis revealed that “steroid biosynthesis” was the most significantly enriched pathway in *P. floridana* (Supplementary Fig. 21). Eleven of 35 steroid biosynthesis (map00100) pathway-related genes were significantly enriched, and 10 of the 11 genes were *SQE* genes (Supplementary Table 27).

A higher dN/dS ratio (>1) indicates positive Darwinian selection on proteins, which may thus have undergone more rapid protein evolution. We therefore performed this analysis at the genomic level, revealing 127 positively selected (d*N*/d*S* > 1, Branch-site-specific model M2A, *p* < 0.05) genes in the P106 genome, including genes encoding zinc finger, ribosomal, ABC transporter, cytochrome P450, small GTPase superfamily, and glycoside hydrolase family proteins (Supplementary Table 28), suggesting that positive selection on these genes during evolution might be involved in *Physalis* plant responses to different stresses. The pf02G034050 (GO: 0006694, *p* ≤ 0) and pf03G074230 (GO: 0016126, *p* ≤ 9e-06) genes, related to steroid biosynthetic processes, were found to be positively selected (Supplementary Table 28). Some steroid compounds isolated from *Physalis* plants contain chloride ions^39^ that are thought to be formed by reaction with an epoxide group^21^. Consistent with this, the positively selected gene pf03G066370 (GO: 0006821, *p* = 0) was found to be a chloride transport-related gene (Supplementary Table 28). These results suggest a role of these positively selected genes related to steroid biosynthesis.

Through the above evolutionary analyses at multiple levels, we found that the solanaceous genomes are conserved overall; however, small-scale mutations were also observed among the genomes of *Physalis*, *Capsicum*, and *Solanum*. The evolutionary consequences of these genetic variations deserve further functional investigation. Here, potential genetic variations of genes related to key steps in the biosynthetic pathways of steroid-related compounds, especially the MADS-box genes involved in Chinese lantern formation, were of primary interest to us and were thus further explored.

### Evolution of genes involved in steroid compound biosynthetic pathways

**Fig. 5:**
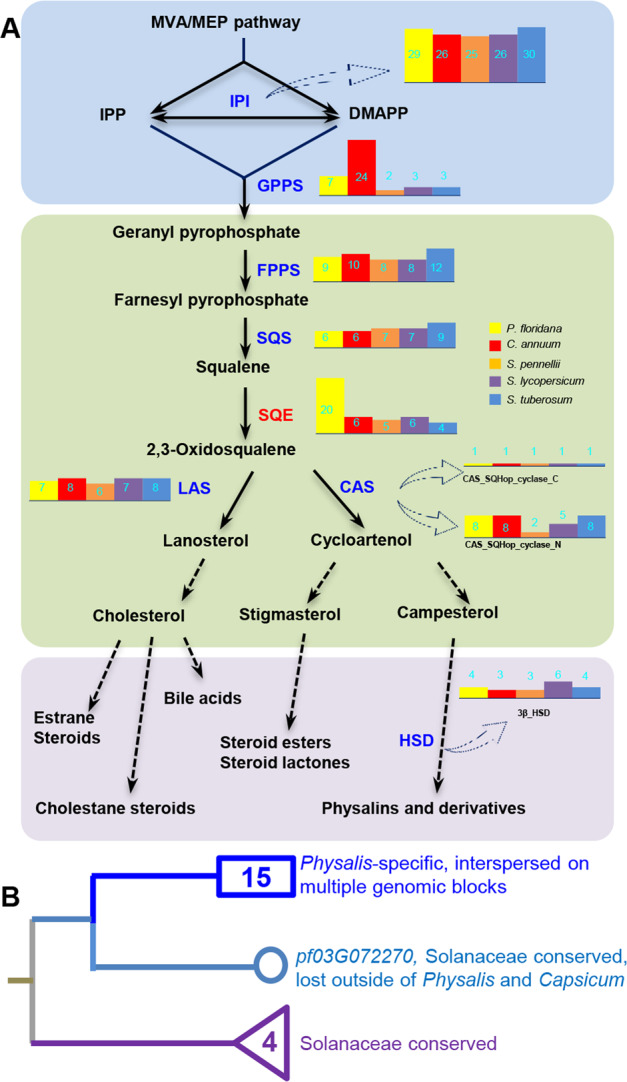
Specific evolution of genes involved in steroid-related derivative biosynthesis in *Physalis*. **A** A simplified steroid-related derivative biosynthetic pathway. The solid arrows indicate the confirmed sections, while the dashed arrows represent the proposed sections. The examined enzymes are given, and the gene numbers in different species are indicated by the histogram. MVA, mevalonic acid; MEP, methylerythritol 4-phosphate; IPI, isopentenyl pyrophosphate isomerase; GPPS, geranyl diphosphate synthase; FPPS, farnesyl diphosphate synthase; SQS, squalene synthase; SQE, squalene epoxidase; CAS, cycloartenol synthase; LAS, lanosterol synthase; HSD, 3-beta hydroxysteroid dehydrogenase/isomerase. **B** A schematic diagram displaying the origin and expansion of *SQE* genes in *P. floridana*. The blue square represents 15 *Physalis*-specific *SQE* genes that expanded from a conserved Solanaceae *SQE* (pf03G072270) but were lost in *Solanum*. The triangle represents the other four Solanaceae-conserved *SQE* genes. Detailed information is available in Supplementary Figs. 22 and 23

Steroids are essential for all eukaryotes^25,40,41^. Withanolides are a class of polyoxygenated steroids based on a C_28_ ergostane skeleton; they are the most abundant steroids found in the genus *Physalis*^24,26^. This is a striking characteristic of chemical diversity in *Physalis* fruits, and the genes in the biosynthetic pathways of steroid-related compounds were repeatedly captured in the above analyses. We therefore checked the key genes in the entire steroid biosynthetic pathways^27–29,42^, which were shown in a simplified framework (Fig. 5A). Thirty-three gene families, including those encoding the rate-limiting enzymes 3-hydroxy-3-methylglutaryl-coenzyme A (HMGR) and SQE, were checked in the related genomes. We found that the copy-number variation (CNV) of most gene families was comparable among the species of *Physalis*, *Capsicum*, and *Solanum*. In contrast, significant changes in the CNV of annotated *HMGR* and *SQE* genes (Fig. 5A and Supplementary Table 29) showed direct expansion of these genes in *Physalis*. In particular, a total of 20 *SQE*-like genes were found in *P. floridana* (P106), and the copy number was ~4-fold higher than that in the non-*Physalis* genomes examined (Supplementary Table 30). These SQEs showed *Physalis*-specific expansion (Fig. 5B). In addition to the conserved *SQE*-located regions among Solanaceae, these expanded *SQE* genes were mainly located in *Physalis-*specific regions (Supplementary Fig. 22), and most of them shared high homology with *pf03G072270*, which might be restricted to *Physalis* and *Capsicum* (Supplementary Fig. 23), indicating that these expanded *SQE* genes may have been derived from *pf03G072270*. Moreover, most *Physalis*-specific *SQE*s belonged to the two subfamilies of this *P. floridana* (*PfSQE*) gene family (24583 and 12781) identified in the gene expansion analysis (Supplementary Fig. 23 and Supplementary Table 18). SQE genes encode flavoprotein monooxygenases that act as rate-limiting enzymes in the steroid biosynthetic pathway^41^, and the roles of the CNV of these genes in the natural variation of steroid metabolites among different species related to *Physalis* need further investigation. However, genetic variations related to MADS-box genes involved in the formation of the Chinese lantern, the most striking novel morphological trait in *Physalis*, were explored in more detail from functional and molecular perspectives.

### *MBP21* loss might have contributed to the origination of the Chinese lantern in *Physalis*

**Fig. 6:**
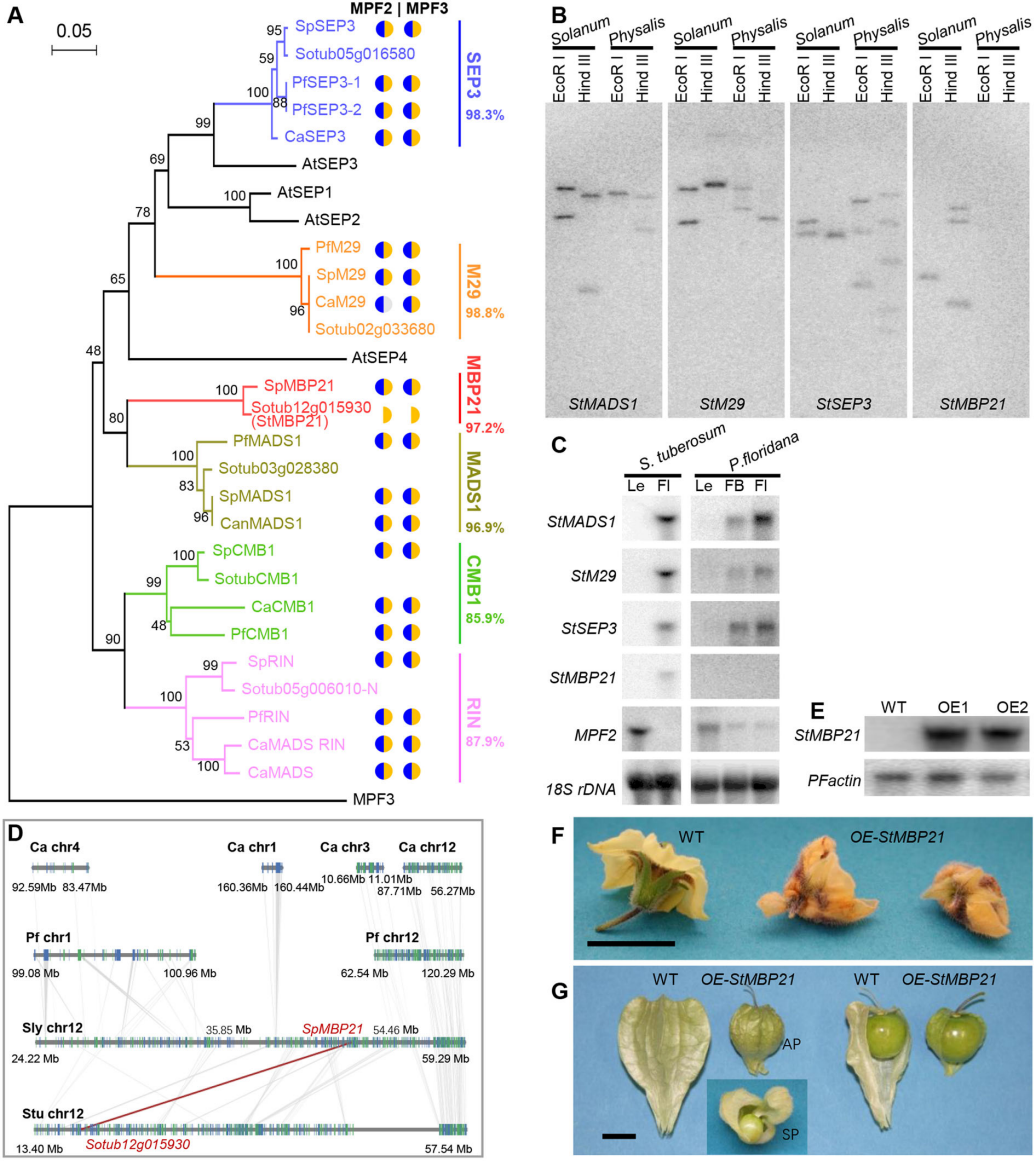
*MBP21*-like MADS-box gene loss and Chinese lantern development in *Physalis*. **A** The phylogeny of *SEP*-like MADS-box genes from the indicated plant species. Different subclades are highlighted with different colors. The circles represent the interactions between MPF2 (left)/MPF3 (right) and the corresponding SEP-like proteins. Blue represents the interaction signals detected in yeast two-hybrid assays. Yellow represents the fluorescent signals detected in BiFC assays. Gray indicates no detectable interaction signal. White indicates not analyzed. The percentage at the bottom of each subclade is the sequence identity of amino acids among the sequences from the four species. Pf, *P. floridana;* Ca, *C. annuum*; Sl, *S. lycopersicum*; St, *S. tuberosum;* At*, Arabidopsis thaliana*. **B** Southern-blotting analyses of *MBP21* genes between *Solanum* and *Physalis*. Total DNA was isolated from *S. tuberosum* and *P. floridana* and digested with *EcoR* I and *Hind* III. **C** The expression of the indicated genes in *Solanum* and *Physalis* revealed by northern-blotting analysis. Total RNA was isolated from the leaves (Le), floral buds (FB), and flowers (Fl) of *S. tuberosum* and *P. floridana*. MADS-box gene-specific probes from *S. tuberosum or P. floridana*, as indicated, were used. **D** Microsynteny of the *MBP21* subclade-containing region among the four indicated species from *Solanum*, *Capsicum*, and *Physalis*. The brown linear region represents the syntenic gene blocks of the *MBP21* gene, and the gray linear region represents the syntenic regions of genes near the target gene. **E** *StMBP21* expression in *OE-StMBP21 P. floridana* plants. Total RNA was isolated from the flowers of two independent transgenic lines and subjected to northern-blotting analyses. The indicated gene-specific probes were used. **F** Flower morphology. Compared to wild-type (WT) flowers, flowers from *StMBP21-*overexpressing transgenic plants (*OE-StMBP21*) were withered, implying that fertilization was inhibited. **G** Fruit morphology comparison. Wild type, WT; *StMBP21*-overexpressing transgenic *Physalis* plants, *OE-StMBP21*. Self-pollination (SP) occasionally occurred in the *OE-StMBP21* flowers, and artificial pollination (AP) produced berries of similar size to the WT, indicating that female fertility was normally functional and that male function was reduced in *OE-StMBP21* plants. Part of the Chinese lantern was removed to show the berry. Bar = 1.0 cm in (F, G)

The role of MADS-box genes in Chinese lantern formation was reported previously, and these genes include *MPF2* and *MPF3* and possibly their MADS-domain protein-interacting factors^5,14,43^. Genome sequencing showed that the *P. floridana* genome contained 136 putative MADS-box genes, including 92 type I and 44 type II genes (Supplementary Table 31). Mγ in the type I class was significantly multiplied in *P. floridana*, but both MIKC and MIKC* in the type II class appeared to have been phased out in *P. floridana* relative to *Solanum* (Supplementary Table 31). The copy or subclade numbers of most MIKC genes were generally invariant among solanaceous species; however, several showed changes, including *AGL12*, *FLOWERING LOCUS C* (*FLC*), and *SEPALLATA* (*SEP*)-like genes (Supplementary Fig. 24 and Supplementary Table 31). No evidence supports a putative role of other lost or expanded MADS-box genes, such as *AGL12* or *FLC*, in Chinese lantern formation; these genes play roles in flowering time control and root meristem development in *Arabidopsis*^44,45^. However, SEP-like proteins in *Physalis* or *Arabidopsis* dimerize with MPF2 or MPF3, and the loss of a certain SEP-like interacting protein is correlated with sepal inflation^16,43^. In line with these results, a genomic region harboring a *SEP*-like gene was identified as having been lost in *Physalis* relative to *Solanum* (Fig. 3C and Supplementary Table 15). Therefore, the genomic variation related to *SEP*-like loss might be essential in the origin of ICS. To obtain further evidence supporting this assumption, we investigated *SEP*-like genes in Solanaceae. Six putative *SEP*-like MADS-box genes were isolated from *P. floridana*, *S. pimpinellifolium*, and *C. annuum*, and the full length of *CaCMB1* was significantly shorter than those of other *SEP*-like MADS-box genes (Fig. 6A and Supplementary Table 32). The encoded proteins were mainly localized in the nucleus (Supplementary Fig. 25). Moreover, we further demonstrated that nearly all SEP-like MADS-box proteins from the three species and StMBP21 from *S. tuberosum* interacted with MPF2 and MPF3 in both yeast two-hybrid and bimolecular fluorescence complementation (BiFC) analyses (Fig. 6A and Supplementary Figs. 26 and 27), further hinting at their roles in ICS formation. However, the observed copy number is evidence against *SEP*-like gene loss in *Physalis*.

To clarify this issue, we investigated the evolutionary relationships of *SEP*-like genes. A protein sequence-based phylogenetic tree was constructed using the neighbor-joining (NJ) method (Fig. 6A). The *SEP*-like genes in *S. tuberosum* were analyzed *in silico*, and it was noteworthy that Sotub05g006010 contained two sections, each with a MADS-domain and a K domain (Supplementary Fig. 28A). The phylogenetic analysis revealed that Sotub05g006010-N fell into the SEP-like (RIN) group and that Sotub05g006010-C was an A-class MADS-box protein (ortholog of LeMADS-MC and MPF3) (Supplementary Fig. 28B). These findings are in line with the observations in tomato and *Physalis* that the *LeMADS RIN*–*LeMADS MC* and *PfRIN*–*MPF3* orthologs show good maintenance of microsynteny^14,46^. The phylogenetic tree suggested that solanaceous *SEP*-like genes were divided into six subclades, and the sequence identity of each subclade varied in a phylogeny-dependent manner (Fig. 6A). One gene from each of the M29, MADS1, and CMB1 subclades was maintained in all examined solanaceous species. One duplication in the SEP3 subclade of *P. floridana* and one duplication in the RIN subclade of *C. annuum* were found. However, no genes in the MBP21 subclade were found in *P. floridana* or *C. annuum* (Fig. 6A). Nevertheless, one-to-one orthologs of *MPF2-like* and *MPF3* genes existed in all examined solanaceous species (Supplementary Fig. 29), suggesting the specific loss of the *MBP21* subclade from the *SEP*-like gene family in *Physalis* and *Capsicum*.

To verify this hypothesis, we examined the existence of the four closely related *SEP*-like genes in *S. tuberosum* and *P. floridana* via gel-blotting analyses since the variation in sequence identity allowed us to generate subclade-specific probes (Supplementary Table 33). Southern-blotting analyses did not detect the *MBP21* subclade in the *Physalis* genome but did detect it in *Solanum*, while the other three genes were detected in both species (Fig. 6B). Northern-blotting results suggested that the *MBP21* subclade was florally expressed in *Solanum*, whereas it was not detected in *Physalis*, and the other three closely related genes were expressed in the floral organs of both species (Fig. 6C). Microsynteny analysis was further performed for each subclade, and the results showed that microsynteny differed among each subclade in the four examined solanaceous species. Most subclades maintained perfect synteny, as observed in the MADS1, RIN, M29, CMB1, and SEP3 subclades (Supplementary Figs. 30–34), but the extent of the conservation of the synteny of each subclade seemed to be independent of phylogenetic relationships (Fig. 6A). In the SEP3 subclade, *PfSEP3-1* was located close to *PfSEP3-2* (Supplementary Fig. 34), implying that these two *Physalis* genes might have resulted from a tandem duplication. In the *MBP21* subclade, the *MBP21-*orthologous genes were located in syntenic blocks on *S. lycopersicum* chromosome 12 (chr12) from 24.22 to 59.29 Mb and *S. tuberosum* chromosome 12 from 13.40 to 57.54 Mb (Fig. 6D). However, the corresponding orthologous regions in *P. floridana* and *C. annuum* were separated and were located on different chromosomes, indicating that significant genomic rearrangements occurred in *P. floridana* and *C. annuum* (Fig. 6D). Notably, we found that no orthologous syntenic region in *P. floridana* matched the region of chr12 from 35.85 to 54.46 Mb in *S. lycopersicum*, and 81% of the orthologous genes (216/264) in this region of *S. lycopersicum* were lost in *P. floridana* and in *C. annuum*, which included a key floral development gene, *MBP21* (Supplementary Table 34). To confirm *MPB21* gene loss, we further conducted homology searches against all Illumina sequencing data from *P. floridana* and against the entire genomic sequences of *C. annuum*, using the *SlMBP21* coding sequence as a query. We found no valid hits in any of the searches, and we therefore excluded the possibility of genomic misassembly (Supplementary Table 35). These results confirm the loss of the *MBP21* gene in *P. floridana* and *C. annuum* and imply that the loss event might have resulted from chromosomal rearrangements during evolution.

### Functional conflict between *MPF2* and *MBP21* in *Physalis*

The downregulation of the *MBP21* gene in *Solanum* as well as its homologs contributes to inflated sepal development^47–50^, and the failure to detect protein–protein interactions (PPIs) of MPF2-like with certain SEP-like proteins is associated with the loss of the Chinese lantern or enlarged sepals^16,43^, suggesting a role of the *SEP*-like gene family in the evolution of the Chinese lantern. Overall, the *Physalis SEP*-like genes shared similar expression patterns with their counterparts in *Solanum* and *Capsicum* during flower and fruit development (Supplementary Fig. 35); however, virus-induced gene silencing (VIGS) analyses revealed a highly redundant role of *Physalis SEP*-like genes. Organ size (flower, fruit, ICS, and seed) and seed number per berry were decreased in the *PfMADS1* knockdown plants (Supplementary Figs. 36 and 37). Furthermore, two transgenic *Physalis* lines overexpressing *StMBP21* were generated and showed poor male fertility (Fig. 6E, F). Self-pollination occasionally occurred, and artificial pollination with wild-type pollen could produce fruits, but the resulting fruits had abnormal lanterns with small berries or small lanterns that tightly enveloped the berry (Fig. 6G), indicating that the coexpression of *MPF2* and *MBP21* genes affected male sterility and calyx growth in *Physalis*. Such a functional conflict implies that coordination between the two genes is required for their proper function in ICS development.

### *MBP21* loss is correlated with ICS occurrence within Solanaceae

**Fig. 7:**
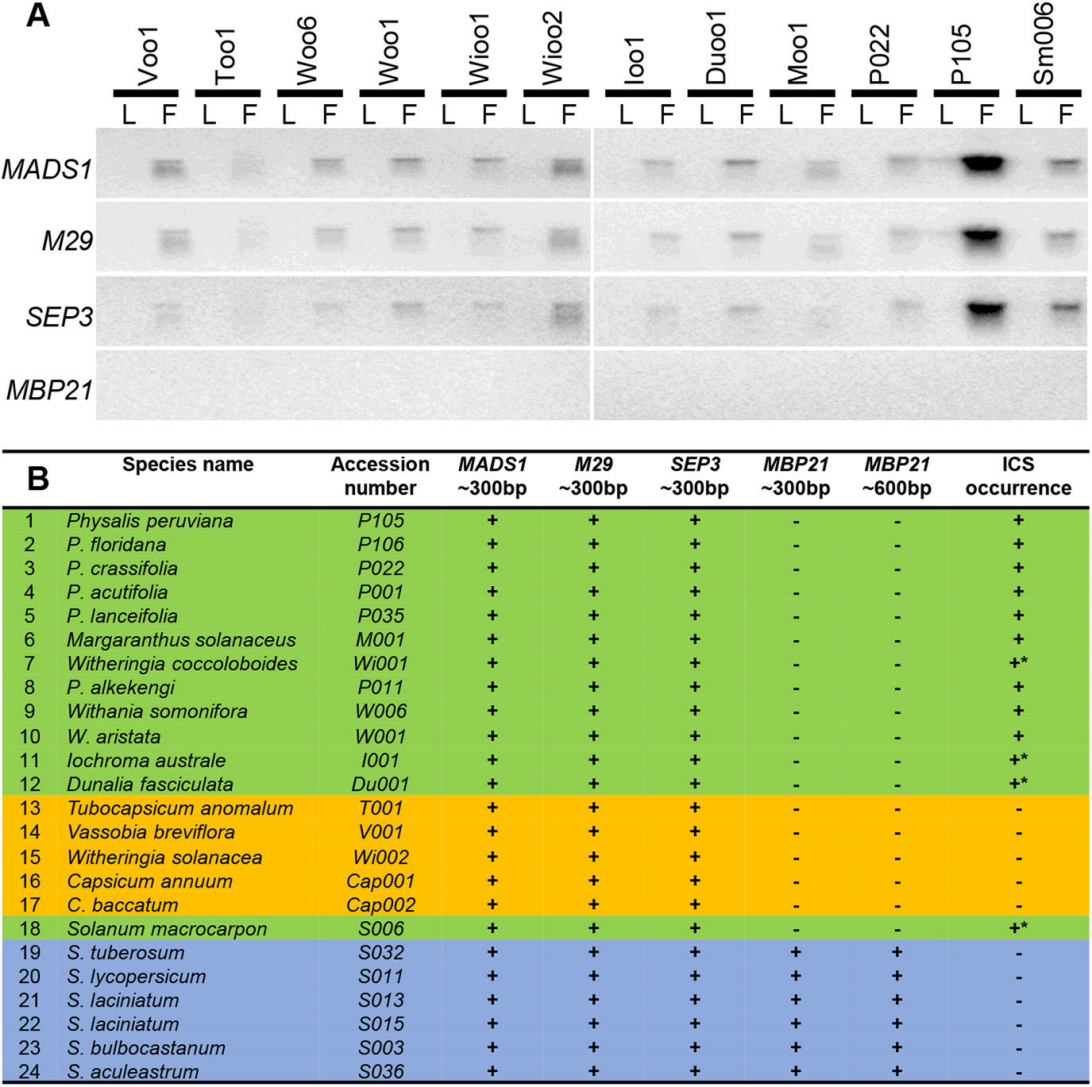
*MBP21* loss correlated with ICS occurrence in Physaleae. **A** Northern-blotting analyses. Total RNA was isolated from the leaves (L) and flowers (F) of the 13 indicated species. **B** Southern-blotting analysis. Genomic DNA of the indicated 24 species was digested by *EcoR* I. *SEP*-like MADS-box gene-specific probes of the *MADS1*, *M29*, *SEP3*, and *MBP21* subclades from *S. tuberosum* were used. +, presence of the indicated genes or the calyx accrescence/ICS; -, not detectable, or the gene or ICS trait has been lost; *, accrescent or enlarged leaf-like sepals. Overall, *MBP21* loss was correlated with ICS occurrence (marked in both green and blue). The observed exceptions (highlighted in orange) could be explained by secondary mutations in ICS biosynthesis pathways^10,15^

To identify the correlation of *MBP21* loss and ICS occurrence within Solanaceae, northern and Southern-blotting analyses were conducted in additional species; the results further indicated the possible loss of *MBP21*-orthologous genes in Physaleae (Fig. 7), in which the ICS trait occurs frequently^10,17^. The gene existed in most *Solanum* species lacking ICS but was lost in the species with inflated leaf-like sepals (Fig. 7), reinforcing the essential role of *MBP21* loss in calyx accrescence and inflation. The *MBP21* gene was also found in other representative solanaceous genomes, such as those of *Lycium*, *Petunia*, and *Nicotiana* (Supplementary Fig. 38), whose species do not exhibit ICS. Thus, *MBP21* loss was correlated with ICS-like occurrence overall but with a few exceptions, including the *Capsicum*, *Vassobia*, and *Tubocapsicum* genera (Fig. 7), which are explicable^10,15^. These observations again support a role of *MBP21* loss in the origin of ICS within Solanaceae.

Taken together, the results indicate that the loss of the *MBP21* subclade likely represented a novel mutation involved in the origin of the Chinese lantern.

## Discussion

### The first high-quality *Physalis* genome assembly

*Physalis* is quickly becoming an important genus within Solanaceae in the context of the current focus of the plant sciences, not only because of its iconic fruit morphology but also due to its unique biochemical profile of steroid metabolites^3,6^. The genus has drawn attention from both evolutionary biologists and berry breeders. No complete genome of *Physalis* has been made publicly available to date, thus hampering relevant evolutionary genetic research and breeding efforts. Here, we report the genome of *P. floridana*, providing data on a conserved representative of *Physalis* species that originated in the New World. The genome size of *P. floridana* is ~1.40 Gb, and the 12 assembled pseudochromosomes covered ~98.82% of the genome. The number of protein-coding genes was predicted to be 32,075, comparable to the numbers in *Solanum* and *Capsicum*^30–33^. Nevertheless, the genome size and gene content varied, possibly due to small-scale gene duplications, TE variations, and genome rearrangements after species divergence. Among the reported solanaceous genomes, the current genome of *P. floridana* was the first to be deciphered by using PacBio sequencing combined with Hi-C technology, where the latter method overcomes the limitations imposed by the lack of genetic maps for genome assembly^51^. The assembled genome of *P. floridana* exhibits higher chromosome-level completeness (98.82%) than those of *C. annuum* (86%), *S. lycopersicum* (84%), *S. pennellii* (97%), and *S. tuberosum* (86%)^30–33^ as well as greater contiguity, with an N50 of ~4.87 Mb, and was further anchored onto pseudochromosomes. Considering the novelty, contiguity, accuracy, and completeness of our sequences, our genome constitutes the first available high-quality genome of *Physalis*. The data should be able to promote further genetic and genomic studies of *Physalis* species and other solanaceous plants.

Through comparative genomic analyses at different levels, we characterized genomic structural variations, *Physalis*-specific genes, expanded and contracted gene families, and positively selected genes to understand the novel genetic variation occurring in the *Physalis* genome relative to the genomes of *Solanum* and *Capsicum*, although the gene collinearity of the solanaceous genomes was conserved overall. We found that some gene families were repeatedly detected in these analyses; many varied in either copy number or the coding sequences that could be associated with the development and evolution of *Physalis* from various aspects. For example, the special expansion of ANR1-like MADS-box genes and the significantly enrichment of low−affinity nitrate transport (nitrate/nitrite transporter) among the contracted gene families may be related to the fact that *Physalis* plants are adapted to poor soil conditions^52,53^. The enriched hormone-related genes (i.e., those involved in cytokinin metabolic processes in *Physalis*-specific gene families) were in line with the observation that Chinese lantern development is triggered by fertilization/hormonal signals^5,11^. However, these genetic variations observed in *Physalis* need further extensive investigation through comparative and functional analyses among solanaceous species to understand their evolutionary significance. In this work, we focused on the evolution of *SEP*-like MADS-box genes and *SQE* genes and their potential impacts on both the morphological and biochemical evolution of the novel fruit traits of *Physalis*.

### On the origin of the Chinese lantern, a morphological novelty within Solanaceae

Consecutive genomic mutations are believed to drive the process of morphological evolution. Gene copy-number gains and losses and variation in the spatiotemporal expressional pattern or dosage impacts are crucial mutational events in evolution^3,8,45,54^. The pioneering hypothesis regarding the genetic basis of Chinese lantern formation was inspired by the *tunicate* mutants of *Zea mays*^8,55,56^, in which the ectopic expression of a *ZMM19*-like MADS-box gene caused an increase in sepal size^8^. In *Physalis*, *MPF2*, a closely related homolog of *ZMM19*, was characterized as playing a role in the development of a novel trait based on RNA interference (RNAi)-mediated silencing^5^ and overexpression approaches^8,11^. The evolutionary genetic mutation related to the heterotopic expression of an *MPF2-like* gene from vegetative to floral contexts is due to *cis*-element variation in the promoter of this gene^5,14,57^. We further demonstrated that *MPF2* is involved in calyx organ identity and size control together with *MPF3*, and the role of MPF2 in increasing calyx size is dependent on the expression of normal *MPF3*^14^. The knockdown of both *MPF2* and *MPF3* also affects male fertility^5,14^. Heterotopically expressed *MPF2* was demonstrated to establish PPIs with proteins integral to floral pathways such as PFMAGO, MPF3, AG, and SEP^43^. The role of *MPF2*-like variation in ICS formation seems to be conserved in Solanaceae^15,16,58^. Within Solanaceae, the heterotopic expression of *MPF2*-like genes is a plesiomorphic trait^10^, and mutations in MPF2-related PPIs affect the evolution of ICS^16,43^. Among these mutations, *SEP*-like variation is likely essential for Chinese lantern formation, since the knockdown of several *SEP*-like genes in *Solanum* led to enlarged sepals despite their major roles in floral meristem determination^47–50,59^. In this work, through genome comparisons and various experimental approaches, we revealed the evolutionary trajectory of the *SEP* genes within Solanaceae and confirmed the loss of the *MBP21* subclade in *Physalis* and *Capsicum*. Moreover, the *MBP21* subclade may have been lost in Physaleae, a lineage grouping most species exhibiting ICS within Solanaceae. *SEP*-like genes are also essential for fertility in various plants^47,60,61^. We further showed that overexpressing *StMBP21* in *Physalis* led to poor male fertility and small lanterns, resembling the floral phenotypic variation observed in *MPF2*-RNAi transgenic *P. floridana* lines^5^. We therefore inferred that the loss of the *MBP21* subclade was a major event giving rise to ICS.

**Fig. 8:**
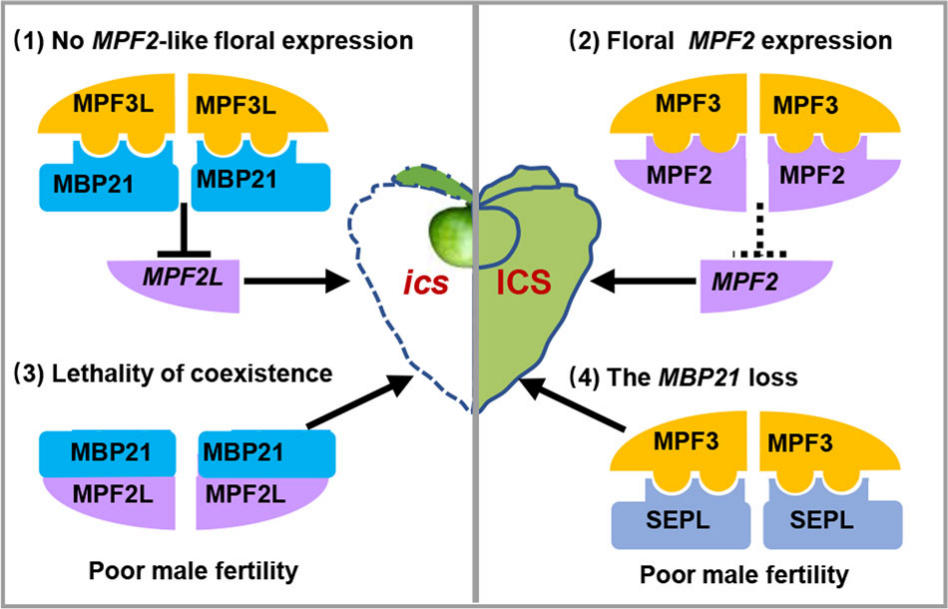
A hypothetical model for the origin of the Chinese lantern. Three genes (*MPF3*, *MPF2*, and *MBP21*) and two mutational events (floral *MPF2* expression and *MBP21* loss) were characterized. Male fertility was integral, and the establishment and variation of the PPIs among these three MADS-domain proteins seemed to be an important way to exert their effects on improving impaired male fertility, thus incidentally giving rise to the origin of the Chinese lantern. (1) and (2) represent the molecular scenarios of non-ICS (*ics*) and ICS phenotypic variation, respectively, within Solanaceae; (3) indicates that the coexpression of *MPF2* and *MBP21* in stamens and calyx led to male sterility and calyx size reduction, while (4) proposes that the loss of *MBP21*, which could be replaced by other SEP-like proteins (SEPL), might have impaired male fertility and promoted calyx inflation. Situation (3) is deleterious and might have been eliminated during floral evolution, while (2) and (4) are two mutual compensatory mutation events co-contributing to the origination of ICS in Solanaceae. The orthologs of these genes from other solanaceous species were defined as *MPF2*-like (*MPF2L*), *MPF3*-like (*MPF3L*), and *MBP21*-like (*MBP21L*). The expression and PPIs in the non-ICS (*ics*) and ICS species are indicated. In the current model, compared to the non-ICS plants (*ics*), the co-occurrence of two mutation events becomes a hallmark of plants with ICS within Solanaceae

All genes related to lantern development characterized thus far have also been found to be required for male fertility^5,14,58^ (this work), leading to the “genetic erosion-restoration of male fertility” hypothesis for the origin of the Chinese lantern. The origin of this structural novelty might have resulted from two genetic changes: *MPF2* floral expression and *MBP21* loss. The overexpression of *MPF2* or *STMADS16* created enlarged leaf-like sepals but did not produce self-fertilized berries in transgenic *S. tuberosum* plants^11^. We are not able to evaluate the effects on male fertility in that case; however, in our *StMBP21* transgenic *Physalis* plants, in which *MPF2* is already expressed^5^, overexpressing *StMBP21* in stamens apparently reduces male fertility, indicating that the combination of *MPF2* and *MBP21* might be deleterious. We further found that MPF2 and MBP21 shared the interaction partner MPF3, an essential gene in calyx development^14^. Moreover, MPF2 was shown to heterodimerize with MBP21. Thus, floral *MPF2* expression could affect the heterodimerization of MPF3 and MBP21, which forms a normally functional dimer in floral development (Fig. 8)^43^, or the MPF2-MBP21 heterodimer could have a deleterious effect on male fertility (Fig. 8). Furthermore, in line with observations made in tomato^48^, we found that SlMBP21 and StMBP21 interacted with MPF3, the *LeMADS*-MC ortholog of *Physalis*^14^. The downregulation of *MBP21* can significantly increase sepal size in tomato^49^; however, the loss of *MBP21* might have caused poor male fertility in the ancestral plants before the origination of ICS (Fig. 8). We thus hypothesized that the expression of either MPF2 or MBP21 in stamens (one only) may have been sufficient to guarantee normal male fertility, and their coexistence in the stamen was harmful; however, a single mutation affecting either floral *MPF2* expression or *MBP21* loss could complement the disadvantageous consequence of the other mutation for fertility so that the two mutations together, incidentally and additively, caused the development of an inflated fruiting calyx (Fig. 8). This also suggests an evolutionary disadvantage of having both genes present in floral organs (i.e., stamens) and an advantage of having only one gene present in stamens. In this scenario, the Chinese lantern might have been a byproduct at the timepoint of its origin, but its adaptive role was selectively maintained during flower evolution thereafter^12,37,55^.

An inflated calyx develops in *S. tuberosum* and *Arabidopsis* upon the overexpression of an *MPF2*-like gene^8,11,15,16,56^. However, the induction of the development of a perfect Chinese lantern, a masterpiece of nature, in a non-ICS plant (i.e., *S. tuberosum*) represents a formidable challenge. Moreover, completely disrupting the formation of the inflated calyx in plants showing ICS (i.e., *P. floridana)* has not yet been achieved^5,58^, even in large-scale mutagenesis studies^62^. First, male fertility is integral to the ICS origin^5,11,43^. Moreover, the Chinese lantern is a complex trait with an intricate biosynthetic pathway^5,11^, and there are multiple genes associated with the trait, whose functional divergence may have differed after species divergence. Unlike the situation in *Solanum*, the disruption of each *SEP* gene could affect floral or fruit development^46–50,63–67^. The knockdown of any single *SEP* gene in *Physalis* did not lead to obvious phenotypic variation, hinting at genetic robustness due to redundancy, compensation, or rewiring in the genetic network. Nevertheless, the overexpression of *StMBP21* in *Physalis* abolished male fertility and repressed ICS development. These findings suggest that the orthologous and paralogous genes could have undergone distinct functional divergence fates and patterns after species divergence, highlighting putative specialized roles of *MBP21* in male fertility and repressing calyx inflation. This gene is apparently not a component of the ICS development program, but its loss during evolution may have been one major mutational event promoting the origination of ICS.

*MPF2-like* floral expression was demonstrated to be plesiomorphic^10^, while *MBP21-like* loss seemed to be mostly restricted to Physaleae. Once the biosynthetic pathway of ICS was established after the two major mutational events, secondary mutations in the pathway could have led to the disappearance of ICS, as observed in species that have lost, as observed in *Capsicum*, *Vassobia*, and *Tubocapsicum*. The secondary mutations affecting ICS evolution are largely underestimated throughout Solanaceae, although a few, including mutations abolishing *MPF2*-like expression in floral organs and polyploidization and subsequent gene loss, have been characterized^10,11,15^. The two mutation events were unlikely to have occurred simultaneously, and we would assume that *MBP21*-like loss might have been the primary mutation, since it could have resulted from genome rearrangements and was likely disadvantageous, while floral *MPF2*-like expression is mainly a consequence of promoter alteration^5,14^. We could not discriminate between a plesiomorphic origin^10^ and a directed acquisition^17^ of ICS, as this would depend on the order and importance of the two identified mutation events (floral *MPF2* expression and *MBP21* loss) and on the exact spatial expression of *MBP21*- and *MPF2-like* genes and their functional characteristics in the floral organs of Solanaceae. The proposed hypothesis for the origin of ICS and its evolutionary pattern might be elucidated once these issues are well-characterized in more representatives of Solanaceae and the corresponding ancestral states are more firmly reconstructed.

### Distinct evolution of steroid biosynthetic pathways in *Physalis* fruits

Steroids are essential triterpenoid compounds that are essential for plant growth, development, and differentiation^25,29^. As a result of adaptive evolution, steroid-derived compounds often play roles in communicating with neighboring plants, attracting pollinators and seed dispersers, and defending against pathogens and herbivores^18,19^ and occasionally function in host-plant specialization^68^. Some steroid derivatives are antinutritional factors that disrupt digestion and nutrient absorption in humans and have thus been reduced or eliminated during crop domestication^42^. However, the steroid biosynthetic pathway is highly conserved and is a key step in eukaryote evolution^27,29,69,70^. Steroids are present in all eukaryotes, and they modulate the fluidity and flexibility of cell membranes^41^. Some steroid compounds exhibit potential anticancer, anti-inflammatory, and apoptotic activities^20,21^. Thus, steroid-based drugs have a broad range of therapeutic applications; consequently, they represent the category of pharmaceuticals with the largest market share^71^. In most plants, steroid components are low in abundance, while members of the Solanaceae family show relatively high steroid synthesis and accumulation and therefore serve as unique models for studying plant cholesterogenesis^29^. *Physalis* species, belonging to Solanaceae, have the ability to oxidize carbons in the steroidal nucleus and the lateral chain, giving rise to a variety of withasteroids, such as withanolides and physalins^39,68,72^. Withanolides (steroidal lactones) are polyoxygenated ergostane derivatives with a lactone group at C_26_, while physalins are C_28_-secosteroid, lactone-type constituents of *Physalis* species^73^. Multiple withanolides and physalins have been isolated from the genus *Physalis*^24,74^. Based on untargeted metabolome analysis, our preliminary analyses suggested that the accumulation of some steroid components of *Physalis* fruits (bile acid derivatives, estrane steroids, physalins and derivatives, steroid lactones, and steroid esters) occurs at higher levels than is observed in *Capsicum* and *Solanum* fruits (Supplementary Fig. 39 and Supplementary Table 36). The identities of these compounds need further investigation. However, most of these compounds can usually be found in herbs and are produced via multiple biosynthesis pathways and from multiple precursors, including the two general C_5_ isoprene units for phytosterol biosynthesis via a cytoplasmic pathway (the MVA pathway), a chloroplastic pathway (the MEP pathway)^27^, and an additional 13 steps of triterpenoid biosynthesis^28,29^. Nevertheless, the biosynthesis and metabolic pathways of steroids and steroid derivatives in *Physalis* are largely unknown, and the events downstream of C_24_ alkyl sterols (phytosterols) are particularly a mystery.

The biosynthesis of steroidal alkaloids and saponins in triterpenoid biosynthetic pathways in solanaceous plants is mainly based on cholesterol^29,42^. We systematically analyzed the genes involved in steroid biosynthesis and metabolic pathways among various species of *Solanum*, *Capsicum*, and *Physalis* and found that most gene families involved were similar in copy number; however, the copy numbers of the rate-limiting enzyme-encoding genes *HMGR* and *SQE* differed among the genera. In particular, *SQE* genes are dramatically expanded in the *P. floridana* genome. The SQE enzyme catalyzes the first oxygenation reaction from squalene to 2,3-oxidosqualene in the pathway, leading to the syntheses of steroids and steroid derivatives^41,75^. Our preliminary experiment involving *PfSQE* knockdown mediated by group-specific VIGS resulted in a dramatic reduction in steroid constituents, including androstane steroids, cycloartanols and derivatives, estrane steroids, and physalins and their derivatives (Supplementary Figs. 40 and 41; Supplementary Table 37), confirming the essential role of this gene family in the biosynthesis of these steroid-related species in *Physalis*. Although the functional divergence of the genes related to each steroid biosynthesis, and their duplication history need further investigation, the specific origin and expansion of the *PfSQE* family is apparently associated with the biochemical diversity of certain steroid-related species in *Physalis* and might be responsible for the natural variation in these steroid derivatives in different solanaceous species. In accordance with the observed chemical variation among solanaceous species (Supplementary Fig. 39 and Supplementary Table 36), only the levels of physalins and their derivatives were consistently reduced and correlated with the expression of the *PfSQE* genes in the VIGS analyses, which was independent of the applied statistical approaches (Supplementary Fig. 41 and Supplementary Table 37), suggesting that the *PfSQE* family may primarily determine the levels of physalins and their derivatives, which are characteristic steroid-related species in Physaleae.

In addition to *Physalis*, the diversification of ergostane structure has also occurred in *Withania*, another genus with ICS, and this has mainly produced withanolides^15,76^. Whether this diversification is directly related to the significant expansion of SQEs remains elusive. Our findings provide a unique example of the natural genetic engineering of *SQE* genes to give rise to a “high-steroid and steroid derivative” model plant, underscoring the value of our gene toolbox for producing high-value steroidal compounds via synthetic biology. The results also provide a reference genome and a new model for investigating the mysterious process of steroid compound biosynthesis.

## Conclusions

We generated the first, high-quality chromosome-level reference genome for *P. floridana*, a representative species in the genus *Physalis* of Solanaceae. Based on the novel genomic data, we pinpointed another key genetic mutation involved in the loss of a *SEP*-like MADS-box gene, in addition to the previously known MPF2 and MPF3 genes^5,14^, which apparently contributed to the origin of the morphological novelty of ICS, or Chinese lantern. We thus complemented the working model and proposed a new evolutionary scenario in which one mutation compensated for the disadvantageous effect of another mutation during the origin of the Chinese lantern. Morphological innovations such as ICS could be hitchhiking byproducts that significantly improve plant fitness. We also found that the origination and significant expansion of the *SQE* genes might be responsible for the high accumulation of certain steroid compounds in *Physalis* fruits. The CNV of this gene family was correlated with the natural variation of these steroids in the fruits of various solanaceous species. We propose that both the complex fruit-related traits of the Chinese lantern and richness in physalins and derivatives are results of adaptive evolution. Although the molecular mechanisms underlying these novel fruit traits resulting from these mutations and the recruited genes need further investigation by using state-of-the-art technologies, including the CRISPR/Cas9 system, the present work highlights the significant roles of both gene gains and gene losses in the evolution and development of novel fruit traits. In addition to advances in functional genomics in *Physalis*^5,14,43,77–80^, the assembled *Physalis* genome serves as a resource for studies of the 5Gs (genome, germplasm, genes, genomic breeding, and gene editing) in *Physalis* crops, and the data provide a platform for studying plant evolutionary genetics as well as for the genetic improvement and breeding of other solanaceous crops.

## Materials and methods

### Plant materials

Diploid, self-compatible *Physalis pubescens* (syn. *P. floridana* P106)^5^ was mainly used for de novo genome sequencing. Other species from Physaleae^10^ and other plant species were also used for Southern gel-blotting analysis. Before genome sequencing, the karyotype was characterized, and genome size was evaluated using flow cytometric analysis. Genomic DNA from young leaves of a single *P. floridana* plant was extracted using the QIAamp DNA Mini Kit (QIAGEN, Dusseldorf, Germany) following the manufacturer’s instructions.

### DNA library preparation and PacBio sequencing

Eight microgram samples of genomic DNA were sheared using g-Tubes (Covaris, Woburn, MA, USA) and concentrated with AMPure PB magnetic beads. Each SMRT bell library was constructed using the Pacific Biosciences SMRTbell template prep kit (Pacific Biosciences, Menlo Park, USA). Sequencing was carried out on the Pacific Bioscience Sequel platform by Annoroad Gene Technology Co., Ltd. (Beijing, China).

### Genome assembly and quality assessment

To obtain a high-accuracy genome for *P. floridana*, we adopted nearly 100× NGS data and more than 90× PacBio long reads for genome assembly. Falcon^81^ (falcon-kit = 1.0, https://github.com/PacificBiosciences/FALCON/) was employed to assemble the genome with the parameters [seed_coverage=30, length_cutoff_pr=5000]. To improve the local base accuracy of the contigs, Pilon (v1.22)^82^ with the default parameters was employed to polish contigs using the Illumina reads. After assembly, three approaches were employed to evaluate the quality of the genome.

### High-through chromosome conformation capture (Hi-C)

The cells of *P. floridana* were retrieved for Hi-C library preparation according to standard procedures. Biotinylated DNA fragments were enriched and sheared to construct a sequencing library. Sequencing was performed on an Illumina HiSeqX-Ten platform with PE150 bp reads. Hi-C data were used to assign the contigs to chromosomes and then to order and orient the contigs within each chromosome. The Hi-C read contact frequency matrix was visualized using Juicebox (version 1.8.8).

### Gene family classification

To perform the gene family analysis, the proteins of the selected species were downloaded from the NCBI database. All-versus-all BLASTP (version 2.2.26) was performed with an *E*-value cutoff of 1e-5 for all proteins. We then used OrthoMCL (Version 2.09)^83^ to cluster the gene families with an MCL inflation parameter of 1.5.

### Phylogenetic analyses of plant species

We constructed a phylogenetic tree for *P. floridana* and other selected plants based on single-copy orthologous genes. Multiple sequence alignment was performed with MUSCLE (http://www.drive5.com/muscle/)^84^. Fourfold degenerate sites were extracted from sequences of each single-copy gene family and concatenated into a supergene for each species. PhyML 3.0^85^ was used to construct the phylogenetic tree using fourfold degenerate sites via the maximum likelihood method under the GTR model. MCMCTREE in the PAML package^86^ (http://abacus.gene.ucl.ac.uk/software/paml.html) was used to estimate divergence times via the BRMC method^87^ using the soft fossil calibration obtained from the TimeTree website (http://www.timetree.org/).

### Gene family expansion and contraction analysis

Gene families were further filtered out if one species had more than 200 genes or fewer than three genes. The remaining gene families were used to run CAFÉ (version 4.1)^88^ (http://sourceforge.net/projects/cafehahnlab/) with the parameters “-p 0.05 -t 1 -r 10000.” We employed probabilistic graphical models (PGM) to estimate the size of each gene family at each ancestral node of the phylogenetic tree topology using the orthologous genes inferred from OrthoMCL and to obtain a family-wide *p*-value (*p* ≤ 0.05; based on a Monte Carlo resampling procedure) to indicate whether there was significant expansion or contraction in each gene family across species.

### Whole-genome duplication analysis and genome synteny analyses

A syntenic block was identified by using MCScanX software^89^. Searches for putative paralogous and orthologous genes were performed for *P. floridana* and the other species both against themselves and between species by using BLASTP (*E*-value ≤ 1e-5), and we performed multiple sequence alignment by using MUSCLE^84^. The synonymous substitution rate (Ks) was calculated using PALM (version 4.9e) with the YN00 model. We plotted the Ks distribution of all gene pairs in syntenic blocks using in-house Perl scripts. Syntenic gene pairs between *P. floridana* and other species were identified using MCScan software implemented in Python (JCVI v0.84) (https://github.com/tanghaibao/jcvi/wiki/MCscan-Python-version).

### Copy-number variation (CNV) survey

To reveal the CNV of MADS-box genes, the complete sequences of *Arabidopsis* MADS-box genes were collected^90^, and protein sequences of other solanaceous species were extracted and aligned against *Arabidopsis* MADS-box genes using BLASTP (v2.2.28+, parameters: 1e-5, -m = 6). To assess the CNV of genes involved in steroid-related compound synthesis, all genes of interest were annotated using hmmer (v3.1b1) with the Pfam database (Pfam-A.hmm, parameter: -noali), and the Pfam id of each gene (*E*-value ≤ 1e-5) in the Physagulin R pathway in all examined solanaceous species was then extracted for statistical analysis.

### Gel-blotting analyses

Total RNA was isolated by using a total RNA reagent kit (Biomol, Hamburg, Germany). The procedures for DNA and RNA gel blots, probe preparation, hybridization, and signal quantification followed previously described methods^91^. The filters were exposed to a Storage Phosphor Screen (Molecular Dynamics), and signals were quantified with a Typhoon 8600 PhosphorImager (Amersham Pharmacia).

### Characterization of SEP-like and SQE genes

Protein–protein interactions were assessed using yeast two-hybrid assays and bimolecular fluorescence complementation (BiFC) assays. VIGS was performed to reveal the developmental roles of the selected genes. Full-length *StMBP21* cDNA was overexpressed in *Physalis* via *Agrobacterium*-mediated transformation to obtain transgenic *P. floridana*. Phylogenetic analysis was performed via the neighbor-joining method in MEGA^92,93^. The cDNA of the reported genes was isolated using PCR amplification, which was carried out using the KOD-Plus-Neo kit (TOYOBO, Japan). All resulting constructs were sequenced by TSINGKE Biological Technology (Beijing, China), and the primers used in this work (Supplementary Table 38) were also synthesized by TSINGKE Biological Technology (Beijing, China).

### Phenotypic quantification and statistical analysis

Multiple traits, including the flower radius, berry weight (without ICS), and 100-seed weight, were quantified in the VIGS and transgenic plants. Fruits from the indicated plants were harvested for untargeted metabolome analyses. Without special note, statistical analysis was performed by using IBM SPSS Statistics for Windows, Version 24.0 (IBM Corp, NY, USA).

The full description of the “Materials and methods” is available in the Supplementary Methods.

## Supplementary information

Supplementary Tables

Supplementary Methods

Supplementary Figures

## Data Availability

All data are available in the manuscript or the supplementary materials. The *P. floridana* P106 genome sequence data and annotation reported in the article have been deposited in the Genome Warehouse at the National Genomics Data Center (https://ngdc.cncb.ac.cn/) under accession number GWHANUX00000000. The raw sequencing data and RNA-seq data have been deposited in the NCBI at the Sequence Read Archive (SRA) database under accession numbers PRJNA627991 and PRJNA552437.
