# Supplementary material for: The Physalis floridana genome provides insights into the biochemical and morphological evolution of Physalis fruits
Source: Hortic Res. 2021 Nov 18;8:244. doi: 10.1038/s41438-021-00705-w (PMC8602270; doi:10.1038/s41438-021-00705-w)
Supplement: Supplementary file 2 — Supplementary Methods [file 41438_2021_705_MOESM2_ESM.docx]

**Supplementary Methods**

**Plant materials**

The diploid and self-compatible *Physalis pubesecens* (syn. *P. floridana* P106)^1^ and *Solanum lycopersicum var* Heinz 1706 (gifted by Dr. Jing Liu of the Institute of Botany, Chinese Academy of Sciences (IBCAS), Beijing, China), *S. pimpinellifolium* *var* LA1589 (seed bank of Chaoying He’s group, IBCAS), and *C. annuum* L. cv ‘Zunla-1’ (gifted by Dr. Cheng Qin of Zunyi Academy of Agricultural Sciences, Zunyi, China) were cultivated in a greenhouse under long-day conditions (temperature 25–28°C, illumination 16 h). The roots, leaves, and stems from four-week-old seedlings, four floral whorls from flower buds (2, 5, and 7 days before fertilization), blooming flowers (0 days), and calyces and berries from different developing stage fruits (5, 10, 15, 20, and 30 days after fertilization) were harvested from *P. floridana*, *C. annuum* L. cv ‘Zunla-1,’ and *S. pimpinellifolium*. These biological materials were immediately frozen in liquid nitrogen and stored at -80°C for RT-PCR analyses. The tissues of *P. floridana* were also harvested for RNA-seq analyses. The *P. floridana* seeds were germinated on 1/2MS Medium for about four weeks, and seedlings were harvested for Hi-C sequencing. *P. floridana* seeds were germinated in water for about five days, and roots were harvested for karyotype analysis. P106 plants used in genome sequencing were strictly selfed offspring of one plant (at least eight generations). The other species from Physaleae^2^ were used for gel blotting analyses. The fruits of *C. annuum* cultivars (Rookie), *S. lycopersicum* cultivars (Gold Coins), *S. melongena* cultivar (Zixiu), and *S. tuberosum* cultivar (Xingjia 2) collected from Jinhua Academy of Agricultural Sciences (Jinhua, China) were subjected to steroid content analyses by Metabolome Ingenuity Technology Bio-Company (Shanghai, China).

**Karyotype investigation**

Primary root tips (1–2 cm) were taken and put into 0.002 mol/L 8-hydroxyquinoline and pretreated at room temperature for three hours. Carnot stationary solution (anhydrous alcohol:glacial acetic acid = 3:1) fixed the material for about 24 h. The material was washed three times in 0.075 mol/L KCl solution for three minutes each time, then transferred to 75% ethanol solution and stored at 4°C. The materials were placed into 1 mol/L HCl solution with constant temperature (60°C) and allowed to dissociate for five minutes. The dissociated material was soaked in distilled water five times, three minutes at a time, placed into 0.075 mol/L KCl solution, and then kept at room temperature for 30 min. The materials were placed on the slide and dyed with carbol fuchsin for 30 min. Tablet compression and microscopic examination were done with a LEICA DM6 B. Karyotype parameter analysis followed the previously described methods^3^. Centromere index = short arm length/chromosome full length. Arm ratio = long arm length/short arm length. Chromosome numbers were statistically analyzed, and the average was calculated from 20 cells.

**Flow cytometric analysis**

Nuclear suspensions of *P. floridana* were prepared according to a previous report^4^. Approximately 50 mg of fresh leaf tissue was finely chopped with a razor blade in ice cold LB01 lysis buffer (15 mM Tris aminomethane, 2 mM Na_2_EDTA, 0.5 mM spermine.4HCl, 80 mM KCl, 20 mM NaCl, 15 mM mercaptoethanol, and 0.1% (v/v) Triton X-100, pH 8.0)^5^. After chopping, the suspension was filtered through a 50-mm nylon mesh, and RNase A and propidium iodide (50 mg/mL final concentrations) were added. The suspension of isolated nuclei was then incubated on ice in darkness for 20 min prior to analysis. Before starting the analysis, the instrument was checked for linearity using fluorescent check beads (Becton, Dickinson and Company, New Jersey, USA). Approximately 10,000 cells per sample were collected and analyzed on a BD LSR Fortessa flow cytometer (Becton, Dickinson and Company, New Jersey, USA). Sample data were acquired using BD FACS Diva V7.0. Experimental data were analyzed by using Flow Cytometry DNA modeling software ModFitLTV4.0. The fresh leaves of *S. lycopersicum var* Heinz 1706 were treated in parallel in the same way, and its genome size (900 Mb) was used as a reference. Three independent tests were done, and the average value was presented.

**DNA extraction and genome survey**

Genomic DNA from young leaves of a *P. floridana* plant was extracted using the QIAamp DNA Mini Kit (QIAGEN, Dusseldorf, Germany) following the manufacturer’s instructions. The integrity of the DNA was determined with an Agilent 4200 Bioanalyzer (Agilent Technologies, Palo Alto, California). An Illumina PE library (150 bp) was then constructed and sequenced on an Illumina HiSeqX-Ten platform, and the whole genome shotgun sequences were generated. High-quality reads were extracted using Trimmomatic (version 0.36) with the following parameters: (i) containing >2% ambiguous “N” bases; (ii) >20% low-quality bases (quality value less than 20); (iii) containing >30% of adapter sequence; (iv) removal of duplications and indexes; and (v) reads below 75 bases long. Finally, we obtained 149 Gb of clean data in total. K-mer analysis was used to estimate the genome size, repeats, and heterozygosity content. We used the software JELLYFISH (v2.2.0)^6^ to generate a K-mer frequency distribution with a *k*-mer size of 21. The *P. floridana* genome size was calculated via the following formula: total number of k-mer / position of peak depth.

**DNA library preparation and PacBio sequencing**

Eight micrograms of genomic DNA were sheared using g-Tubes (Covaris, Woburn, MA, USA) and concentrated with AMPure PB magnetic beads. Each SMRT bell library was constructed using the Pacific Biosciences SMRTbell template prep kit (Pacific Biosciences, Menlo Park, USA). The constructed libraries were size-selected on a BluePippin™ system for molecules close to 20 kb, followed by primer annealing and the binding of SMRT bell templates to polymerases with a DNA/Polymerase Binding Kit. Sequencing was carried out on the Pacific Bioscience Sequel platform by Annoroad Gene Technology Co. Ltd (Beijing, China). A total of 26 SMRT cells of data were obtained.

**RNA library construction and sequencing analysis**

Tissues were collected from *P. floridana* to perform RNA-seq analysis, and total RNA was isolated using TRIzol Reagent (Invitrogen, Carlsbad, California, USA). Libraries were generated using a NEBNext® Ultra™ RNA Library Prep Kit for Illumina® (NEB, USA) following the manufacturer’s recommendations. Four micrograms (μg) of total RNA with RNA integrity number (RIN) > 7.5 was used as input material for library construction. The RNA-seq library was constructed using the Illumina HiSeq^TM^ 2000 platform at the Beijing Genomics Institute (Beijing, China)^7^. The Iso-Seq libraries were constructed using the Clontech SMARTer PCR cDNA Synthesis Kit (Clontech Laboratories Inc., Palo Alto, CA, USA) according to the manufacturer’s protocol and sequenced on the Pacific Bioscience Sequel platform by Annoroad Gene Technology Co. Ltd (Beijing, China).

**High-through chromosome conformation capture (Hi-C) library sequencing**

The cells of *P. floridana* were retrieved for Hi-C library preparation according to standard procedures including cellular cross-linking, chromatin digestion, labelling of DNA ends, and DNA ligation, purification, and fragmentation. First, nuclear DNA of *P. floridana* was cross-linked *in situ*, extracted, and then digested by the restriction enzyme *Mbo* I. The sticky ends of the digested fragments were biotinylated, diluted, and randomly ligated. Biotinylated DNA fragments were enriched and sheared to construct a sequencing library. Finally, sequencing was performed on an Illumina HiseqX-Ten platform with PE150 bp reads.

**Genome assembly and quality assessment**

To obtain a high-accuracy genome for *P. floridana*, we adopted nearly 100× data of NGS and above 90× PacBio long reads for genome assembly and performed two main steps. Falcon^8^ (falcon-kit=1.0, <https://github.com/PacificBiosciences/FALCON/>) was employed to assemble the genome with the parameters [seed_coverage=30, length_cutoff_pr=5000]. First, all raw subreads were overlapped with seed reads, and we then selected 30× longest subreads from all raw subreads for error correction. After correction, pre-assembly was performed. A graph was constructed according to the overlaps between pre-assembly reads. The genome sequence was then constructed. To improve the local base accuracy of the contigs, Pilon (v1.22)^9^ with default parameters was used to polish contigs using the Illumina reads.

After assembly, three approaches were employed to evaluate the quality of the genome. First, Benchmarking Universal Single-Copy Orthologs (BUSCO v3.0)^10^ was applied as an evolutionary measure of genome completeness using the embryophyta_odb10 (1,375 genes) as a query. Second, the Illumina short reads were re-mapped to the genome using the Burrows-Wheeler Aligner (BWA) software (v0.7.9a) for calculating the mapping rates and genome coverage. Finally, the transcripts produced by Illumina RNA-seq and PacBio iso-seq were used to estimate the quality of the assembled genome.

**Chromosome-level genome construction by Hi-C**

Hi-C data were used to assign the contigs to chromosomes and then to order and orient the contigs within each chromosome. Hi-C clean reads were mapped to these contigs, and Hi-C contact frequency between genomic loci was computed using Juicer (<https://github.com/aidenlab/juicer>) (version 1.7.6)^11^. The program 3D-DNA (<https://github.com/theaidenlab/3d-dna>) (version 180114)^12^ was used to anchor and orient contigs based on contact frequency calculated from mapped Hi-C read pairs to obtain the pseudomolecules for two rounds using default parameters. During this process, we manually corrected misassembled ordered and oriented contigs of DNA based on Hi-C data and took advantage of the telomere-to-telomere contact enrichment associated with genomes in Rabl configuration to obtain 12 pseudo-chromosomes using Juicebox Assembly Tools (<https://github.com/aidenlab/Juicebox/wiki/Juicebox-Assembly-Tools>) (JBAT version 1.8.8). The Hi-C reads contact frequency matrix was visualized using Juicebox (version 1.8.8). As expected, the spatial proximity, as reflected by the Hi-C interaction intensity, decreased along with the increasing physical distance between two loci.

**Identifying repeat sequences**

Repetitive sequences are an important part of the genome. The two main categories are tandem repeats and interspersed repeats. We employed two strategies for repeat prediction using homology-based and *de novo* methods. For homology-based repeat identification, we used RepeatMasker and RepeatProteinMask (version 4.0.5)^13^ to screen the whole *P. floridana* genome for known transposable elements in the RepBase database (<https://www.girinst.org/server/RepBase/index.php>)^14^. The *de novo* transposable elements were identified by using RepeatModeler (version 1.0.11) (<http://www.repeatmasker.org/RepeatModeler/>). Tandem Repeats Finder (TRF, version 4.09) with default parameters was used to search for tandem repeats in the genome^15^. The predicted LTRs were further classified into intact and non-intact LTRs, and the insertion time was estimated as T = K/2μ (K is the divergence rate, and μ is the neutral mutation rate; we used 1.38 × 10^−8^ in LTR_retriever) using the scripts implemented in the LTR_retriever package^16^. Repeat sequences were masked to reduce the complication in genome annotation^17^.

**Gene structure prediction**

We combined three strategies for gene structure prediction by integrating evidence from transcriptome sequencing, homolog searching, and *de novo* predictions. The RNA-seq reads from multiple tissues were assembled by Trinity (version 2.4.0) with parameters (--min_kmer_cov 4 --group_pairs_distance 500 --full_cleanup --no_version_check). High-quality full-length transcripts were collected from Iso-Seq raw reads using SMRT Link (version 7.0) software. The transcriptomes, including transcripts assembled from Illumina RNA_seq and high-quality full-length PacBio long reads, were aligned against the *P. floridana* genome using Program to Assemble Spliced Alignment (PASA, <http://pasa.sourceforge.net/>)^18^. For homologous comparisons, the reference protein sequences for five species were downloaded from the NCBI database (*Capsicum annuum*, *Solanum lycopersicum*, *Solanum pennellii*, *Solanum tuberosum*, and *Nicotiana tabacum*) and were aligned against the *P. floridana* genome using TBLASTN (<http://blast.ncbi.nlm.nih.gov/Blast.cgi>)^19^ with the parameters of “-seg yes -evalue 1e-5.” All blast hits results were merged and concatenated; we then filtered the low-quality records. Genewise (<http://www.ebi.ac.uk/~birney/wise2/>)^20^ with default parameters was used to predict the gene structure. For *de novo* prediction, we employed three software programs, Augustus (<http://augustus.gobics.de/>)^21^, SNAP (<https://github.com/KorfLab/SNAP>)^22^, and GeneMark-ET (<http://exon.gatech.edu/GeneMark/>)^23^. To obtain high accuracy, we selected the high-quality proteins collected from PASA gene models to train the best gene characteristic parameters. Finally, according to the three results, EvidenceModeler (EVM)^24^ (<http://evidencemodeler.github.io/>) was used to generate the non-redundant reference gene set.

**Gene functional assignments**

The functional annotation of the predicted proteins was mainly done according to searches against various functional databases, including NT (<https://www.ncbi.nlm.nih.gov/nucleotide/>), Swissprot (<https://web.expasy.org/docs/swiss-prot_guideline.html>), PFAM (<http://xfam.org/>)^25^, NR (<ftp://ftp.ncbi.nlm.nih.gov/blast/db/FASTA/nr.gz>), eggNOG (<http://eggnogdb.embl.de/>)^26^, GO (<http://geneontology.org/page/go-database>)^27^, and KEGG (<http://www.genome.jp/kegg/>)^28^.

**Noncoding RNA prediction**

Three types of ncRNAs, microRNA (miRNA), ribosomal RNA (rRNA), and small nuclear RNA (snRNA), were identified in the assembled *P. floridana* genome using the cmsearch program of INFERNAL (version 1.0.2) against the Rfam database (version 14.1)^29^. The tRNA genes were identified using tRNAscan-SE (version 1.3.1)^30^ with eukaryote parameters.

**Gene family classification**

To perform the gene family analysis, proteins of 12 other species, *C. annuum* (CM334), *S. lycopersicum* (SL3.0), *S. pennellii* (SPENNV200), *S. tuberosum* (SolTub_3.0), *Petunia axillaris*, *Nicotiana attenuata*, *A. thaliana* (TAIR10.1), *V. vinifera* (PN40024), *G. max* (Wm82.a2.v1), *P. trichocarpa* (Ptr v3.0), *A. coerulea* (Goldsmith v3.1), *O. sativa* (IRGSP-1.0), *Z. mays* (RefGen_v4), and *A. trichopoda* (AMTR1.0), were download from the NCBI database, and these of *Lycium barbarum* were requested from the authors of the related article^31^. For genes with alternatively spliced variants, only the longest transcript was selected. Genes encoding proteins of fewer than 30 amino acids were discarded. All-versus-all blastp (version 2.2.26) was performed with an E-value cutoff of 1e-5 for all proteins. We then used OrthoMCL (Version 2.09)^32^ to cluster the gene family with the help of an MCL inflation parameter of 1.5. Except for the orthologs, the remaining gene families in one genome were generally defined as the specific gene families in this genome compared to another genome and *vice versa*.

**Phylogenetic analyses of plant species**

We constructed a phylogenetic tree for *P. floridana* and other selected plants (*C. annuum*, *S. lycopersicum*, *S. pennellii*, *S. tuberosum*, *A. thaliana*, *V. vinifera*, *G. max*, *P. trichocarpa*, *A. coerulea*, *O. sativa*, *Z. mays*, and *A. trichopoda*) based on single-copy orthologous genes. Multiple sequence alignment was performed with MUSCLE (<http://www.drive5.com/muscle/>)^33^. Fourfold degenerate sites were extracted from sequences of each single-copy gene family and concatenated into a supergene for each species. PhyML 3.0^34^ was used to construct the phylogenetic tree using fourfold degenerate sites via the maximum likelihood method under the GTR model.

**Species divergence time estimates**

The phylogenetic tree constructed according to the coding sequences of single-copy orthologous genes was used to estimate divergence times. MCMCTREE in the package of PAML^35^ (<http://abacus.gene.ucl.ac.uk/software/paml.html>) was used to estimate divergence time via the BRMC method^36^ using soft fossil calibration collected from the TimeTree website (<http://www.timetree.org/>). The soft fossils of *O. sativa* / *A. thaliana*^37^, *G. max* / *A. thaliana*^38^, *S. lycopersicum* / *C. annuum*^39^, *O. sativa* / *Z. mays*^40^, and *V. vinifera* / *S. tuberosum*^41^ were used for correction.

**Positive selection analysis**

We employed PAML (version 4.9h)^42^ to detect genes under positive selection for each single copy gene family in the Solanaceae. Proteins from each single copy gene family were used to perform multiple sequence alignments (MSAs) using MUSCLE (<http://www.drive5.com/muscle/>)^33^ with the default parameters. We back-translated the protein alignment to coding sequences. Positive selection for each SCG of the Solanaceae was estimated using the branch-site model^42^. The genus *Physalis* was selected to serve the foreground position, and the others in the phylogeny as backgrounds. *P*-values were calculated using the chi2 program to adjust for the false discovery rate.

**Gene family expansion and contraction analysis**

Gene families were further filtered out if one species had more than 200 genes or fewer than three genes. The remaining gene families were used to run CAFÉ (version 4.1)^43^ (<http://sourceforge.net/projects/cafehahnlab/>) with parameters “-p 0.05 -t 1 -r 10000.” We employed the PGM (probabilistic graphical models) to estimate the size of each gene family at each ancestral node of the phylogenetic tree topology using the orthologous genes inferred from OrthoMCL and to obtain a family-wide *P*-value (*P* ≤ 0.05; based on a Monte Carlo resampling procedure) to indicate whether there was a significant expansion or contraction in each gene family across species.

**Whole-genome duplication analysis**

A syntenic block was identified by using MCScanX software^44^. *P. floridana* and other species were searched against themselves and between species by blastp (E value ≤ 1e-5) for putative paralogous and orthologous genes, and we performed multiple sequences alignment by using MUSCLE^33^. The synonymous substitution rate (Ks) was calculated using PALM (version 4.9e) with the YN00 model. We plotted the Ks distribution of all gene pairs in syntenic blocks using in-house Perl scripts.

**Genome synteny analyses**

Syntenic gene pairs between *P. floridana* and other species were identified using the MCScan software implemented in Python (JCVI v0.84) (<https://github.com/tanghaibao/jcvi/wiki/MCscan-Python-version>). Syntenic blocks between two species were first found using ‘jcvi.compara.catalog ortholog’ implemented in JCVI with parameters “ --dbtype=nucl --cscore=0.7 --dist=20.” The ‘.anchors’ and ‘.bed’ files were selected for subsequent analysis using default parameters. The syntenic regions of the target species and the comparison species were shown graphically. The *P. floridana* -specific regions were defined when at least ten consecutive genes of *P. floridana* had no syntenic hits in *S. lycopersicum* and *C. annuum*. Similarly, the number of *S. lycopersicum* -specific regions was calculated when at least ten consecutive genes of *S. lycopersicum* had no syntenic hits in *P. floridana* or *C. annuum*.

**Copy number variation (CNV) survey of the genes of interest**

To reveal the CNV of MADS-box genes, the complete sequences of *Arabidopsis* MADS-box genes were collected^45^, and protein sequences of other solanaceous species were extracted and aligned against *Arabidopsis* MADS-box genes using BLASTP (v2.2.28+, parameters: 1e-5, -m=6). For the CNV of genes involved in steroid-related compounds synthesis, all genes of interest were annotated using hmmer (v3.1b1) with the Pfam database (Pfam-A.hmm, parameter: --noali), and then the Pfam id of each gene (E value ≤ 1e-5) in the Physagulin R pathway in all examined solanaceous species was extracted for statistical analysis.

**Isolation and molecular identification of *SEP*-like MADS-box genes**

The sequences of four *SEP*-like MADS-box genes (*AtSEP1*, *AtSEP2*, *AtSEP3*, and *AtSEP4*) in *A. thaliana* were obtained from TAIR (<https://www.arabidopsis.org/>)^46,47^. The sequences of *SEP*-like MADS-box genes in *S. lycopersicum* were also collected^48,49^. Based on the sequence information, BLAST was performed on the Sol Genomics Network (<https://solgenomics.net/>) to search for homologous genes in *S. pimpinellifolium*, *C. annuum* L. cv ‘Zunla-1,’ and *S. tuberosum*. The homologous gene sequences in *P. floridana* were screened out from the transcriptome and genomic databases.

**qRT-PCR analyses**

For real-time reverse transcription polymerase chain reaction (qRT-PCR), total RNA was isolated using a Plant RNeasy Mini kit (QIAGEN, Dusseldorf, Germany). The qRT-PCR was performed by using the SYBR *Premix Ex Taq* (Perfect Real Time) Kit (TaKaRa, Dalian, China) as follows: 1.0 μL of a 1/10 dilution of cDNA in ddH_2_O was added to 12.5 μL of 2 × SYBR *Premix Ex Taq* (Takara, Dalian, China), 0.5 μL of 10 μM of each primer (Supplementary Table 38), and 0.5 μL ROX Reference Dye II; ddH_2_O was then added to a final volume of 25.0 μL. Based on the Mx3000P Real Time PCR System (Agilent Stratagene, La Jolla, CA, USA), the PCR amplification procedure was 30 s at 95°C for 1 cycle followed by 46 cycles of 5 s at 95°C, 45 s at 60°C, then 1 min at 95°C for 1 cycle, 30 s at 60°C for 1 cycle, and 30 s at 95°C for 1 cycle. *ACTIN*, *Ubiquitin,* or *EF1a* genes, the housekeeping genes, were used as the internal controls. The relative expression quantification was calculated according to the 2^-ΔΔ^*^C^*^T^ method^50^. The heatmap of gene expression was constructed using TBtools^51^.

**Gel blotting analyses**

Total RNA was isolated by using a total RNA reagent kit (Biomol, Hamburg, Germany). DNA and RNA gel blots, preparation of probes, hybridization, and signal quantification followed previously described methods^52^. The filters were exposed to a Storage Phosphor Screen (Molecular Dynamics), and signals were quantified with Typhoon 8600 PhosphorImager (Amersham Pharmacia).

**Subcellular localization analyses**

The ORFs of the *SEP*-like genes were cloned into a Super1300 expression vector that contained the *GFP* gene via designed gene-specific primers (Supplementary Table 38). The recombinant construct was injected into leaf epidermal cells of *N. benthamiana* and *P. floridana* via *Agrobacterium tumefaciens*. The GFP fluorescence signal was detected using confocal laser scanning microscopy (Olympus FV1000 MPE).

**Protein-protein interaction analyses**

The yeast two-hybrid assays followed the previously described method^53^ with minor modifications. The open reading frames (ORFs) of *SEP*-like MADS-box genes in *S. pimpinellifolium*, *C. annuum* L. cv ‘Zunla-1,’ and *P. floridana* were constructed into pGBKT7 (Clontech, Mountain View, CA, USA) via the designed gene-specific primers (Supplementary Table 38). Autoactivation and toxicity of BD-vectors were checked in yeast strain Y2H Gold with SD/-His-Trp and SD/-Trp media, respectively. No autoactivation or toxicity was found. The ORF of *MPF2* and *MPF3* was constructed into pGADT7 (Clontech, Mountain View, CA, USA). The plasmids of bait (SEP-like MADS-box genes-pGBKT7) and prey (MPF2-pGADT7 or MPF3-pGADT7) were co-transformed into Y2H Gold. The transformation mix was plated on selective media (SD/-Ade-His-Leu-Trp and SD/-Ade-His-Leu-Trp-X-α-Gal) and then cultured at 30°C for 3–5 days. Growth on SD/-Ade-His-Leu-Trp medium or blue coloration on SD/-Ade-His-Leu-Trp-X-α-Gal indicated the interaction between the bait and the prey.

For bimolecular fluorescence complementation (BiFC) assays, the ORFs of the *SEP*-like genes and *MPF2/MPF3* genes were respectively cloned into pSPYNE-35S and pSPYCE-35S^54^ with *Spe*I and *Sal*I (TaKaRa, Dalian, China) cutting sites through the In-Fusion® HD Cloning Kit (Clontech, Mountain View, CA, USA). The two vectors were designed to express either N- or C-terminal halves of a YFP. The construct combination of two proteins fused with the N- or C-terminal halves of YFP was injected into leaf epidermal cells of *Nicotiana benthamiana* via *Agrobacterium tumefaciens*. Forty-eight hours after injection, the YFP fluorescence signal was detected using confocal laser scanning microscopy (Olympus FV1000 MPE).

**VIGS analyses in *P. floridana***

For VIGS of *SEP*-like genes, the gene-specific C-terminal of each MADS-box genes in *P. floridana* was designed as a probe. The probe regions of *PfSEP3-1-TRV2* and *PfSEP3-2-TRV2* had high sequence identity. Therefore, *PfSEP3-1-VIGS* and *PfSEP3-2-VIGS* plants were named *PfSEP3-1/2-VIGS* plants, in which both paralogous genes were down-regulated. The *Agrobacterium* solution transformed recombined vectors (*PfSEP3-1-TRV2*, *PfSEP3-2-TRV2*, *PfM29-TRV2*, *PfMADS1-TRV2*, *PfCMB1-TRV2*, and *PfRIN-TRV2*) were mixed equally with the *Agrobacterium* solution transformed TRV1. The procedure followed the previous description^55^. At least four seedlings were employed in each gene-specific VIGS analysis.

For VIGS of *PfSQE* genes, 15 *PfSQE* genes were clustered into three groups, and group-specific probes were designed. The *Agrobacterium* solution transformed with one vector of *PfSQE-1-TRV2*, *PfSQE-2-TRV2*, and *PfSQE-3-TRV2* was mixed equally with the *Agrobacterium* solution transformed with *TRV1*. The mixture was first used to inject tobacco seedlings; after two weeks, the leaf tissue liquid from these tobacco plants was injected into six-day-old berries of *P. floridana*.

**Generation of overexpressing *StMBP21* transgenic *Physalis* plants**

Full-length *StMBP21* cDNAs were cloned into the plant binary vector pBin19, introduced into the LBA4404 strain of *Agrobacterium tumefaciens,* and transformed into *P. floridana*. The transgenic plant lines were selected on 50 mg/L kanamycin and identified via floral phenotypic variation.

**Phenotypic quantification**

Plant height was defined as the length from the stem bottom to the plant top (n = 20). The length/width ratio represented leaf size. The flower radius, berry weight (without the ICS), and 100-seed weight were measured, and berry volume (without the ICS) was measured by immersing the whole berry in water in a volumetric cylinder. In addition, the Chinese lantern length, fruit stalk length, number of seeds per fruit, and 1000-seed weight were measured in the *SEP*-like VIGS analyses. Each trait was evaluated based on 20–40 samples.

**Untargeted metabolome analysis**

The 25-day-old fruits of a *C. annuum* cultivar (Rookie), a *S. lycopersicum* cultivar (Gold Coins), a *S. melongena* cultivar (Zixiu), and a *S. tuberosum* cultivar (Xingjia 2) and the fruits 14 days after VIGS-treatments of the *PfSQE*-VIGS *P. floridana* plants were harvested for untargeted metabolome analyses. Sample preprocessing, liquid chromatography-tandem mass spectrometry (LC-MS/MS) detection, the mobile phase, and the parameters optimized in both positive and negative ion modes for mass spectrometry were performed as previously described^56,57^. Seven technical replicates of each fruit sample were performed for natural variation of steroid-related compounds in solanaceous species, while three VIGS fruits were analyzed for each *PfSQE*-group-specific VIGS. Raw data were converted to a common (mz.data) format by Agilent Masshunter Qualitative Analysis B.08.00 software (Agilent Technologies, USA). In the R software platform, the XCMS program was used for peak identification, retention time correction, and automatic integration pretreatment. The data were subjected to weight normalization. Visualization matrices containing the sample name, m/z-RT pair, and peak area were obtained. After editing, the data matrices were imported into SIMCA-P 13.0 (Umetrics, Umea, Sweden), mean-centered, and scaled to Pareto variance followed by multivariate analysis. The maximum and minimum values were removed for each group of steroid-related species, and thus five replicates were included for evaluating natural variation of the steroid derivatives among solanaceous species.

**cDNA isolation, sequencing analyses and primer synthesis**

Total RNA was isolated using the TRIzol Reagent (Invitrogen, Carlsbad, USA) and digested using the RQ1 RNase-free DNase (Promega, Madison, USA). The first-strand cDNA was synthesized by oligo(dT)17 and M-MLV Reverse Transcriptase (Invitrogen, Carlsbad, CA, USA). The cDNA of the reported genes was isolated using PCR amplifications that were carried out using the KOD-Plus-Neo kit (TOYOBO, Japan). After purification using the High Pure PCR Product Purification Kit (Roche, Mannheim, Germany), PCR products were sequenced by TSINGKE Biological Technology (Beijing, China). Amplified fragments were cloned into *pEASY*-blunt cloning vector (TransGen, Beijing, China) and transformed into the *Trans1*-T1 Phage Resistant Chemically Competent Cell (TransGen, Beijing, China). Plasmids were extracted using the HighPure Rapid Mini Plasmid Kit (Biomed, Beijing, China). All resulting constructs were sequenced by TSINGKE Biological Technology (Beijing, China), and the primers used in this work (Supplementary Table 38) were synthesized by TSINGKE Biological Technology (Beijing, China).

**Phylogenetic analysis of SEP-like and SQE genes**

Phylogenetic analysis was performed by the Neighbor-Joining method in MEGA^58,59^. The tree was constructed using aligned coding sequences with 1000 bootstrap replicates using the optimum Tamura three-parameter model with T92 using a discrete Gamma distribution (+G).

**Statistical analysis**

Without special note, statistical analysis was performed by using IBM SPSS Statistics for Windows, Version 24.0 (IBM Corp, NY, USA).

**References**

1. He, C.Y. & Saedler, H. Heterotopic expression of *MPF2* is the key to the evolution of the Chinese lantern of *Physalis*, a morphological novelty in Solanaceae. *Proc. Natl. Acad. Sci. USA*. **102**, 5779–5784 (2005).

2. Hu, J.Y. & Saedler, H. Evolution of the inflated calyx syndrome in Solanaceae. *Mol. Biol. Evol*. **24**, 2443–2453 (2007).

3. Paszko, B. A critical review and a new proposal of karyotype asymmetry indices. *Plant Syst. Evol*. **258**, 39–48 (2006).

4. Galbraith, D.W. et al. Rapid flow cytometric analysis of the cell cycle in intact plant tissues. *Science*. **220**, 1049–1051 (1983).

5. Dpooležel, J., Binarová, P. & Lcretti, S. Analysis of nuclear DNA content in plant cells by flow cytometry. *Biol. Plant*. **31**, 113–120 (1989).

6. Marcais, G. & Kingsford, C. A fast, lock-free approach for efficient parallel counting of occurrences of k-mers. *Bioinformatics*. **27**, 764–770 (2011).

7. Gao, H.H., Li, J., Wang, L., Zhang, J. S., & He, C.Y. Transcriptomic variation of the flower–fruit transition in *Physalis* and *Solanum*. *Planta*. **252**, 28 (2020).

8. Chin, C.S. et al. Phased diploid genome assembly with single-molecule real-time sequencing. *Nat. Methods*. **13**, 1050–1054 (2016).

9. Walker, B.J. et al. Pilon: an integrated tool for comprehensive microbial variant detection and genome assembly improvement. *PLoS One*. **9**, e112963 (2014).

10. Simao, F.A., Waterhouse, R.M., Ioannidis, P., Kriventseva, E.V. & Zdobnov, E.M. BUSCO: assessing genome assembly and annotation completeness with single-copy orthologs. *Bioinformatics*. **31**, 3210–3212 (2015).

11. Durand, N.C. et al. Juicer provides a one-click system for analyzing loop-resolution Hi-C experiments. *Cell Syst*. **3**, 95–98 (2016).

12. Dudchenko, O. et al. De assembly of the *Aedes aegypti* genome using Hi-C yields chromosome-length scaffolds. *Science*. **356**, 92–95 (2017).

13. Tarailo-Graovac, M. & Chen, N. Using RepeatMasker to identify repetitive elements in genomic sequences. *Curr. Protoc. Bioinformatics*. **25**, 4.10.1– 4.10.14 (2009).

14. Jurka, J. et al. Repbase Update, a database of eukaryotic repetitive elements. *Cytogenet. Genome Res*. **110**, 462–467 (2005).

15. Benson, G. Tandem repeats finder: a program to analyze DNA sequences. *Nucleic Acids Res*. **27**, 573-580 (1999).

16. Ou, S.J. & Jiang, N. LTR_retriever: a highly accurate and sensitive program for identification of long terminal repeat retrotransposons. *Plant Physiol*. **176**, 1410–1422 (2018).

17. Yandell, M. & Ence, D. A beginner's guide to eukaryotic genome annotation. *Nat. Rev. Genet*. **13**, 329–342 (2012).

18. Roberts, A., Pimentel, H., Trapnell, C. & Pachter, L. Identification of novel transcripts in annotated genomes using RNA-Seq. *Bioinformatics*. **27**, 2325–2329 (2011).

19. McGinnis, S. & Madden, T.L. BLAST: at the core of a powerful and diverse set of sequence analysis tools. *Nucleic Acids Res*. **32**, W20–25 (2004).

20. Birney, E., Clamp, M. & Durbin, R. Genewise and genomewise. *Genome Res*. **14**, 988–995 (2004).

21. Stanke, M., Steinkamp, R., Waack, S. & Morgenstern, B. AUGUSTUS: a web server for gene finding in eukaryotes. *Nucleic Acids Res*. **32**, W309–312 (2004).

22. Korf, I. Gene finding in novel genomes. *BMC Bioinformatics*. **5**, 59 (2004).

23. Ter-Hovhannisyan, V., Lomsadze, A., Chernoff, Y.O. & Borodovsky, M. Gene prediction in novel fungal genomes using an *ab initio* algorithm with unsupervised training. *Genome Res*. **18**, 1979–1990 (2008).

24. Haas, B.J. et al. Automated eukaryotic gene structure annotation using EVidenceModeler and the Program to Assemble Spliced Alignments. *Genome Biol*. **9**, R7 (2008).

25. Finn, R.D. et al. Pfam: the protein families database. *Nucleic Acids Res*. **42**, D222–230 (2014).

26. Powell, S. et al. eggNOG v3.0: orthologous groups covering 1133 organisms at 41 different taxonomic ranges. *Nucleic Acids Res*. **40**, D284–289 (2012).

27. Ashburner, M. et al. Gene Ontology: tool for the unification of biology. *Nat. Genet*. **25**, 25–29 (2000).

28. Kanehisa, M., Goto, S., Sato, Y., Furumichi, M. & Tanabe, M. KEGG for integration and interpretation of large-scale molecular data sets. *Nucleic Acids Res*. **40**, D109–114 (2012).

29. Griffiths-Jones, S. et al. Rfam: annotating non-coding RNAs in complete genomes. *Nucleic Acids Res*. **33**, D121–124 (2005).

30. Lowe, T.M. & Eddy, S.R. tRNAscan-SE: a program for improved detection of transfer RNA genes in genomic sequence. *Nucleic Acids Res*. **25**, 955–964 (1997).

31. Cao, Y.L., et al. Wolfberry genomes and the evolution of *Lycium* (Solanaceae).

*Commun Biol.* **4**, 671 (2021).

32. Li, L., Stoeckert, C.J. & Roos, D.S. OrthoMCL: identification of ortholog groups for eukaryotic genomes. *Genome Res*. **13**, 2178–2189 (2003).

33. Edgar, R.C. MUSCLE: multiple sequence alignment with high accuracy and high throughput. *Nucleic Acids Res*. **32**, 1792–1797 (2004).

34. Guindon, S. et al. New algorithms and methods to estimate maximum-likelihood phylogenies: assessing the performance of PhyML 3.0. *Syst. Biol*. **59**, 307–321 (2010).

35. Yang, Z.H. PAML 4: phylogenetic analysis by maximum likelihood. *Mol. Biol. Evol*. **24**, 1586–1591 (2007).

36. Sanderson, M.J. r8s: inferring absolute rates of molecular evolution and divergence times in the absence of a molecular clock. *Bioinformatics*. **19**, 301–302 (2003).

37. Dexter, K. & Chave, J. Evolutionary patterns of range size, abundance and species richness in Amazonian angiosperm trees. *PeerJ*. **4**, e2402 (2016).

38. Ndiribe, C. et al. Plant functional and phylogenetic turnover correlate with climate and land use in the Western Swiss Alps. *J. Plant Ecol*. **7**, 439–450 (2013).

39. Wu, Z.H. et al. A precise chloroplast genome of *Nelumbo nucifera* (Nelumbonaceae) evaluated with Sanger, Illumina MiSeq, and PacBio RS II sequencing platforms: insight into the plastid evolution of basal eudicots. *BMC Plant Biol*. **14**, 289 (2014).

40. Verboom, G.A., Stock, W.D. & Cramer, M.D. Specialization to extremely low-nutrient soils limits the nutritional adaptability of plant lineages. *Am. Nat*. **189**, 684–699 (2017).

41. Harris, L.W. & Davies, T.J. A complete fossil-calibrated phylogeny of seed plant families as a tool for comparative analyses: testing the ‘Time for Speciation’ hypothesis. *PLoS One*. **11**, e0162907 (2016).

42. Zhang, J.Z., Nielsen, R. & Yang, Z.H. Evaluation of an improved branch-site likelihood method for detecting positive selection at the molecular level. *Mol. Biol. Evol*. **22**, 2472–2479 (2005).

43. De Bie, T., Cristianini, N., Demuth, J.P. & Hahn, M.W. CAFE: a computational tool for the study of gene family evolution. *Bioinformatics*. **22**, 1269–1271 (2006).

44. Wang, Y.P. et al. MCScanX: a toolkit for detection and evolutionary analysis of gene synteny and collinearity. *Nucleic Acids Res*. **40**, e49 (2012).

45. Paenicová, L. et al. Molecular and phylogenetic analyses of the complete MADS-box transcription factor family in *Arabidopsis*: new openings to the MADS world. *Plant Cell*. **15**,1538–1551 (2013).

46. Ditta, G., Pinyopich, A., Robles, P., Pelaz, S. & Yanofsky, M.F. The *SEP4* gene of *Arabidopsis thaliana* functions in floral organ and meristem identity. *Curr. Biol*. **14**, 1935–1940 (2004).

47. Pelaz, S., Ditta, G.S., Baumann, E., Wisman, E. & Yanofsky, M.F. B and C floral organ identity functions require *SEPALLATA* MADS-box genes. *Nature*. **405**, 200–203 (2000).

48. Soyk, S. et al. Bypassing negative epistasis on yield in tomato imposed by a domestication gene. *Cell*. **169**, 1142–1155 (2017).

49. Zhang, J.L. et al. A tomato MADS-box protein, SlCMB1, regulates ethylene biosynthesis and carotenoid accumulation during fruit ripening. *Sci. Rep*. **8**, 3413 (2018).

50. Livak, K.J. & Schmittgen, T.D. Analysis of relative gene expression data using real-time quantitative PCR and the 2^−ΔΔC^_T_ Method. *Methods*. **25**, 402–408 (2001).

51. Chen, C.J. et al. TBtools, a integrative toolkit developed for interactive analyses of big biological data. *Mol. Plants*. **13**, 1194–1202 (2020).

52. He, C.Y., Zhang, J.S. & Chen, S.Y. A soybean gene encoding a proline-rich protein is regulated by salicylic acid, an endogenous circadian rhythm and by various stresses. *Theor. Appl. Genet*. **104**, 1125–1131 (2002).

53. He, C.Y., Sommer, H., Grosardt, B., Huijser, P. & Saedler, H. PFMAGO, a MAGO NASHI-like factor, interacts with the MADS-domain protein MPF2 from *Physalis floridana*. *Mol. Biol. Evol*. **24**, 1229–1241 (2007).

54. Walter, M. et al. Visualization of protein interactions in living plant cells using bimolecular fluorescence complementation. *Plant J*. **40**, 428–438 (2004).

55. Zhang, J.S., Zhao, J., Zhang, S.H. & He, C.Y. Efficient gene silencing mediated by tobacco rattle virus in an emerging model plant *Physalis*. *PLoS One*. **9**, e85534 (2014).

56. Zhan, X.R. et al. Comparative metabolomic and proteomic analyses reveal the regulation mechanism underlying MeJA-induced bioactive compound accumulation in cutleaf groundcherry (*Physalis angulata* L.) hairy roots. *J. Agric. Food Chem*. **66**, 6336–6347 (2018).

57. Zhan, X.R. et al. Bioactive compounds induced in *Physalis angulata* L. by methyl-jasmonate: an investigation of compound accumulation patterns and biosynthesis-related candidate genes. *Plant Mol. Biol*. **103**, 341-354 (2020).

58. Saitou, N. & Nei, M. The neighbor-joining method: a new method for reconstructing phylogenetic trees. *Mol. Biol. Evol*. **4**, 406–425 (1987).

59. Kumar, S., Stecher, G. & Tamura, K. MEGA7: molecular evolutionary genetics analysis version 7.0 for bigger datasets. *Mol. Biol. Evol*. **33**, 1870–1874 (2016).
