# Supplementary material for: The Physalis floridana genome provides insights into the biochemical and morphological evolution of Physalis fruits
Source: Hortic Res. 2021 Nov 18;8:244. doi: 10.1038/s41438-021-00705-w (PMC8602270; doi:10.1038/s41438-021-00705-w)
Supplement: Supplementary file 3 — Supplementary Figures [file 41438_2021_705_MOESM3_ESM.pdf]

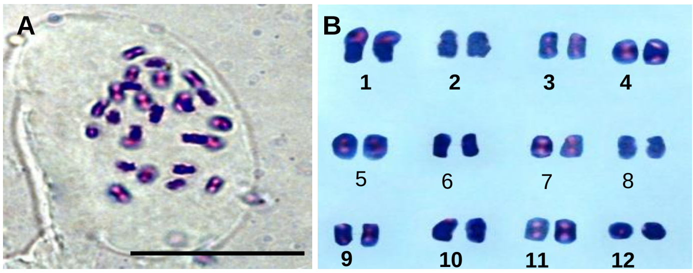

C

Chromosome number	Relative length （%）	Centromere index (%)	Arm ratio	Type
1	1.37+2.13=3.50	39.14	1.55	m
2	1.35+1.56=2.91	46.39	1.16	m
3	1.25+1.64=2.89	43.25	1.31	m
4	1.18+1.44=2.62	45.04	1.22	m
5	1.17+1.34=2.51	46.61	1.15	m
6	1.26+1.26=2.52	50.00	1.00	M
7	1.21+1.26=2.47	48.99	1.04	m
8	0.94+1.52=2.46	38.21	1.62	m
9	1.03+1.33=2.36	43.64	1.29	m
10	1.07+1.20=2.27	47.14	1.12	m
11	1.04+1.14=2.18	47.71	1.10	m
12	0.72+0.85=1.57	45.86	1.18	m

Supplementary Fig. 1 Chromosomal karyotype analysis of *P. floridana* P106. **A** Chromosomes in a root tip cell. Bar = 20 µm. **B** Chromosomal karyotype model. **C** Karyotype statistical parameters of 12 chromosomes in *P. floridana*. m, Metacentric chromosome; M, Median centromere chromosome

A

Statistic	Number
Number of Raw Reads	1,021,770,006
Number of Raw Bases	153,265,500,900
Number of Clean Reads	992,846,510
Rate of Clean Reads (%)	97.17
Number of Clean Bases	148,926,976,500
Number of Low-quality Reads	8,339,810
Rate of Low-quality Reads (%)	0.82
Number of Reads containing over 5% of N	20,132
Rate of Reads containing over 5% of N (%)	0
Number of Adapter Polluted Reads	20,563,554
Rate of Adapter Polluted Reads (%)	2.01
Rate of Raw Q30 Bases (%)	93.55
Rate of Clean Q30 Bases (%)	93.91

B

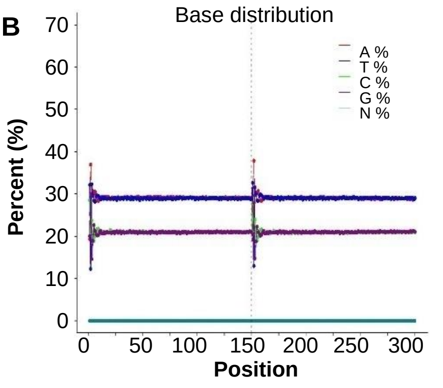

C

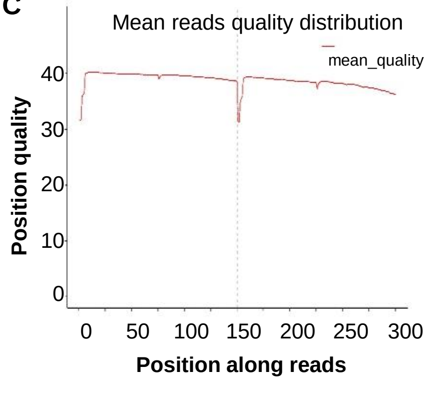

Supplementary Fig. 2 Landscape of Illumina sequencing data. A Filtering statistical results. **B** Base distribution. **C** Mean reads quality distribution

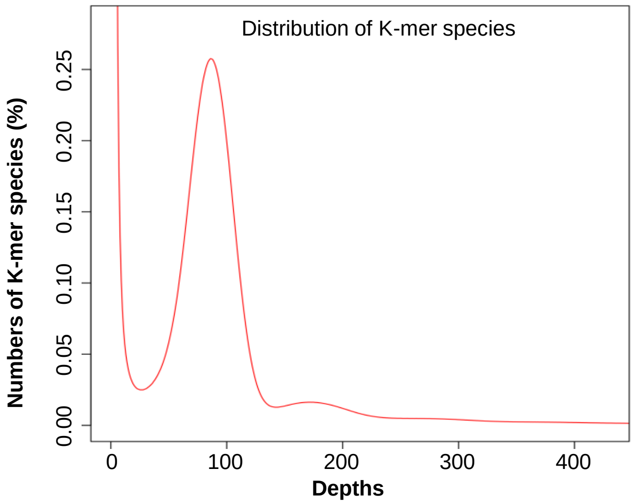

Supplementary Fig. 3 K-mer frequency distribution of sequencing reads of *P. floridana*. The 21-mer frequency (x-axis) is plotted against the occurrence of the k-mer (y-axis)

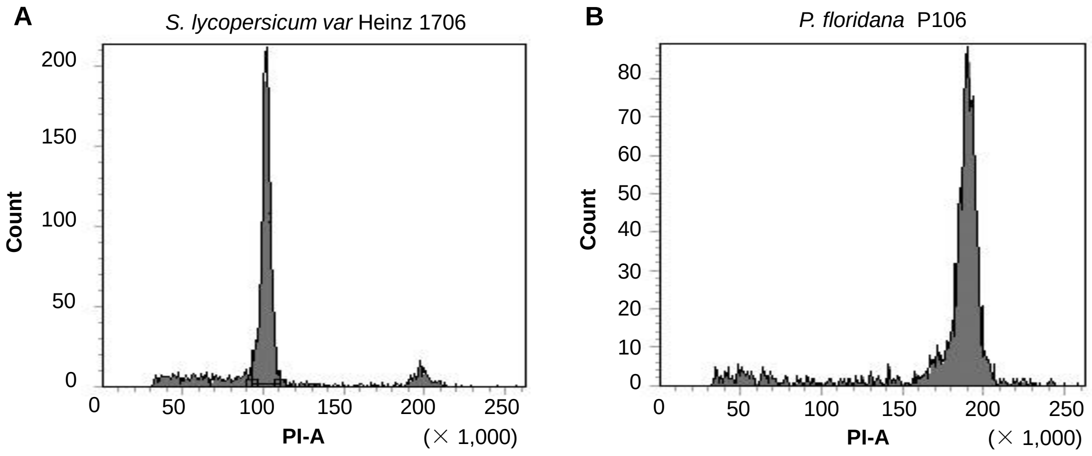

C

	PI-A mean	Genome size (Mb)
S. lycopersicum var Heinz 1706	135.1765±9.11	900.00
P. floridana P106	231.3681±15.04	1540.44

Supplementary Fig. 4 Evaluation of genome size by cell flow cytometry. A Flow cytometric analysis of *S. lycopersicum var* Heinz 1706 serving as a reference. **B** Flow cytometric analysis of *P. floridana* P106. The x-axis shows the relative DNA content, and the y-axis shows the calculated strength of the fluorescence signal. **C** Statistical analysis of *P. floridana* P106 genome size. The nuclear DNA content (in pg) of *P. floridana* samples was estimated according to the equation: 1C nuclear DNA content = (1C reference in pg × peak means of sesame) / (peak mean of reference)

A

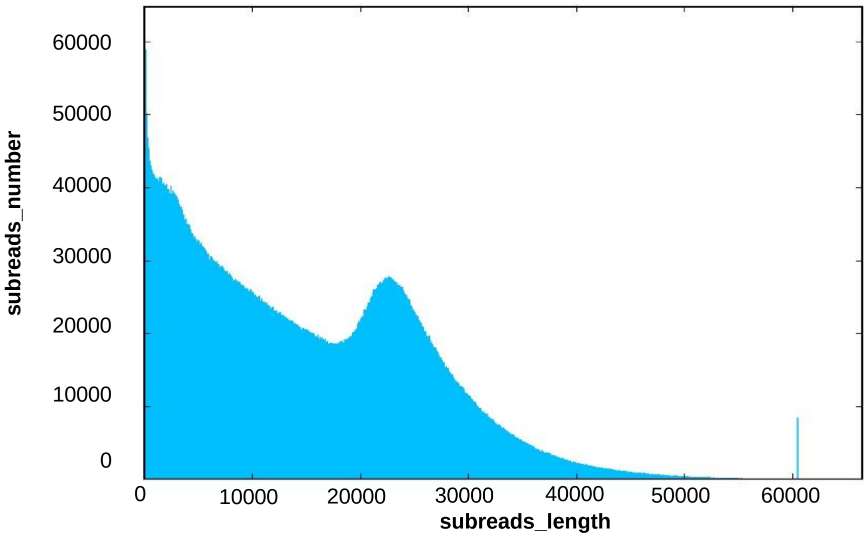

B

Sample	Library insert (kb)	Subreads statistics				Physical coverage (X)
		Cells	Mean Length (bp)	N50 (bp)	Base number (Gb)	
P. floridana P106	20	5	14,900.40	22,875	125	90

Supplementary Fig. 5 Statistical analysis results of PacBio sequencing data. **A** Subreads number and length distribution. **B** Filtering statistical results

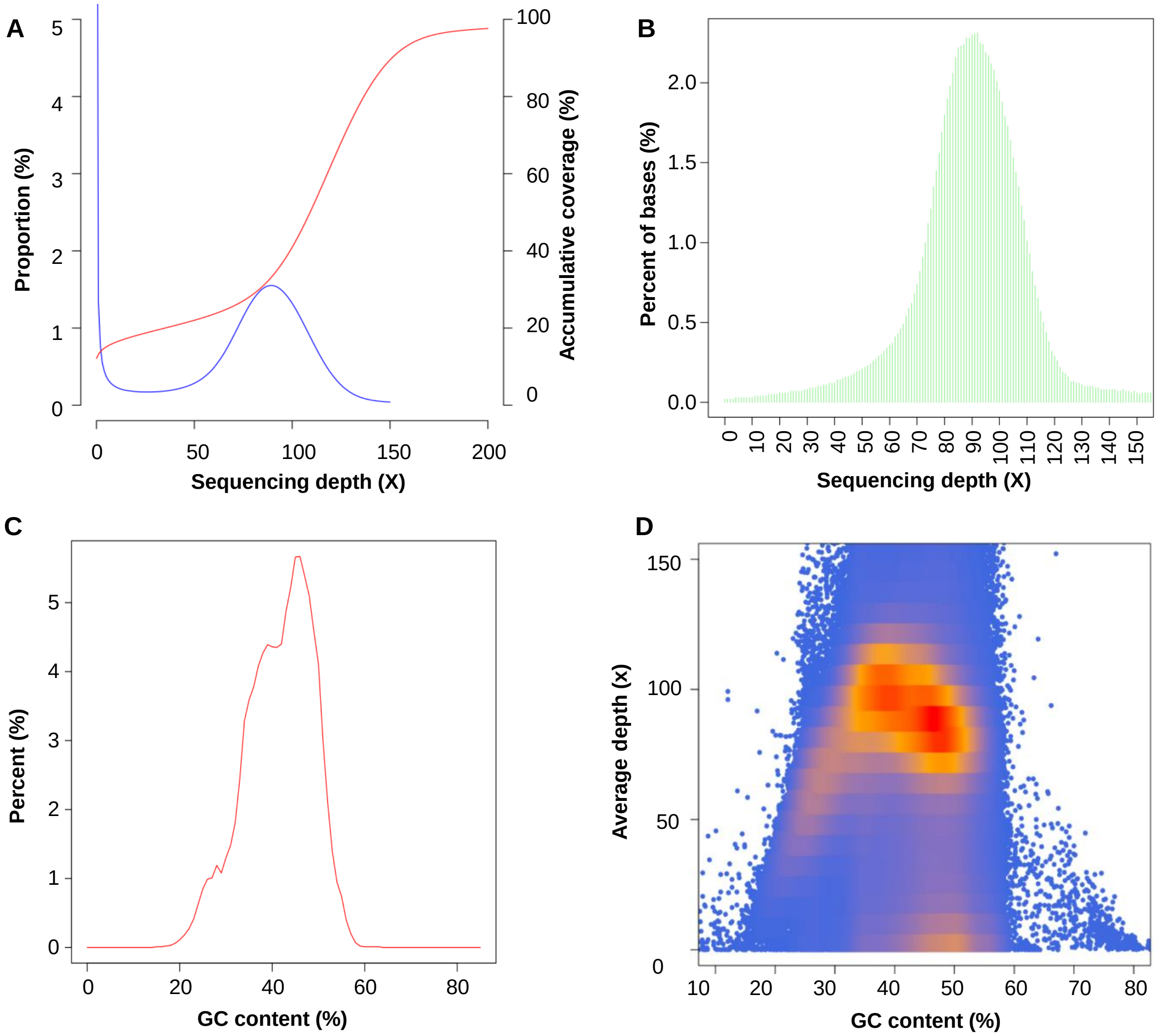

Supplementary Fig. 6 Statistical analysis results of genome assembly. A The frequency distribution of genome depth (blue) and accumulative coverage (red). **B** The frequency distribution of genome depth of sequencing reads. **C** The frequency distribution of the GC content. **D** The frequency distribution of the content and depth of GC. The abscissa represents GC content (in 10 kb windows), and the ordinate represents the ratio of the number of GC corresponding windows to the total number of windows

A

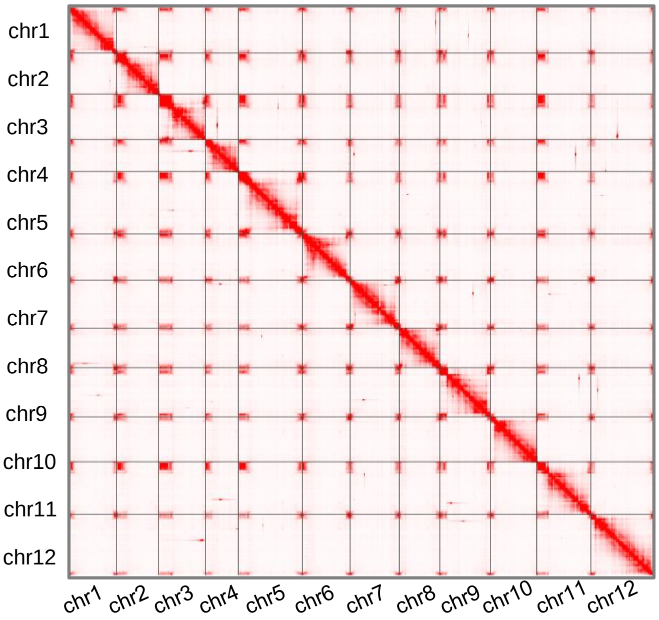

B

Pseudomolecule	Length
Chr01	112,228,426
Chr02	98,634,447
Chr03	107,648,561
Chr04	78,307,290
Chr05	149,502,788
Chr06	113,373,659
Chr07	114,608,531
Chr08	96,491,055
Chr09	119,292,486
Chr10	109,913,245
Chr11	126,491,088
Chr12	146,391,336
Total anchored	1,372,882,912
Unanchored	16,449,599

Supplementary Fig. 7 Results of Hi-C analyses in *P. floridana*. A The genomic global Hi-C interaction heat map. Contact matrices of the 12 chromosomes of the final *P. floridana* P106 assembly. The darker the color, the stronger the interaction signal. Strong Hi-C signals were enriched at both ends of each chromosome. The anchor rate of scaffold: 1,372,882,912 / (16,449,599 + 1,372,882,912) = 98.82%. **B** Length of each pseudochromosome

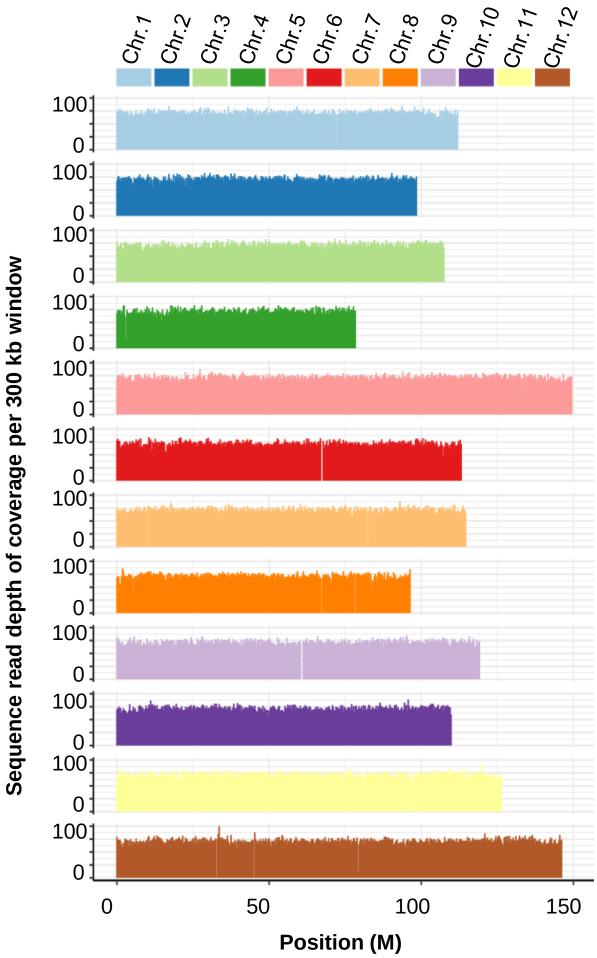

Supplementary Fig. 8 Sequence mapping profile in *P. floridana*. The y-axis shows the depth of PacBio subreads coverage along the non-overlapping 300 kb windows, and the x-axis shows the length of the 12 chromosomes of *P. floridana*. Chromosomes are listed in numerical order and are represented by the corresponding colors

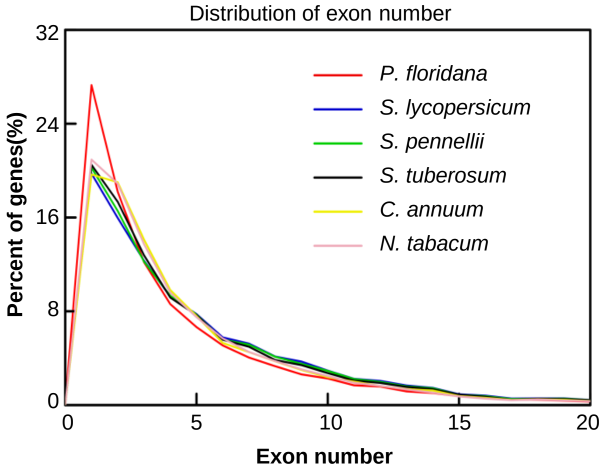

Supplementary Fig. 9 The frequency distribution of predicted exon numbers among solanaceous species.

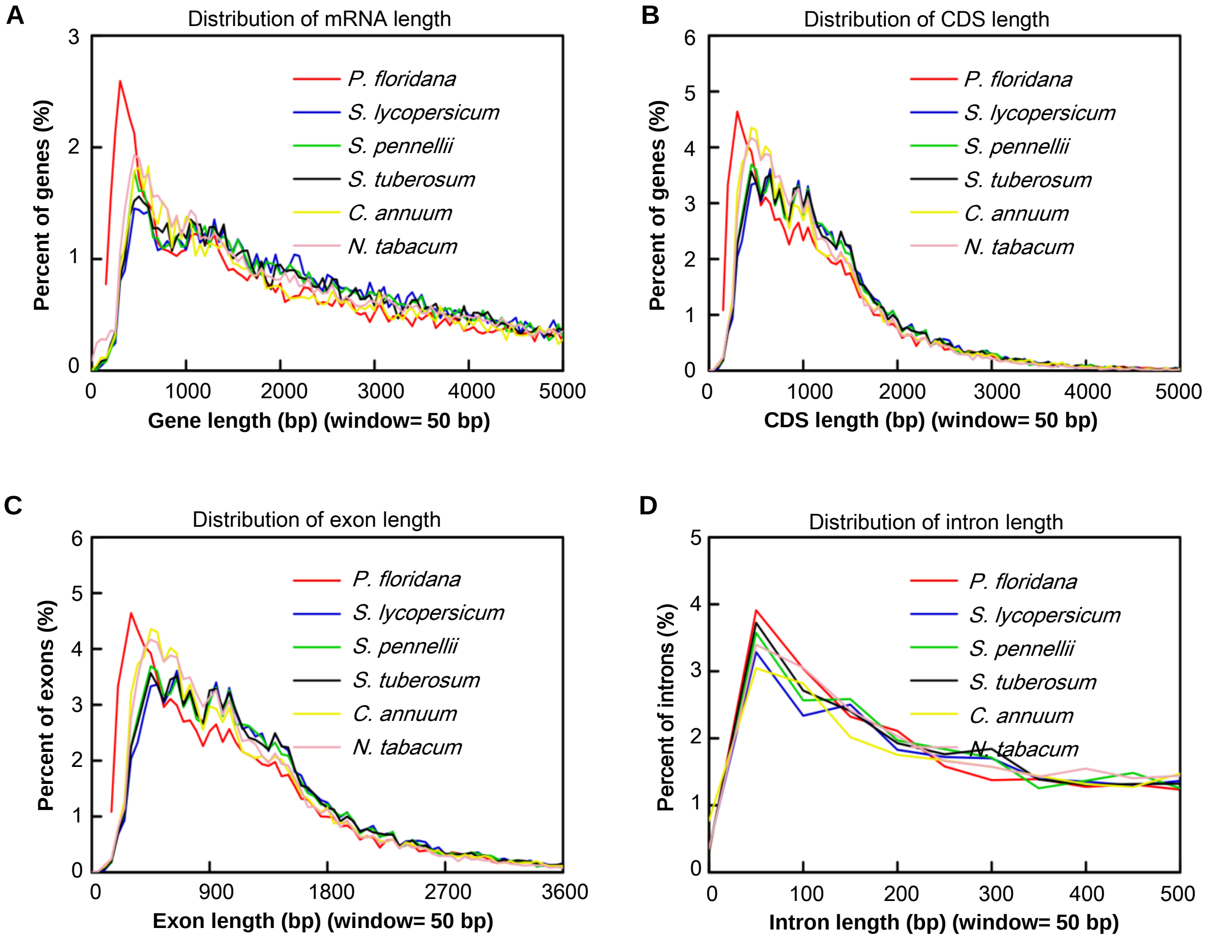

Supplementary Fig. 10 The frequency distribution of various predicted gene features among solanaceous species. **A** mRNA length. **B** CDS length. **C** Exon length. **D** Intron length

A

Database	Count	Percentage (%)
BLASTP	22,939	71.52
BLASTX	22,714	70.82
GO	23,092	71.99
KO	8,463	26.39
Map	5,372	16.75
NR	29,491	91.94
NT	26,293	81.97
PFAM	23,014	71.75
eggNOG	18,902	58.93
Annotated	29,938	93.34
Unannotated	2,137	6.66
Total	32,075	100.00

B

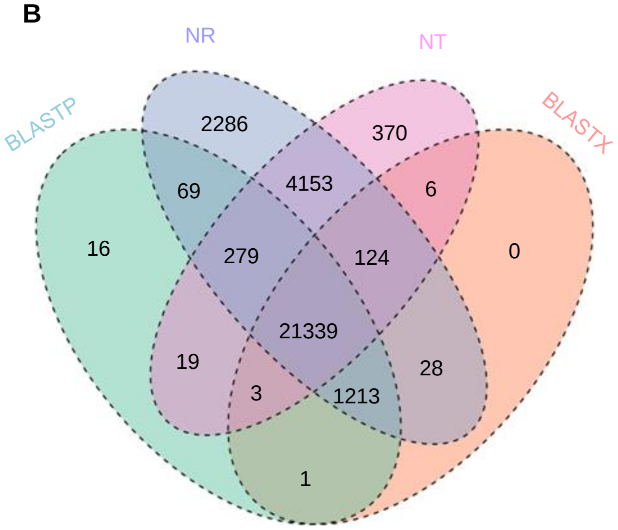

Supplementary Fig. 11 Gene functional annotation of the *P. floridana* genome. **A** Summary of gene functional annotation. **B** Venn diagram showing the numbers of unique and shared genes using different databases

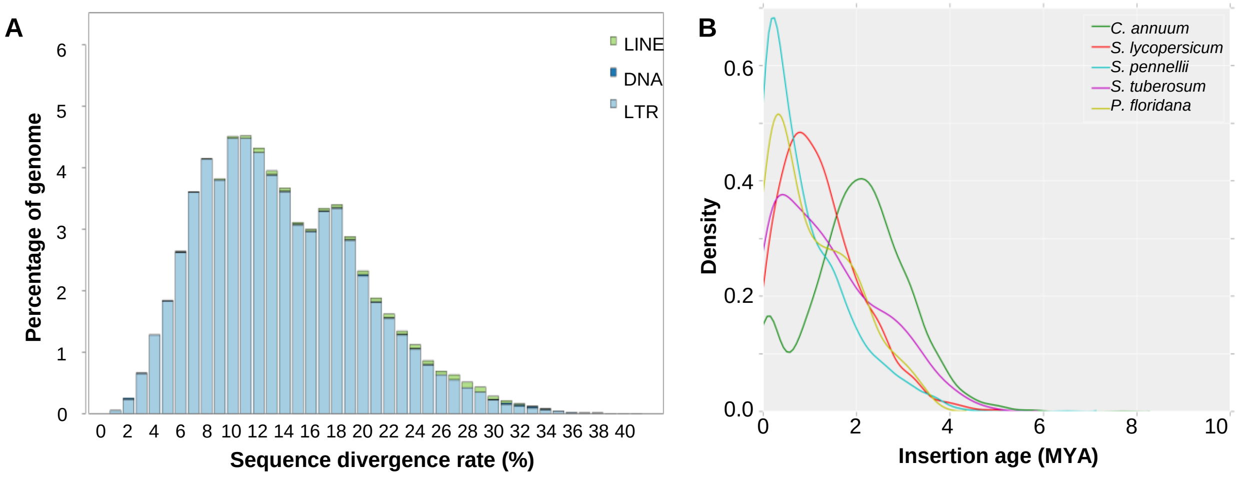

Supplementary Fig. 12 The frequency distributions of TE repeats and LTR insertion times. A The frequency distribution of major TE classes sequence divergence in *P. floridana*. **B** Insertion times of LTR retrotransposons in the *P. floridana*, *S. tuberosum*, *S. pennellii*, *S. lycopersicum,* and *C. annuum* genomes

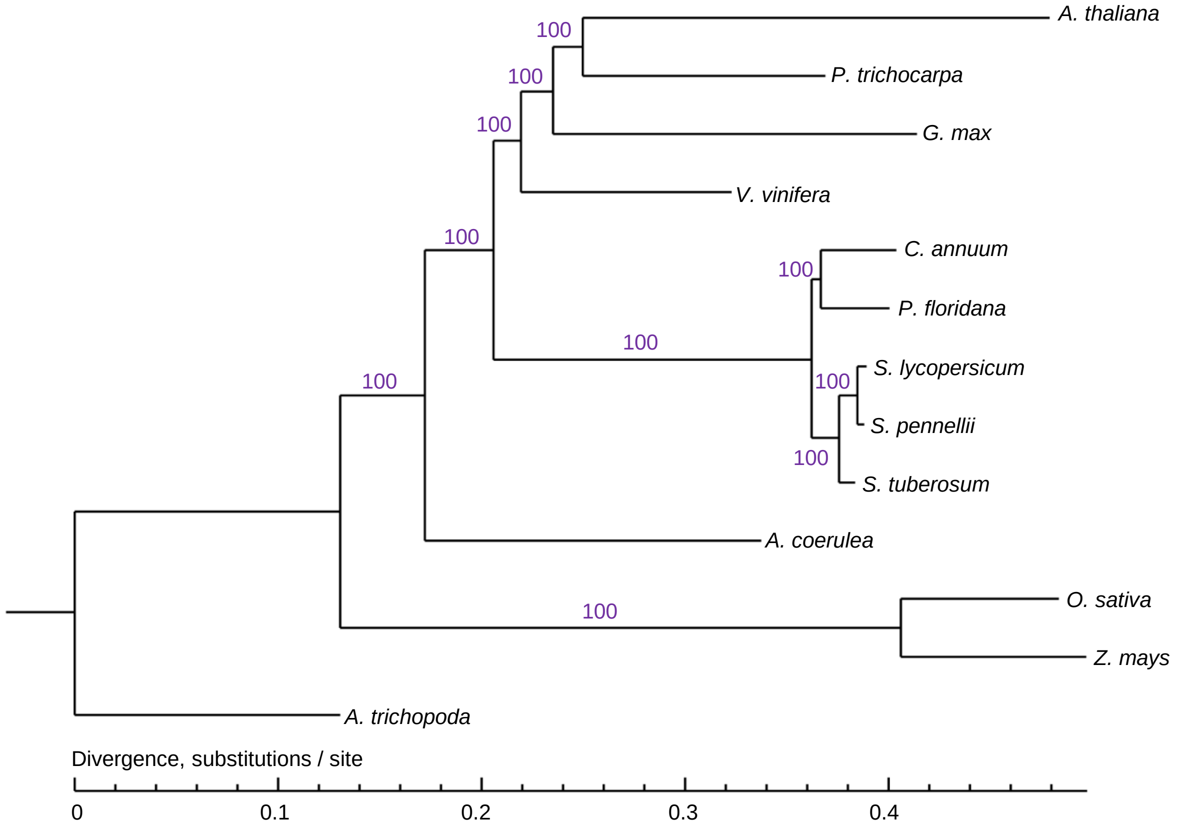

Supplementary Fig. 13 Phylogenetic tree for 13 plant species. The phylogenetic tree was constructed using 7,553 single-copy orthologous genes by the maximum likelihood method. The longer the length of the branch, the longer the divergence time. The closer the branches on the phylogenetic tree, the closer the predicted genetic relationship. The numbers before the binary structure on the phylogenetic tree represent the Bootstrap support numbers, and a number greater than 85 denotes good support for the result

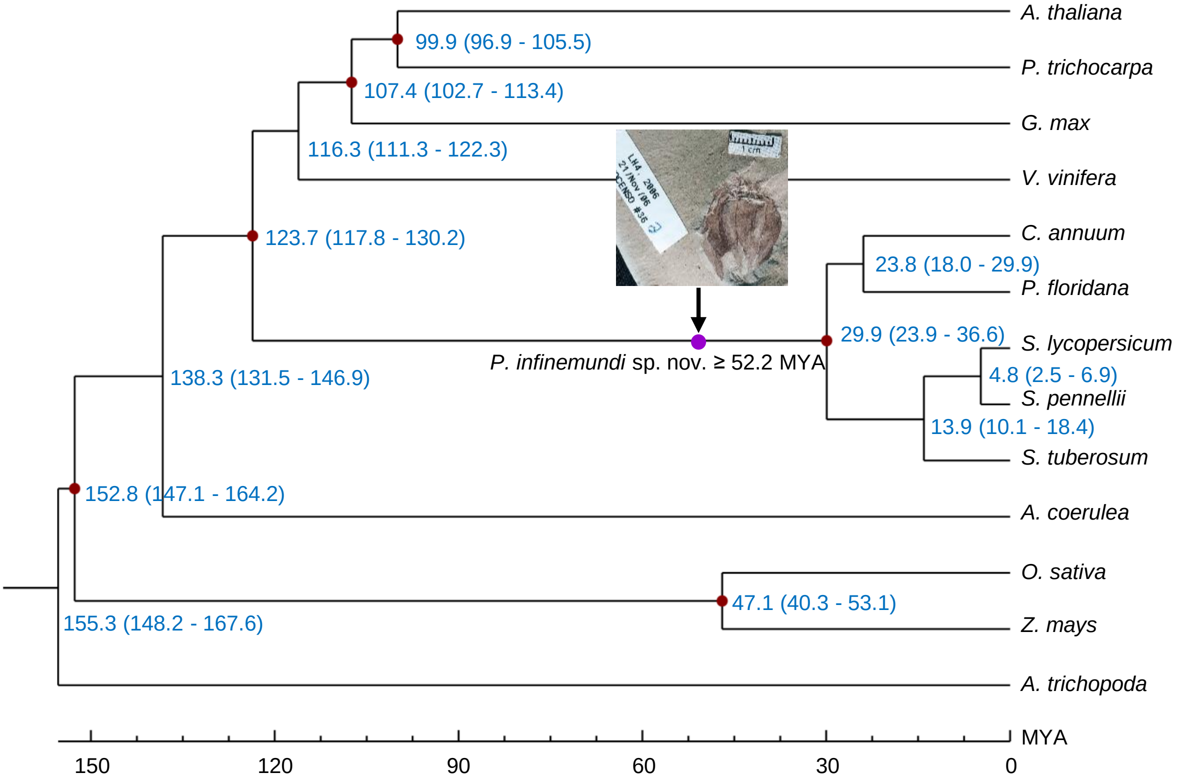

Supplementary Fig. 14 Divergence time estimation of the selected species. The blue number on the node position represents the divergence time of each species in millions of year ago (MYA). The numbers in parentheses indicate the confidence range of the divergence time; these can be used to estimate the divergence time of target species and other species. Divergence times were estimated by using PAML MCMCTREE and implementing the approximate likelihood calculation method. *A. trichopoda* was used as the outgroup. The 52.2-million-year-old lantern fruit of *P. infinemundi* sp. nov (Wilf et al., 2017) is indicated

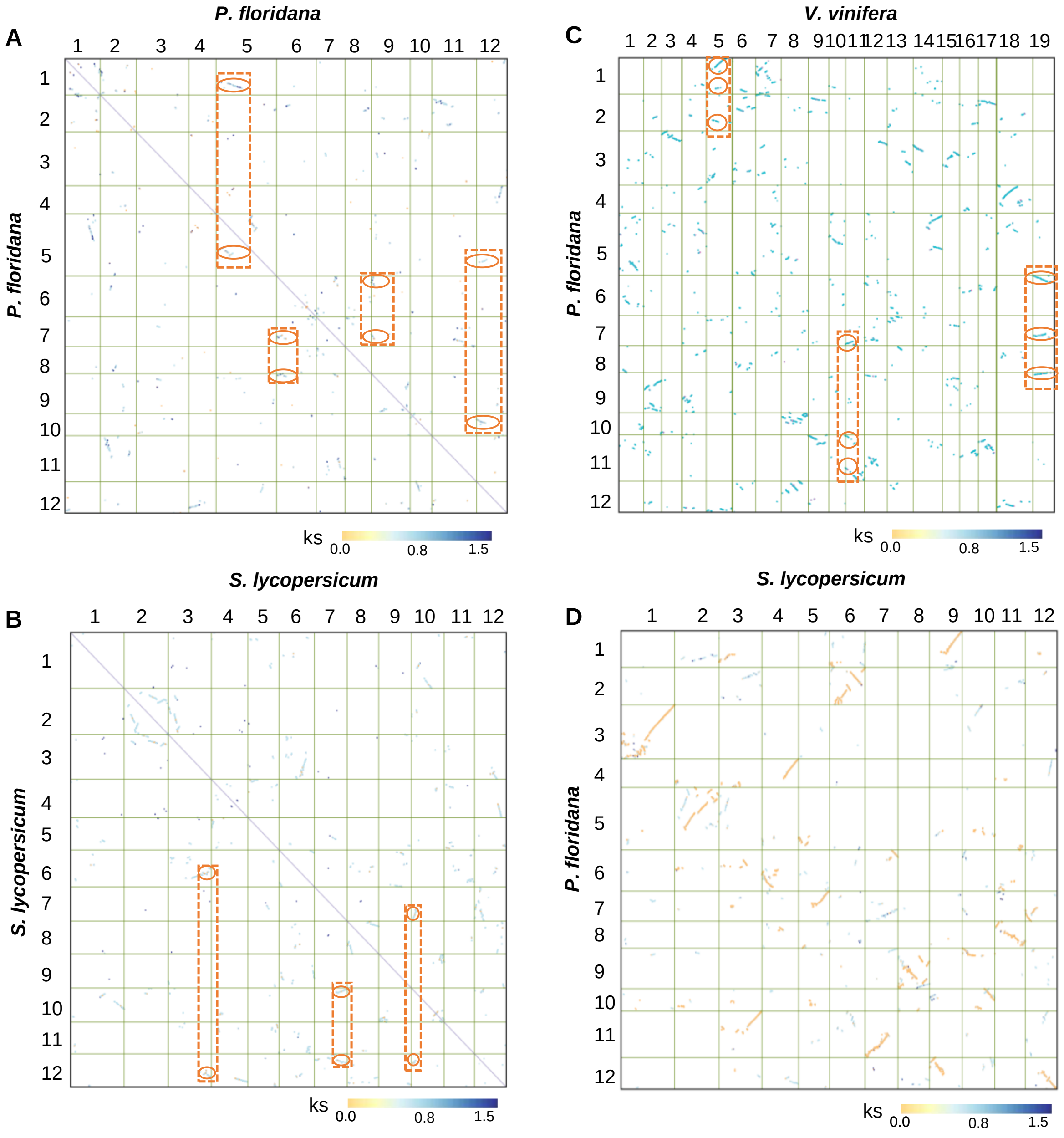

Supplementary Fig. 15 The triplication events present in the *P. floridana* P106 genome. **A** Dot plot matrix displaying the syntenic paralogs within the *P. floridana* genome. The circles within dashed lines indicate the triplication imprints in the *P. floridana* genome. **B** Dot plot matrix displaying the syntenic paralogs within *S. lycopersicum*. The circles indicate the triplication imprints in the *S. lycopersicum* genome. **C** Dot plots of orthologs between *P. floridana* and grape genomes. Circles show a 1:3 synteny relationship between grape and *P. floridana*. **D** Dot plots of orthologs between *P. floridana* and *S. lycopersicum* genomes

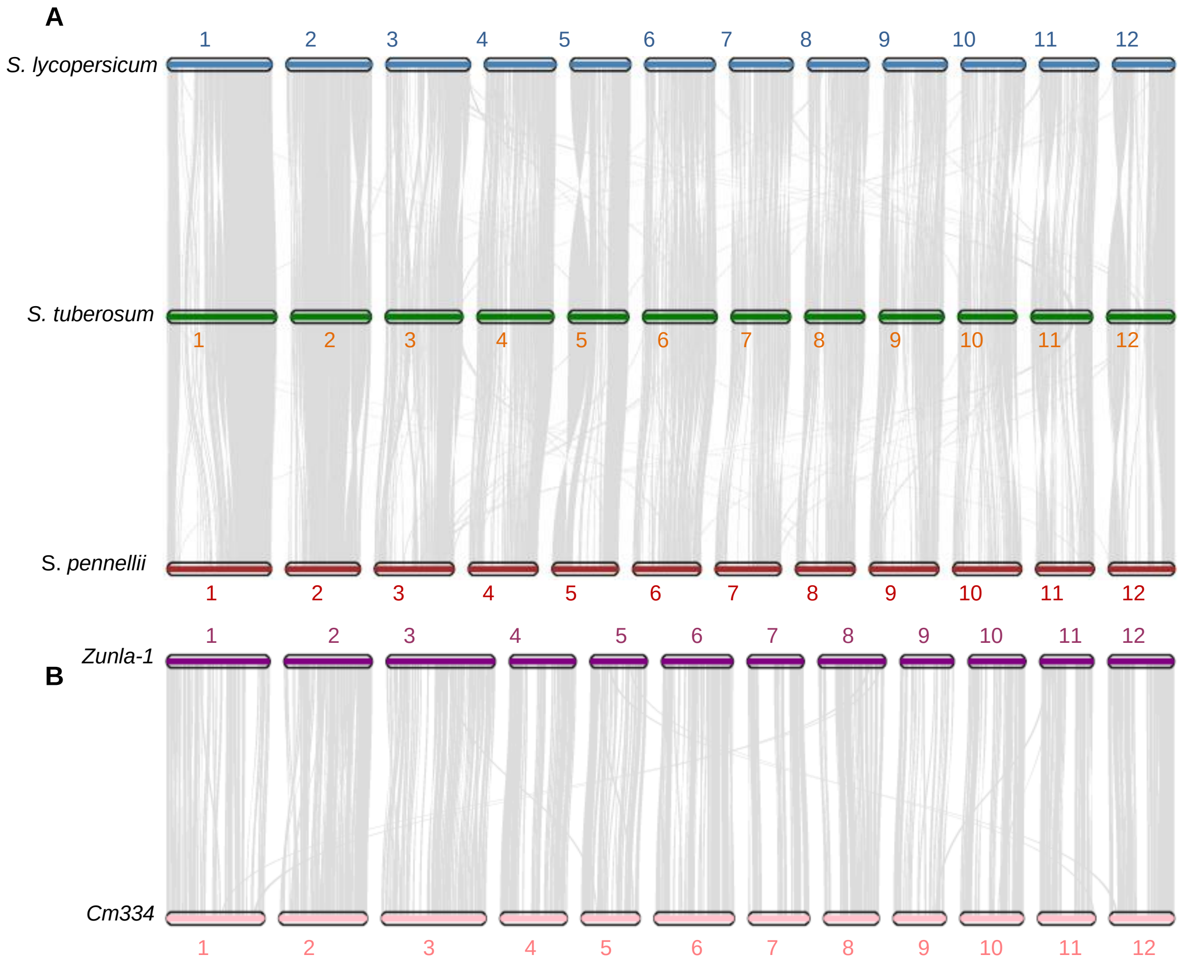

Supplementary Fig. 16 Genomic synteny comparison. A Synteny of *S. tuberosum*, *S. pennellii,* and *S. lycopersicum*. **B** Synteny of *C. annuum* cultivars CM334 and Zunla-1. Lines between chromosomes (Chr) show syntenic regions. Grey wedges in the background (**B**) highlight syntenic blocks between *C. annuum* cv. CM334 and *C. annuum* cv. Zunla-1

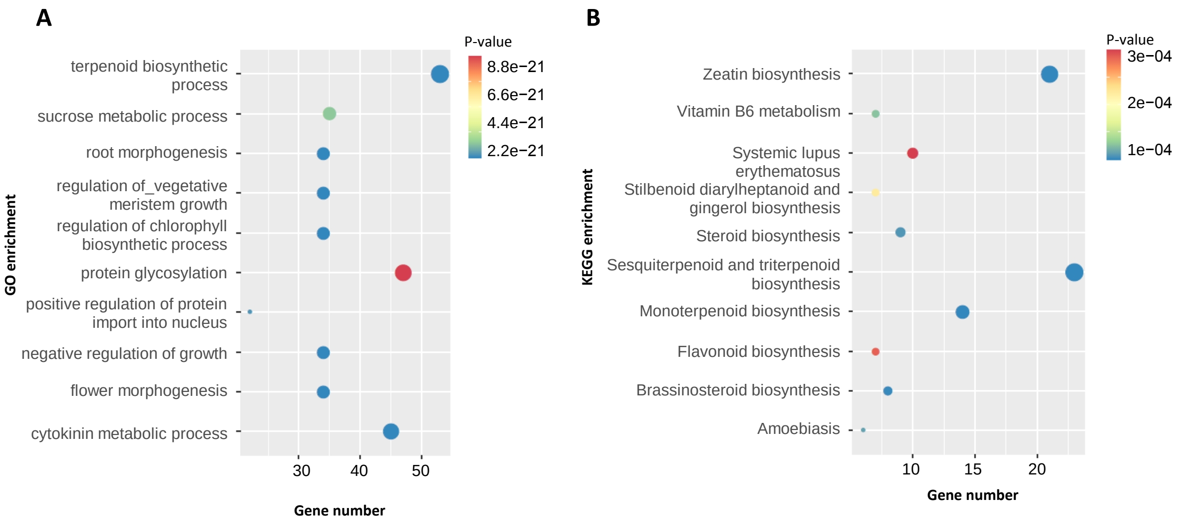

Supplementary Fig. 17 Enrichment analysis of significantly expanded gene families in *P. floridana.* **A** GO enrichment analyses. The top 10 GO terms are shown. *P* values represent the significance of the enrichment. The source data are listed in Supplementary Table 20. **B** KEGG analysis. The top 10 enriched KEGG terms are shown. *P* values represent the significance of the enrichment. The source data are listed in Supplementary Table 21. Circles indicate the target genes, and the size is proportional to the number of genes

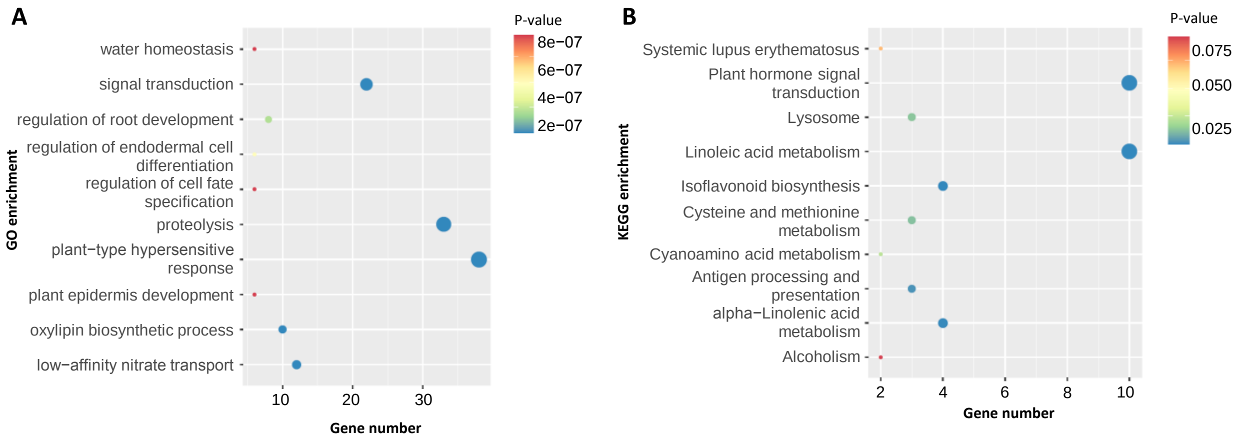

Supplementary Fig. 18 Enrichment analysis of significantly contracted gene families in *P. floridana*. **A** GO enrichment analyses. The top 10 GO terms are shown. *P* values represent the significance of the enrichment. The source data are listed in Supplementary Table 22. **B** KEGG analysis. The top 10 enriched KEGG terms are shown. *P* values represent the significance of the enrichment. The source data are listed in Supplementary Table 23. Circles indicate the target genes, and the size is proportional to the number of genes

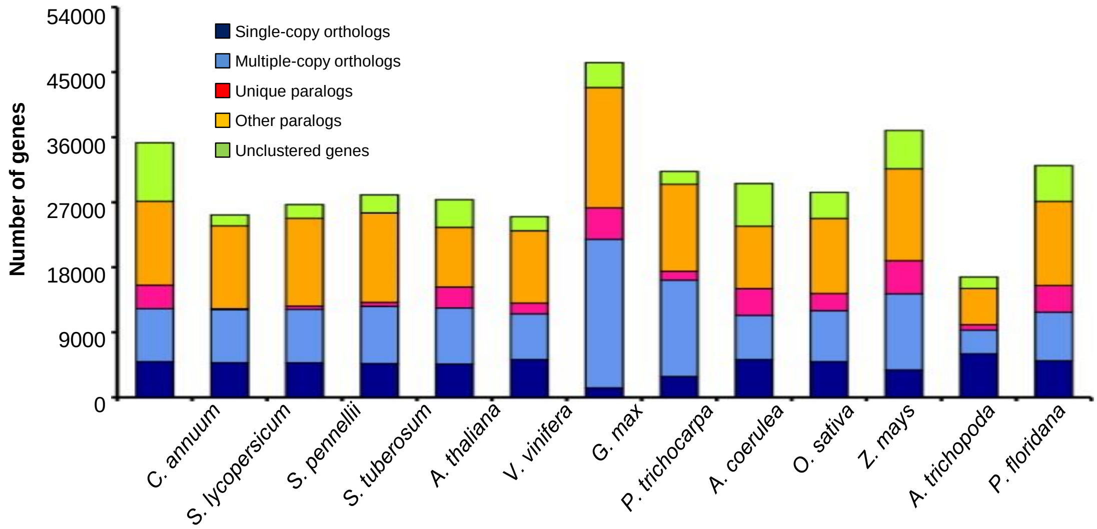

Supplementary Fig. 19 Gene family comparisons among 13 representative angiosperm species. Distribution of single-copy orthologs (dark blue), multiple-copy orthologs (pale blue), unique genes (red), other paralogs (orange), and other unclassified genes (green) in *P. floridana* and 12 other plant species as indicated

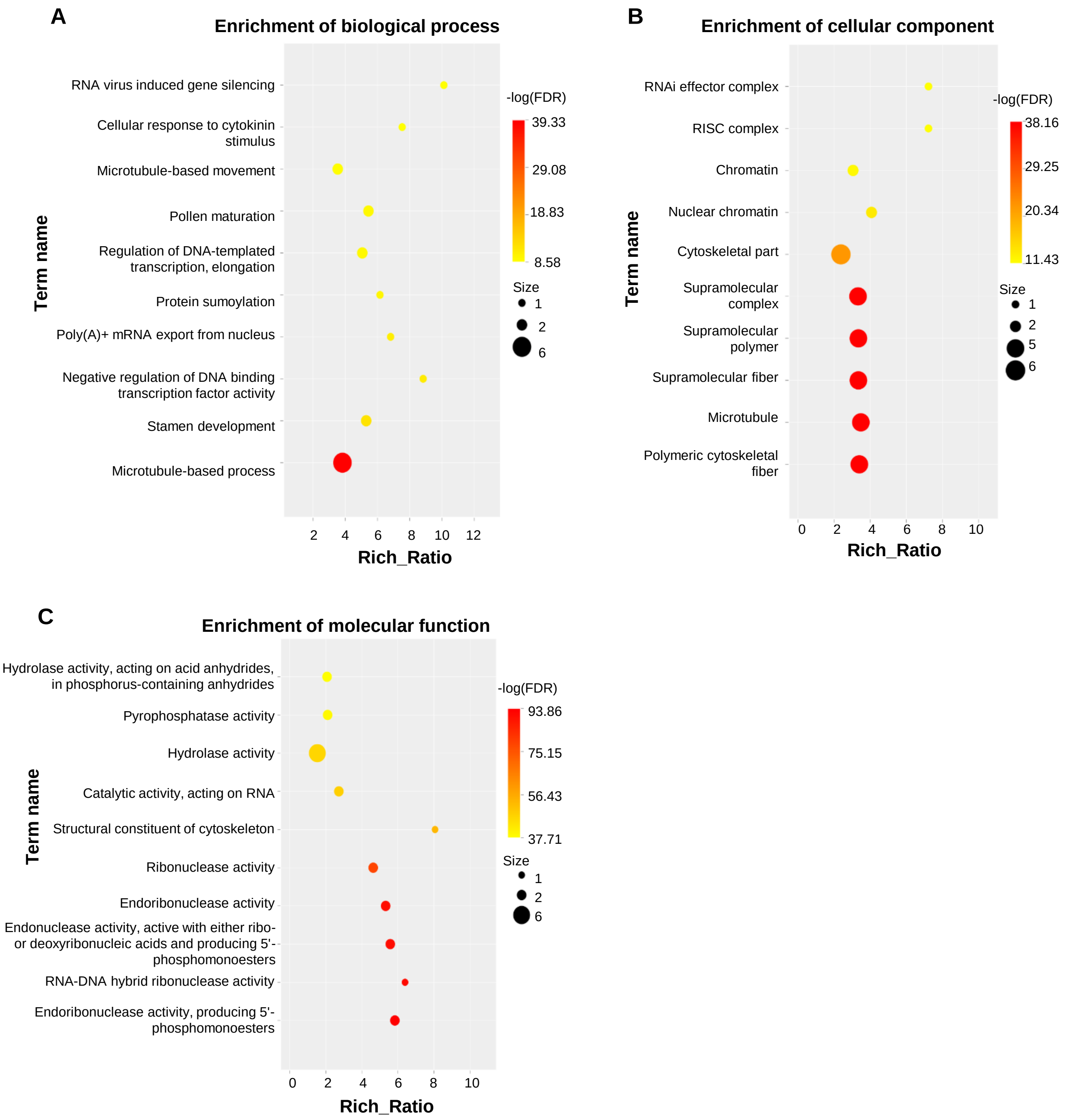

Supplementary Fig. 20 GO-enrichment analyses of *Physalis* unique gene families. **A** The biological process enrichment. **B** The cellular component enrichment. **C** The molecular function enrichment. A total of 3671 genes were used in the GO analysis. In each analysis, the top 10 GO terms are shown. *Q* values represent the significance of the enrichment. Circles indicate the target genes, and the size is proportional to the number of genes. The source data are listed in Supplementary Table 26

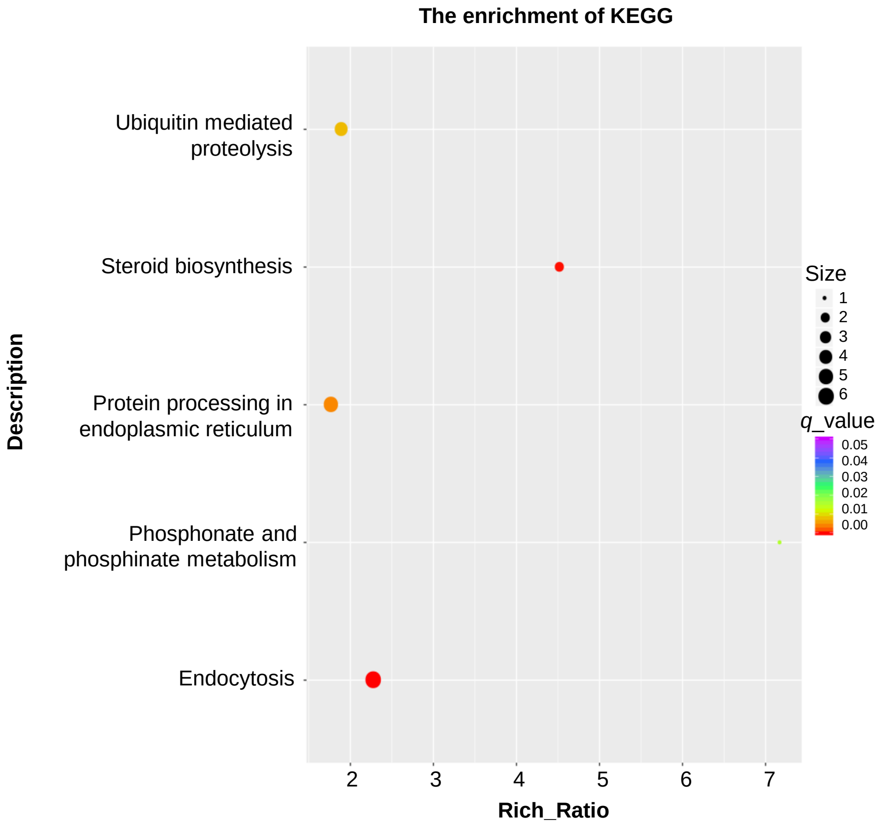

Supplementary Fig. 21 KEGG analysis of the unique gene families in *P. floridana*. The top five enriched KEGG terms are shown. The source data of the unique gene families are listed in Supplementary Table 27

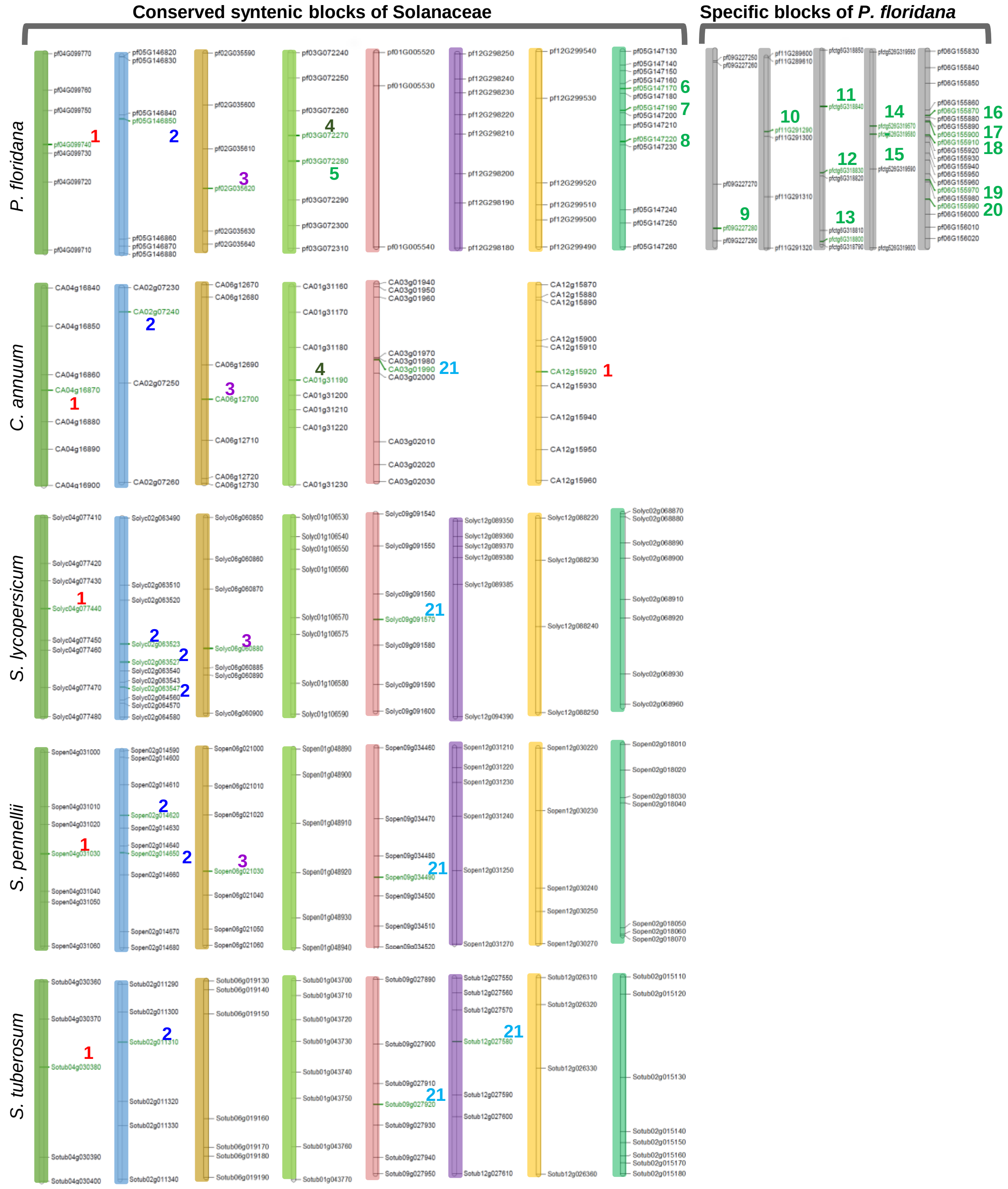

Supplementary Fig. 22 The genomic location of *SQE* genes detected in the Solanaceae. Collinear blocks are represented by the same color in each column. SQE genes are highlighted in green on each block. The specific genomic regions of *P. floridana* are shown with grey blocks. Altogether, 21 distinct SQE genes were found within the Solanaceae genomes; these are labeled as 1–21 in the figure. The putative orthologs of the Solanaceae conserved SQE genes are indicated with different colors but with the same label number, while the *P. floridana*-specific *SQE* genes are indicated in green and are located in specific genomic blocks of *P. floridana.* The orthologs were grouped by a combination of syntenic and phylogenetic analyses (Supplementary Fig. 23)

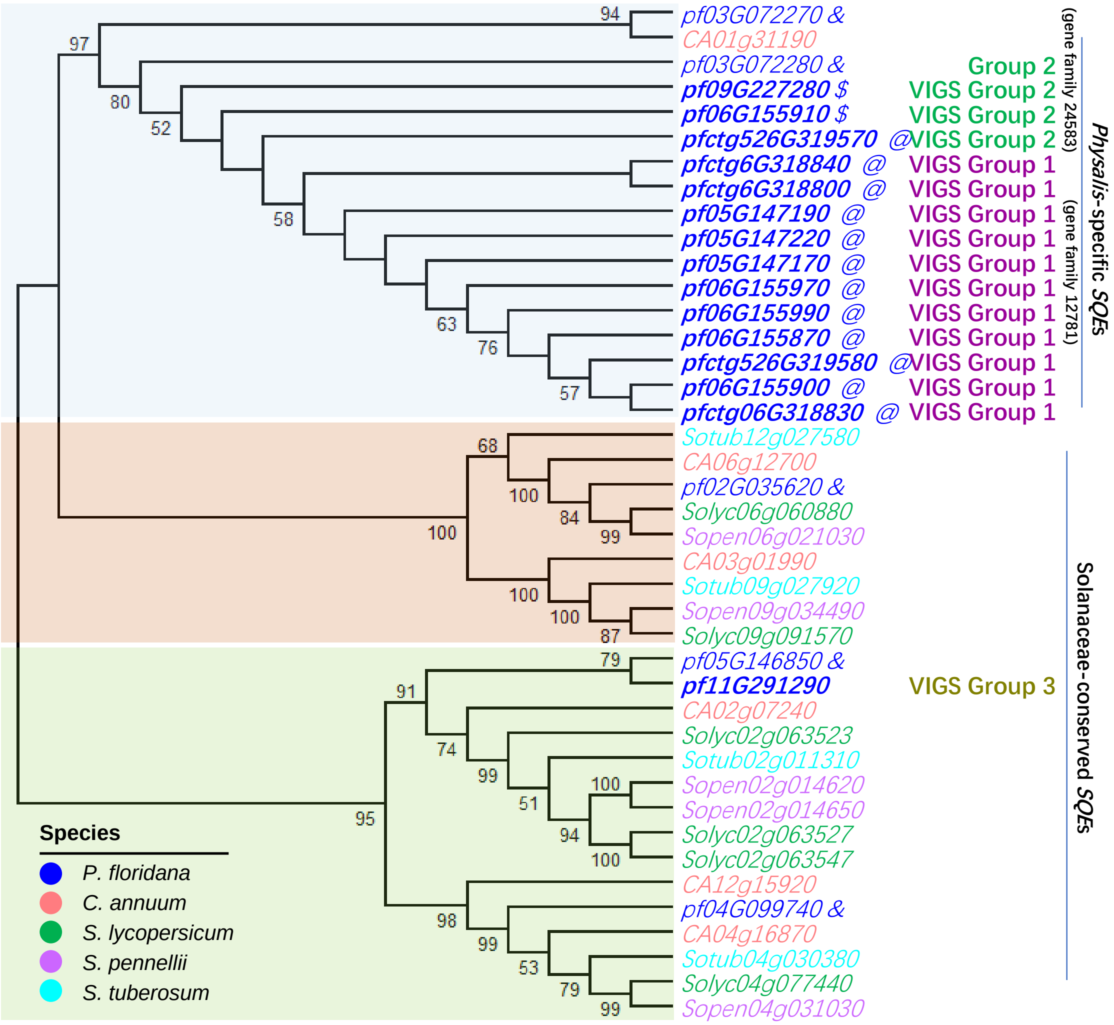

Supplementary Fig. 23 The evolution of squalene epoxidase genes within the Solanaceae. Most of the *Physalis*-specific *SQE* genes had relatively high homology and were grouped with pf03G072280. The *Physalis* genes marked with "&" are those containing some motifs found in *SQE* genes. Fourteen *PfSQEs* are *Physalis*-specific genes that were also identified from gene expansion analysis including gene family 12781 (12 members) and gene family 24583 (2 members) that are marked with "@" and "$", respectively. Fifteen SQEs (bold names) were divided into three groups for VIGS (VIGS 1–3) based on the sequence similarity in *P. floridana* P106 in the frame of the phylogeny. The tree was constructed using maximum likelihood in MEGA. Bootstrap values > 50% are shown

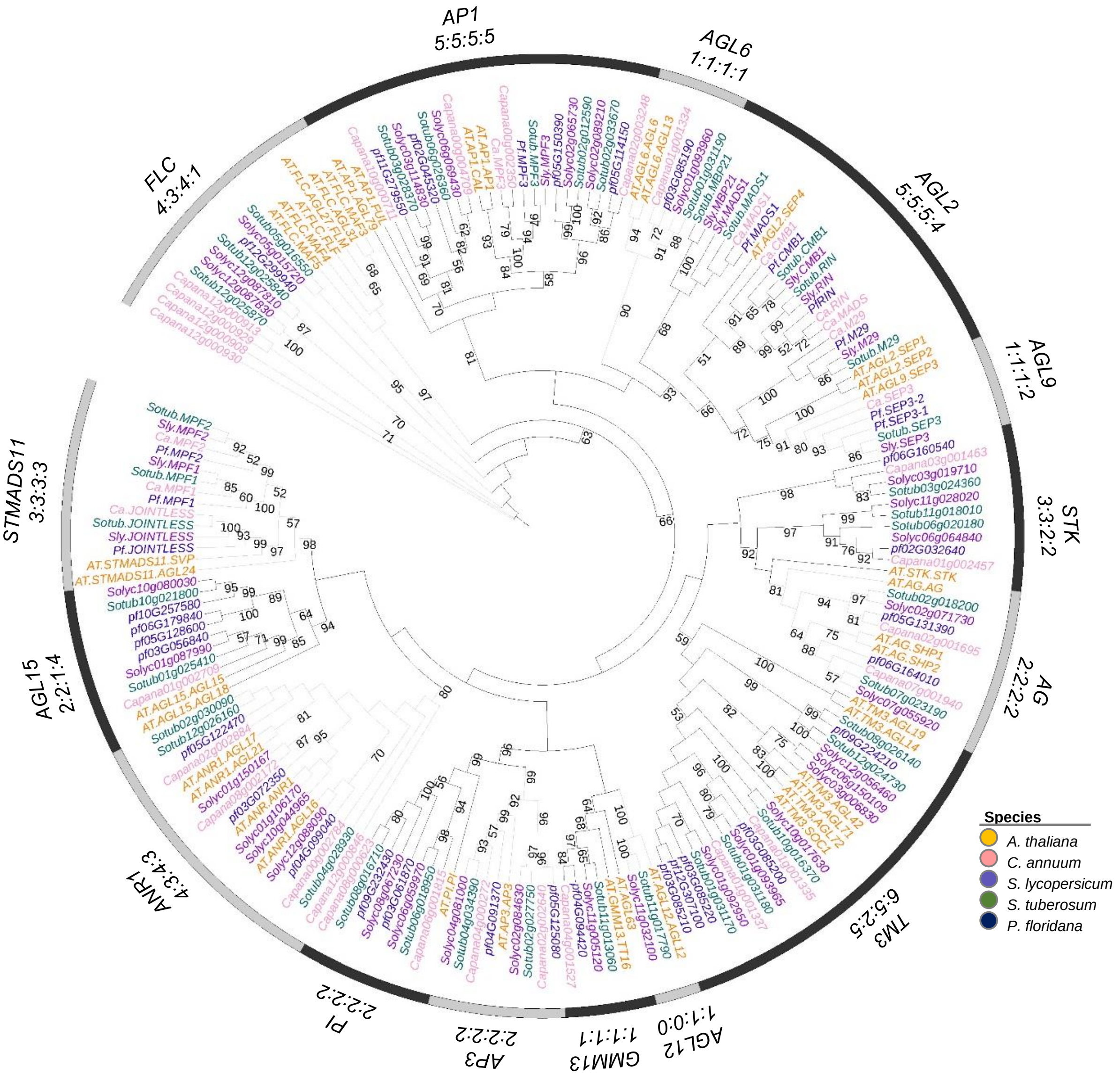

Supplementary Fig. 24 The phylogeny of MIKC type of MADS-box genes. Four species from the Solanaceae and *Arabidopsis thaliana* were included. The copy number of MADS-box genes in each lineage (*S. lycopersicum: S. tuberosum: C. annuum: P. floridana*) is marked. The tree was constructed using maximum likelihood in Raxml. Bootstrap values > 50% are shown

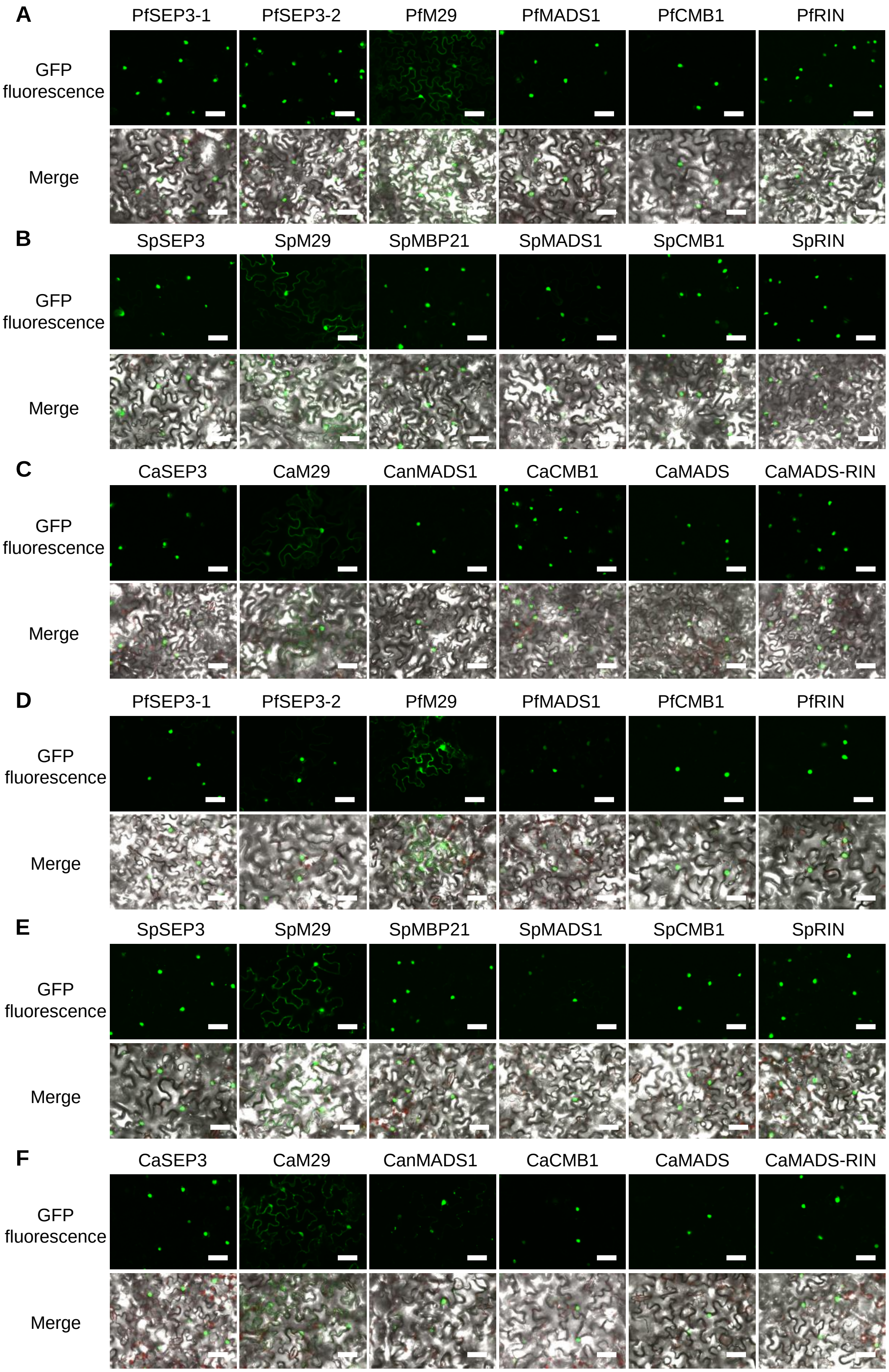

Supplementary Fig. 25 Subcellular localizations of solanaceous SEP-like proteins. A–C Subcellular localization of E-class MADS-box proteins of *P. floridana* (A), *S. pimpinellifolium* (B), and *C. annuum* (C) in tobacco leaf epidermal cells. **D–F** Subcellular localization of E-class MADS-box proteins of *P. floridana* (D), *S. pimpinellifolium* (E), and *C. annuum* (F) in *P. floridana* leaf epidermal cells. Bar = 50 μm

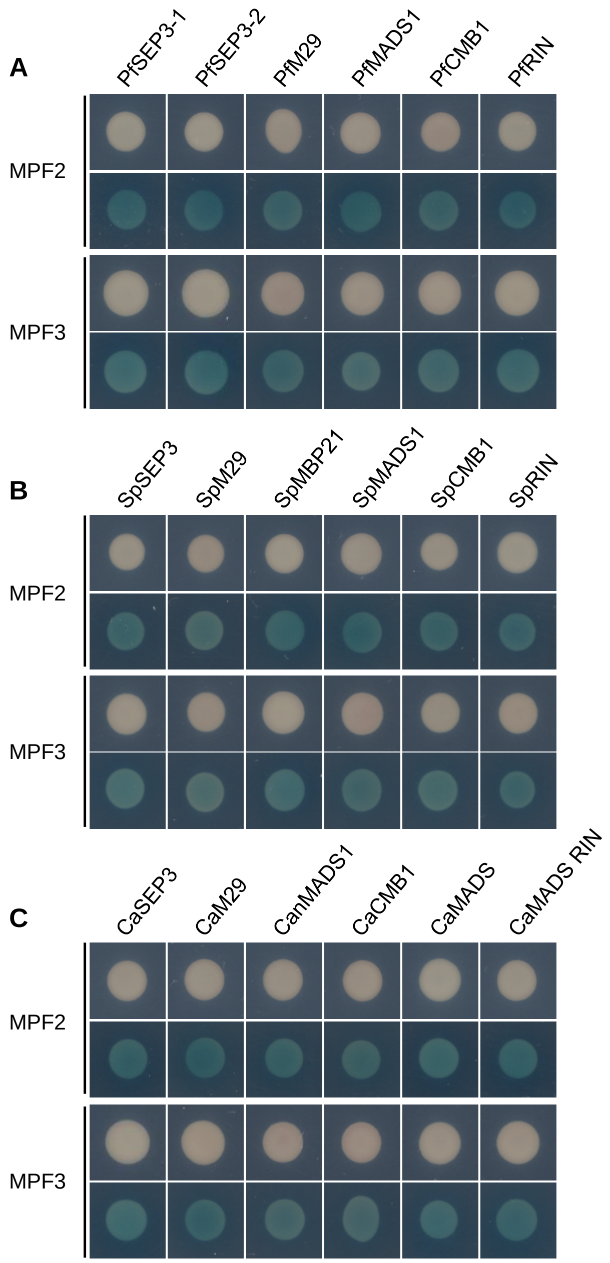

Supplementary Fig. 26 SEP-like proteins from three solanaceous species interact with MPF2 or MPF3 in yeast two-hybrid assays. A SEP-like proteins from *P. floridana.* **B** SEP-like proteins from *S. pimpinellifolium*. **C** SEP-like proteins from *C. annuum.* The first row of each group shows the yeast growth on SD medium lacking Trp, Leu, His, and Ade. The basal row of each group shows the yeast growth on SD medium lacking Trp, Leu, His, and Ade with X-α-Gal

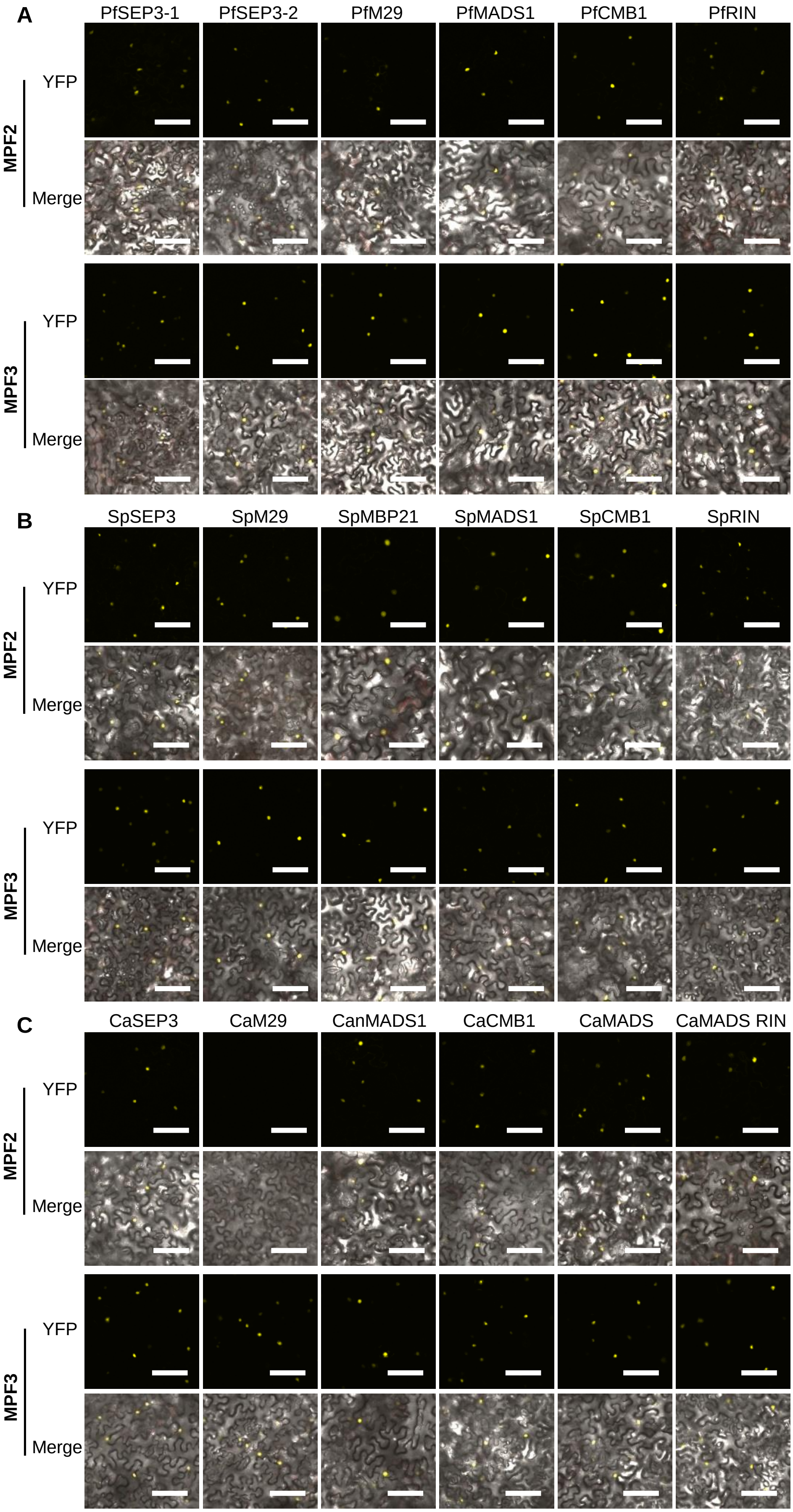
A
PfSEP3-1
PfSEP3-2
PfM29
PfMADS1
PfCMB1
PfRIN
MPF2
MPF3
YFP
Merge
B
SpSEP3
SpM29
SpMBP21
SpMADS1
SpCMB1
SpRIN
C
CaSEP3
CaM29
CanMADS1
CaCMB1
CaMADS
CaMADS RIN

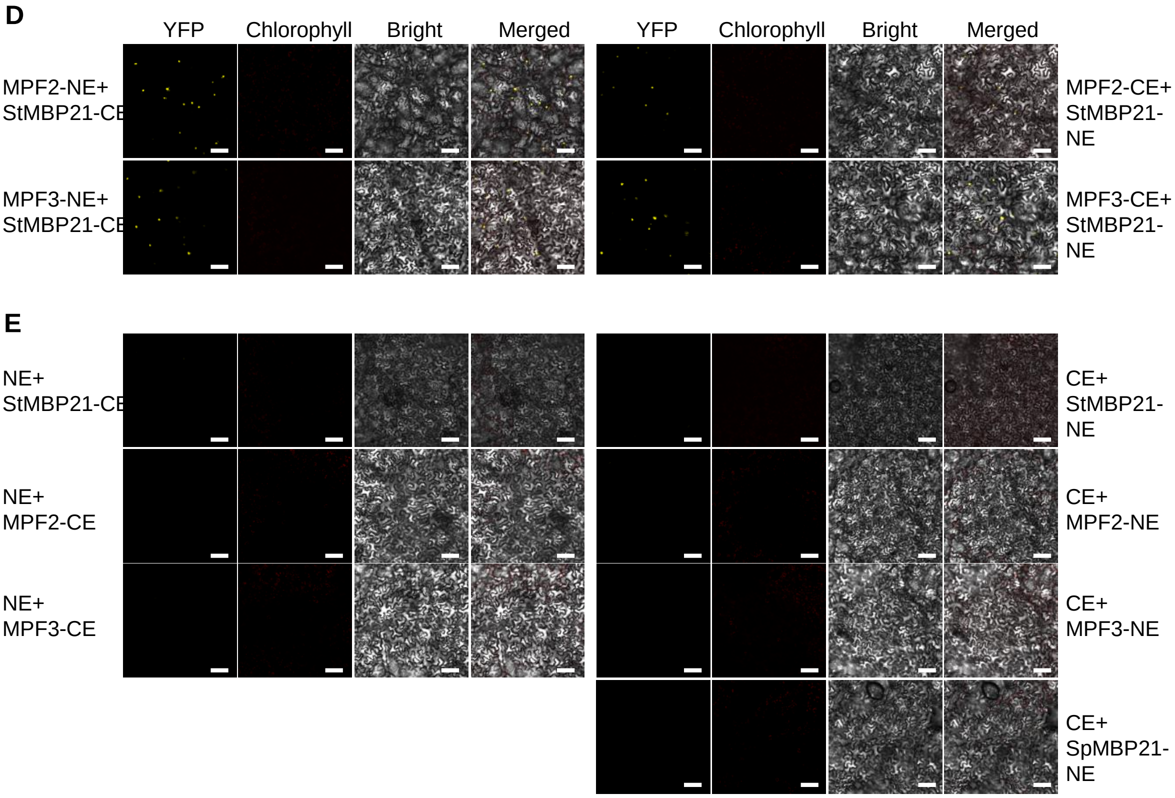

Supplementary Fig. 27 SEP-like proteins from three solanaceous species interact with MPF2 or MPF3 in BiFC analyses. A SEP-like proteins from *P. floridana.* **B** SEP-like proteins from *S. pimpinellifolium*. **C** SEP-like proteins from *C. annuum.* Interaction signals were detected in the tobacco leaf cells. Bar = 100 µm. **D** StMBP21 protein interacts with MPF2 and MPF3 in the leaf epidermal cells of tobacco. **E** Negative controls. Bar = 100 µm

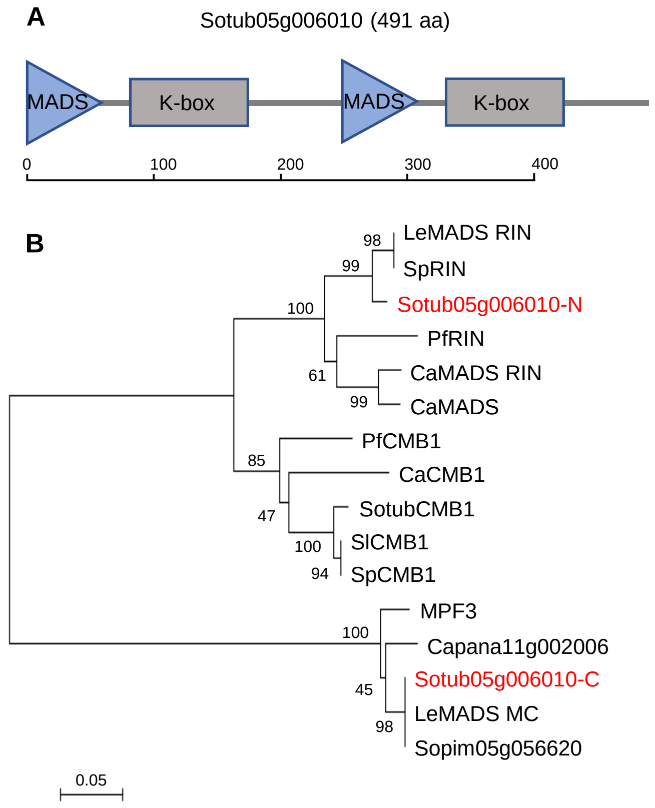

Supplementary Fig. 28 The domain and phylogeny of Sotub05g006010. **A** The domain structure. **B** The phylogeny of the two MADS-box genes on Sotub05g006010

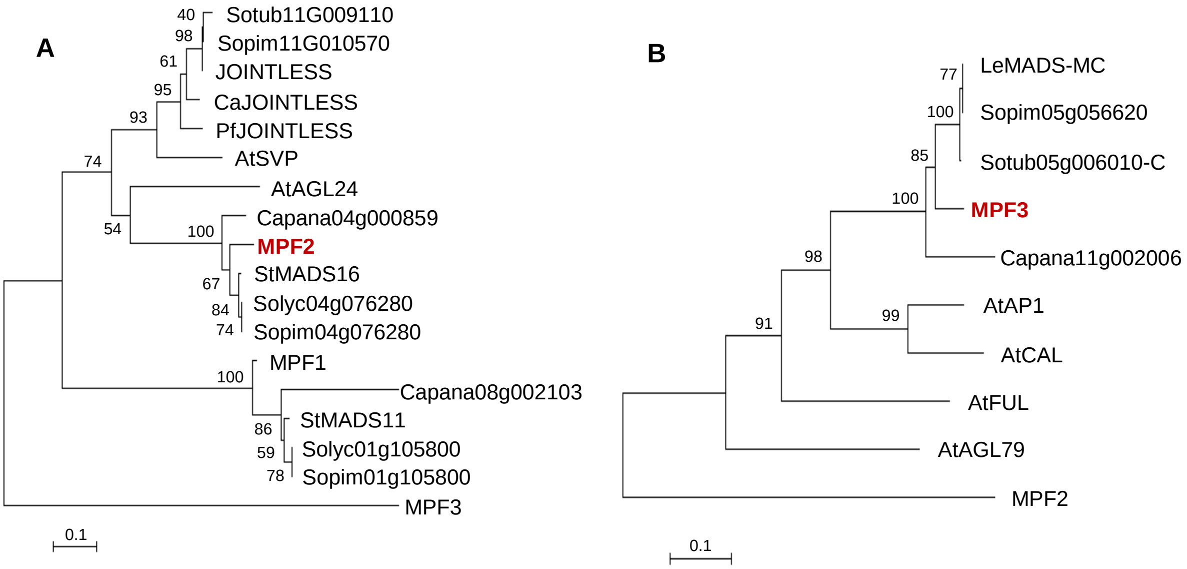

Supplementary Fig. 29 The phylogenetic trees for MPF2 and MPF3 orthologs in *S. lycopersicum, S. pimpinellifolium*, *C. annuum*, *S. tuberosum,* and *A. thaliana.* **A** Phylogenetic tree for MPF2-like proteins. **B** Phylogenetic tree for MPF3 and its orthologs

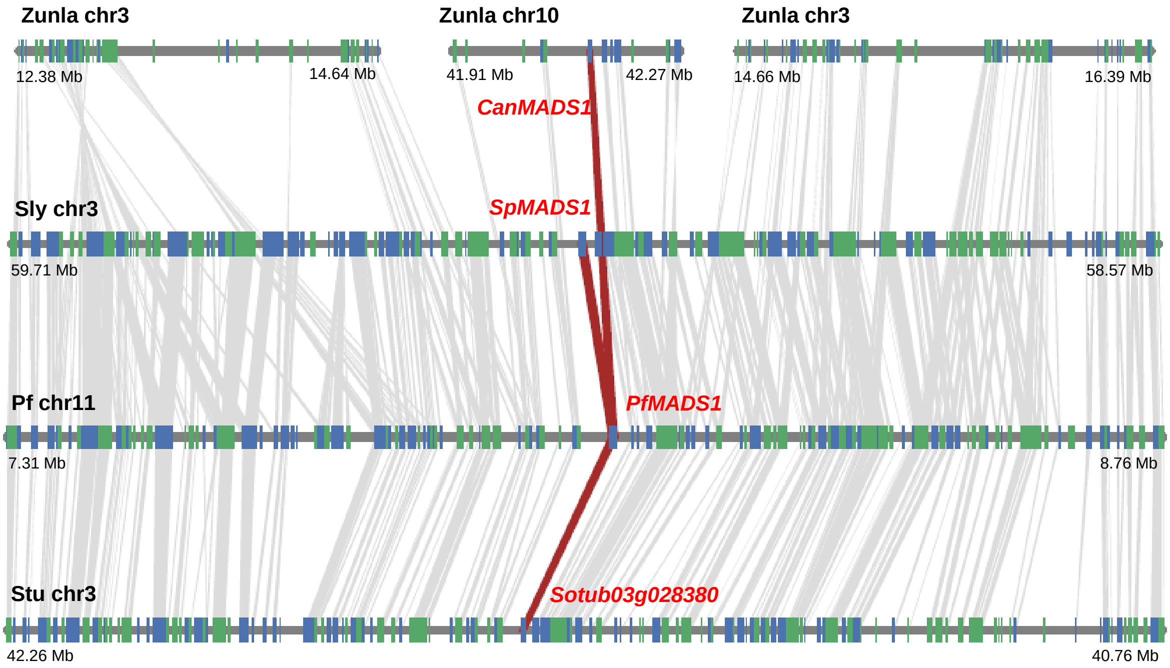

Supplementary Fig. 30 The microsynteny patterns of the MADS1 subclade among *C. annuum*, *S. lycopersicum, P. floridana,* and *S. tuberosum.* Green and blue rectangles represent predicted genes and their relative locations. Grey wedges connect matching gene pairs; orthologous gene pairs (MADS1-like) are highlighted in brown

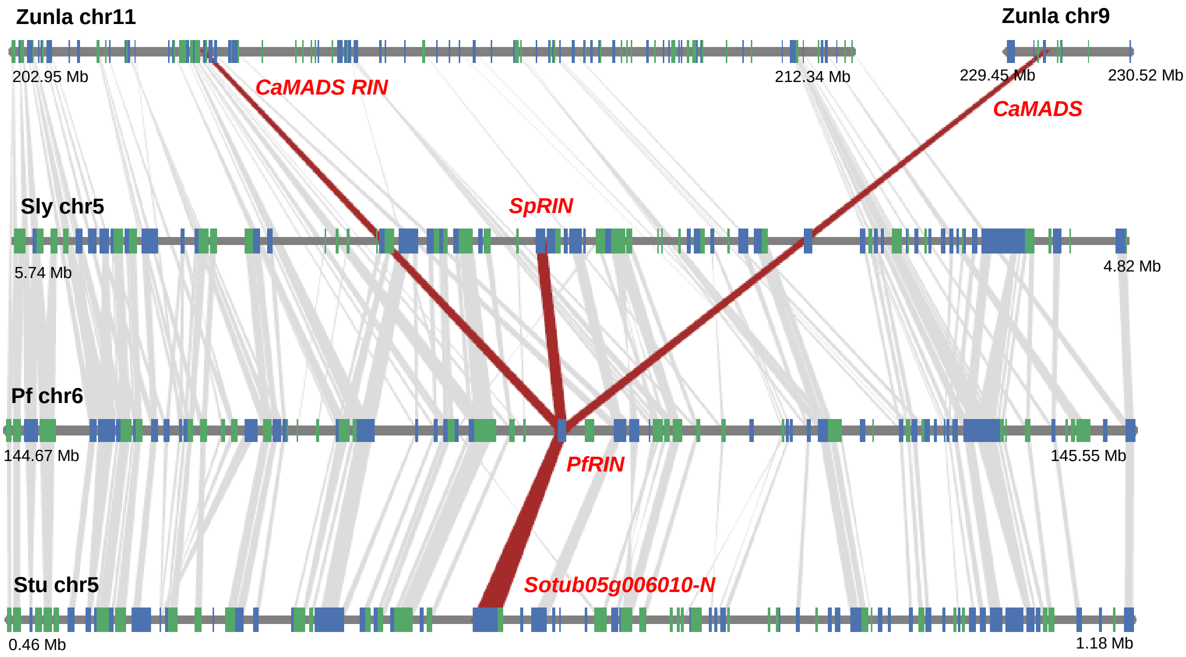

Supplementary Fig. 31 The microsynteny patterns of the RIN subclade among *C. annuum*, *S. lycopersicum, P. floridana,* and *S. tuberosum.* Green and blue rectangles represent predicted genes and their relative locations. Grey wedges connect matching gene pairs; orthologous gene pairs (RIN-like) are highlighted in brown

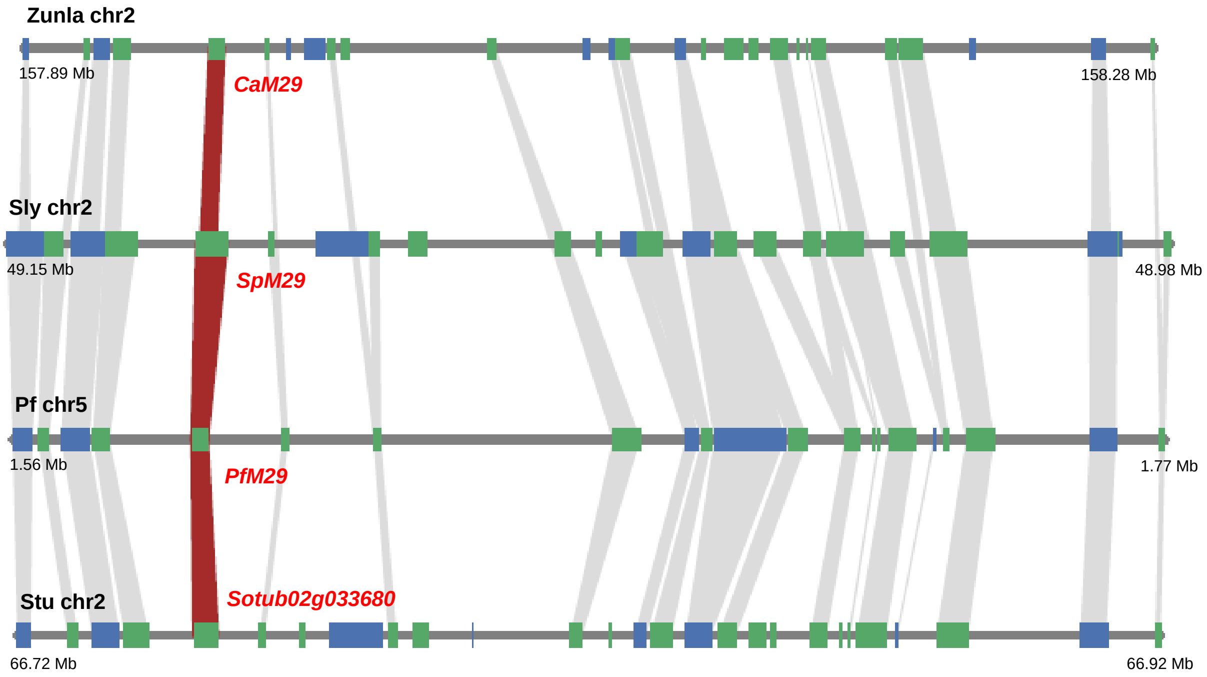

Supplementary Fig. 32 The microsynteny patterns of the M29 subclade among *C. annuum*, *S. lycopersicum, P. floridana,* and *S. tuberosum.* Green and blue rectangles represent predicted genes and their relative locations. Grey wedges connect matching gene pairs; orthologous gene pairs (M29-like) are highlighted in brown

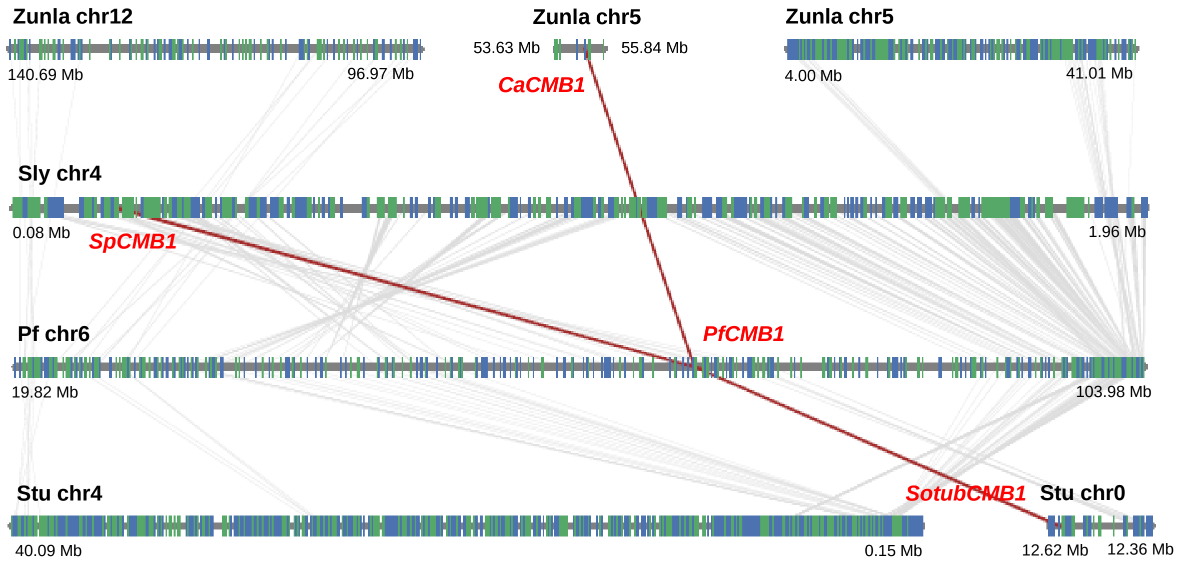

Supplementary Fig. 33 The microsynteny patterns of the CMB1 subclade among *C. annuum*, *S. lycopersicum, P. floridana,* and *S. tuberosum*. Green and blue rectangles represent predicted genes and their relative locations. Grey wedges connect matching gene pairs; orthologous gene pairs (CMB1-like) are highlighted in brown

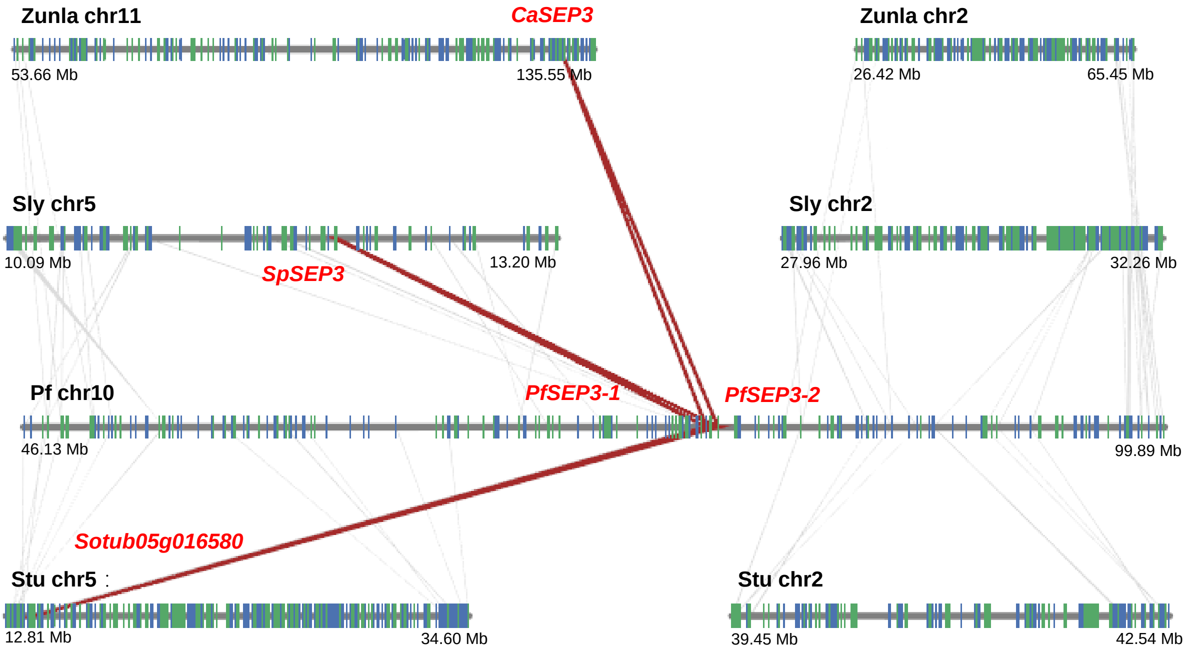

Supplementary Fig. 34 The microsynteny patterns of the SEP3 subclade among *C. annuum*, *S. lycopersicum*, *P. floridana*, and *S. tuberosum*. Green and blue rectangles represent predicted genes and their relative locations. Grey wedges connect matching gene pairs; orthologous gene pairs (SEP3-like) are highlighted in brown

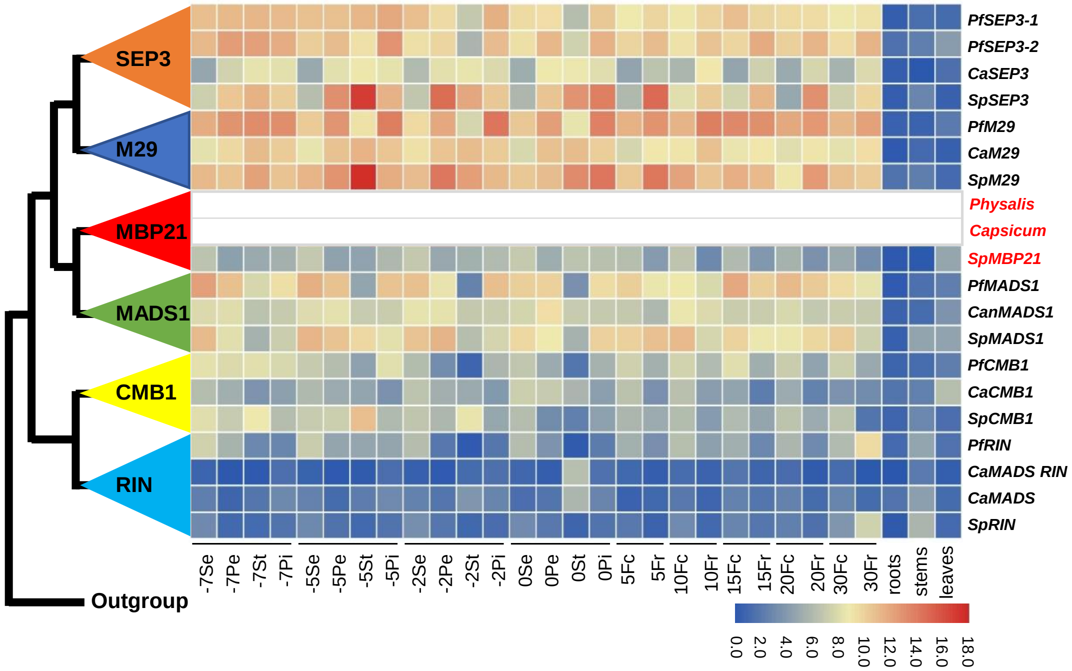

Supplementary Fig. 35 Expression variation of *SEP*-like genes among *Physalis*, *Solanum,* and *Capsicum.* The expression of the *SEP*-like genes in various indicated organs revealed by qRT-PCR and arranged in a phylogenetic framework. Total RNAs were isolated from sepals (Se), petals (Pe), stamens (St), and pistils (Pi) from -7, -5, and -2 days before flowering (day 0); fruiting calyx (Fc) and fruits (Fr) from 5, 10, 15, 20, and 30 days after flowering. The roots, stems, and leaves from 14-day-old seedlings were also included. The expression levels of *PfACTIN*, *SpACTIN,* and *CaUbiquitin* were used as the internal control in *Physalis*, *Solanum,* and *Capsicum*, and each biological sample was repeated three times. The white box indicates the *MBP21* loss in *Physalis* and *Capsicum* highlighted in red

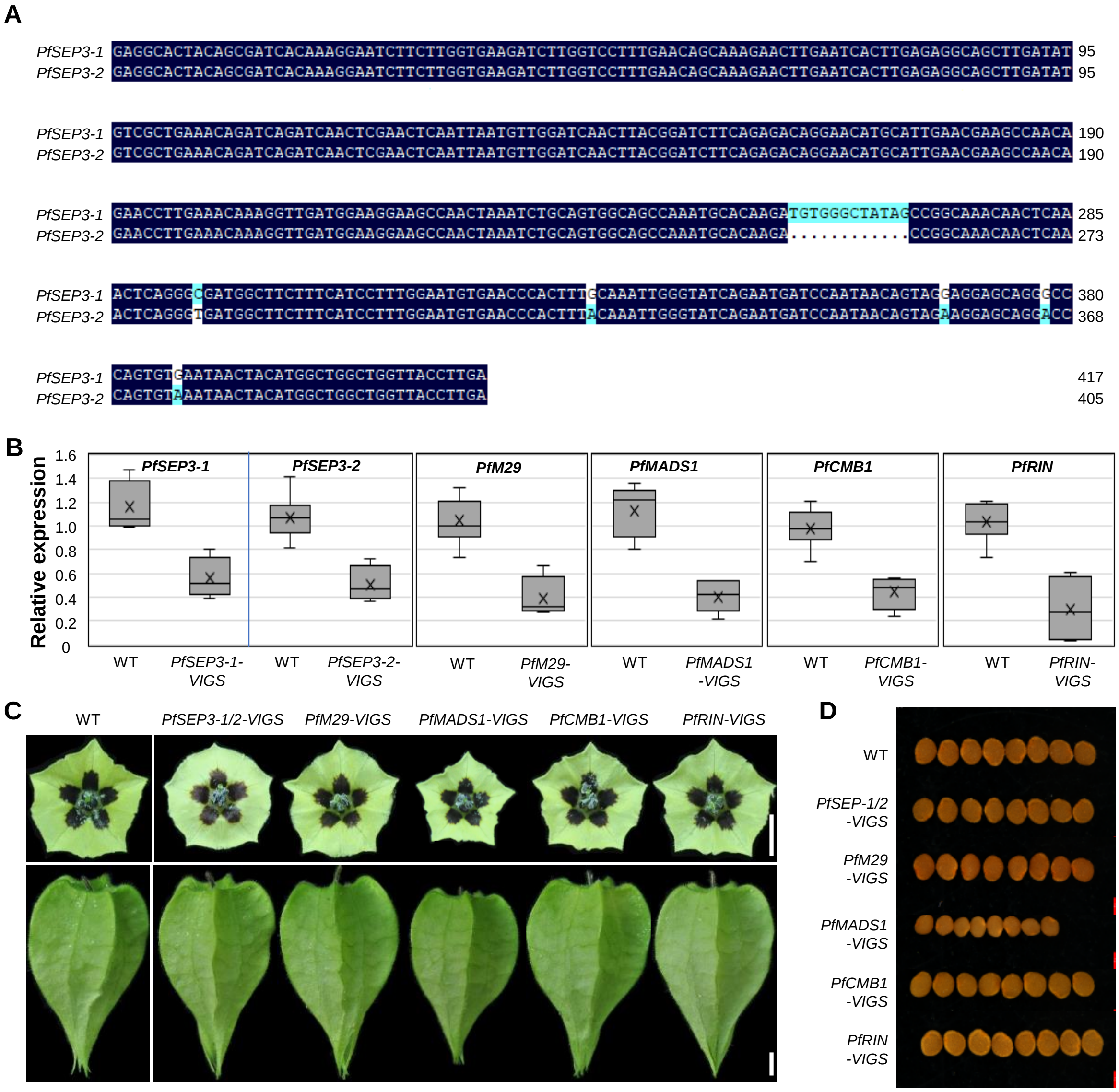

Supplementary Fig. 36 VIGS analysis of *SEP*-like genes in *P. floridana.* **A** The VIGS probe of *PfSEP3-1* and *PfSEP3-2*. Both paralogs had a high identity, and they were downregulated in the fragments of either *PfSEP3-1* or *PfSEP3-2* used for VIGS. **B** Relative gene expression in VIGS floral organs. The results were obtained by qRT-PCR with at least three independent biological samples. **C** Morphology of flowers and fruits. No significant changes in floral organs or fruits indicate that each gene was down-regulated compared with the wild type (WT). **D** Seed morphology in the indicated genetic background. Bar = 0.5 cm

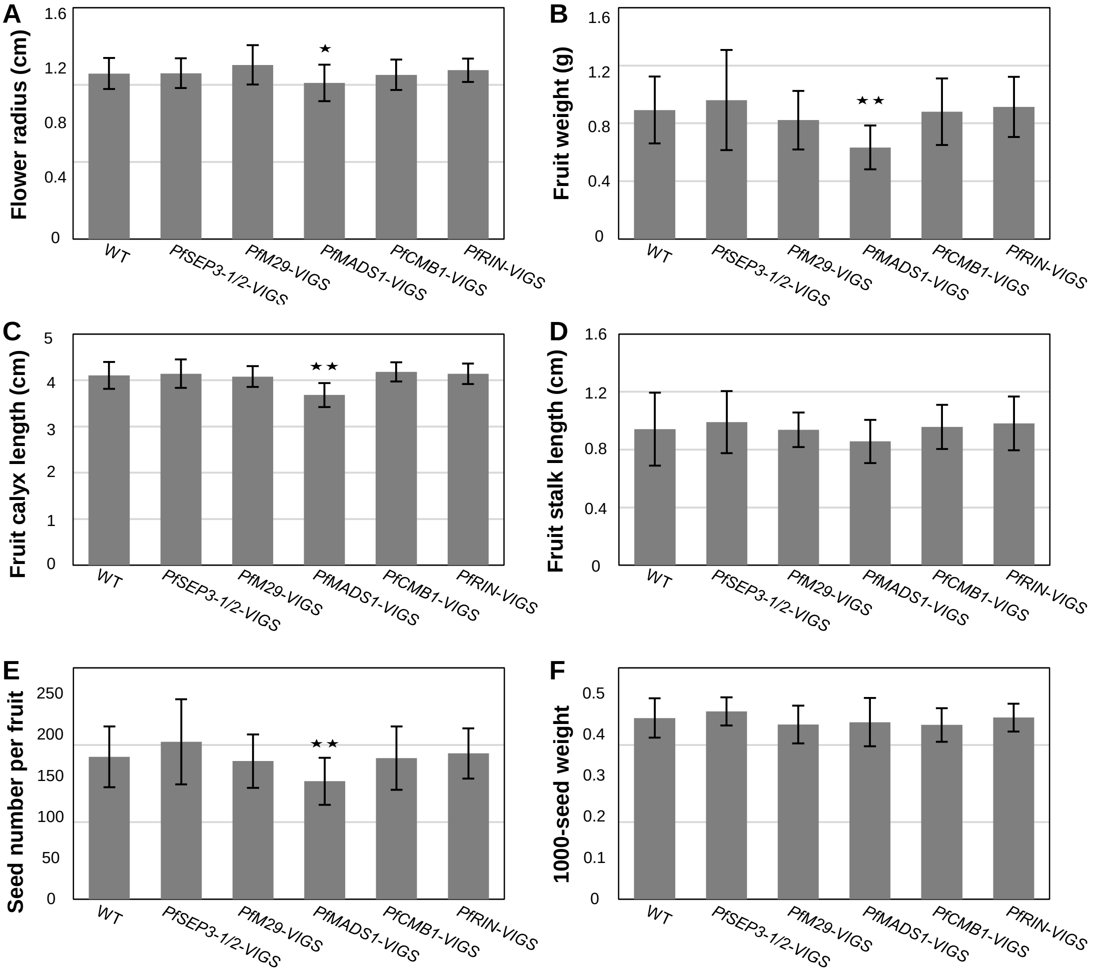

Supplementary Fig. 37 Quantification of floral phenotypes in *SEP*-like VIGS *Physalis*. **A** Flower radius. **B** Fruit weight. **C** Fruit calyx length. **D** Fruit stalk length. **E** Seed number per fruit. **F** 1000-seed weight. Asterisks indicate the significance of each trait in the indicated VIGS samples relative to the wild type (WT) evaluated by the two-tailed Student' s *t* test

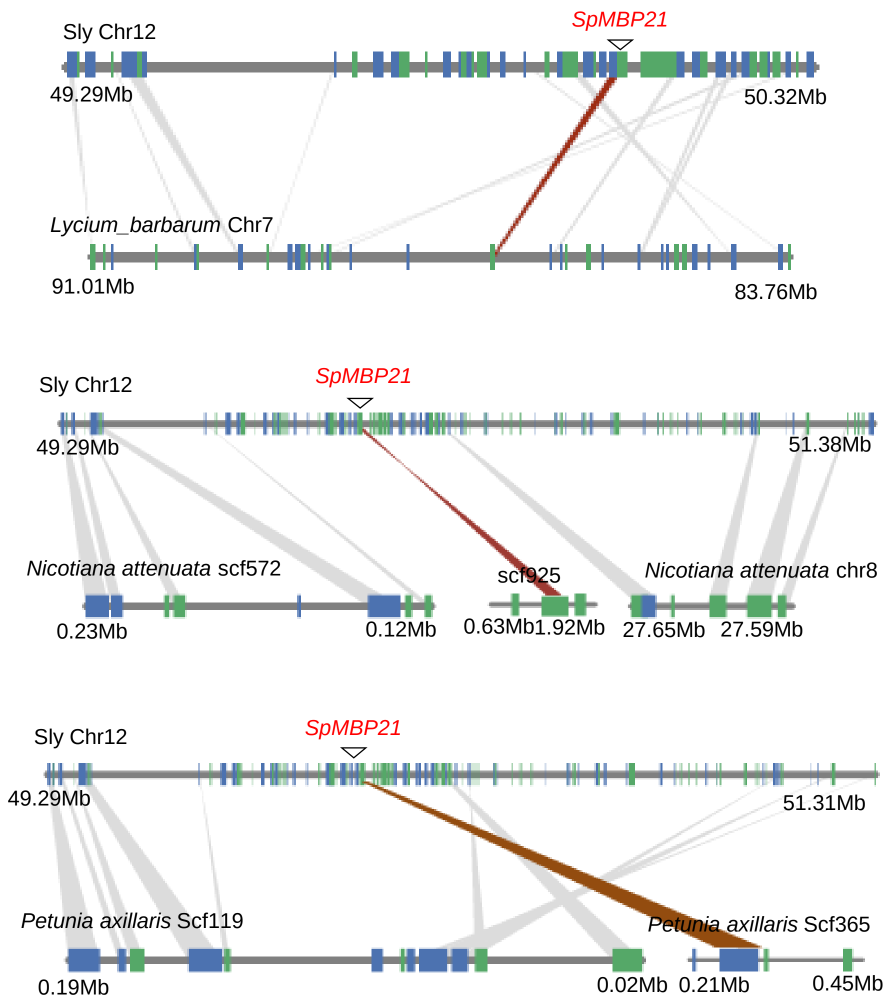

Supplementary Fig. 38 The existence of *MBP21* in other available Solanaeous genomes. The microsynteny of the *MBP21* subclade-containing region among the four indicated species from Solanaceae. The brown linear region represents the syntenic gene blocks of the *MBP21* gene. The *MBP21* gene was detected in the *Lycium*, *Nicotiana*, and *Petunia*, however, only *Lycium MBP21* displayed a relatively good synteny with *Solanum SpMBP21*

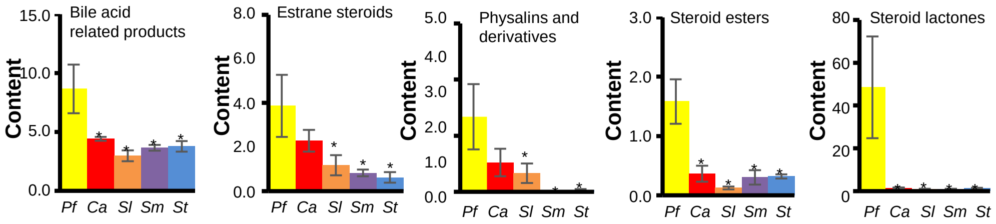

Supplementary Fig. 39 Natural variation of steroid-related derivatives content in various solanaceous fruits. The 25-day-old fruits after fertilization of the indicated solanaceous species were subjected to LC-MS/MS analyses. The full list of the detected steroids and derivatives is presented in Supplementary Table 36, and only the steroid-related species that were higher in relative abundance determined by spectral count in *Physalis* compared to other species are presented as indicated. *, significant difference at $p < 0.05$ according to Tukey's tests in one-way ANOVA relative to *P. floridana* (*Pf*). *Ca*, *C. annuum*; *Sl*, *S. lycopersicum*; *Sm*, *S. melongena*; *St*, *S. tuberosum*.

To explore the biochemical diversity within the Solanaceae, we detected the steroids and steroid derivatives in the fruits of five representative Solanaceae species, *P. floridana* P106, *S. lycopersicum* (Gold coins), *S. melongena* (Zixiu), *S. tuberosum* (Xingjia 2), and *C. annuum* (Rookie) by liquid chromatography-tandem mass spectrometry (LC-MS/MS)-based untargeted metabolomics; 18 steroid-related species were detected as naturally varying among these species (Supplementary Table 36). *Physalis* fruits had a significantly higher concentration of the related products or derivatives of bile acids, estrane steroids, physalins, steroid lactones, and steroid esters as compared to *Solanum* species and the most closely related *Capsicum*

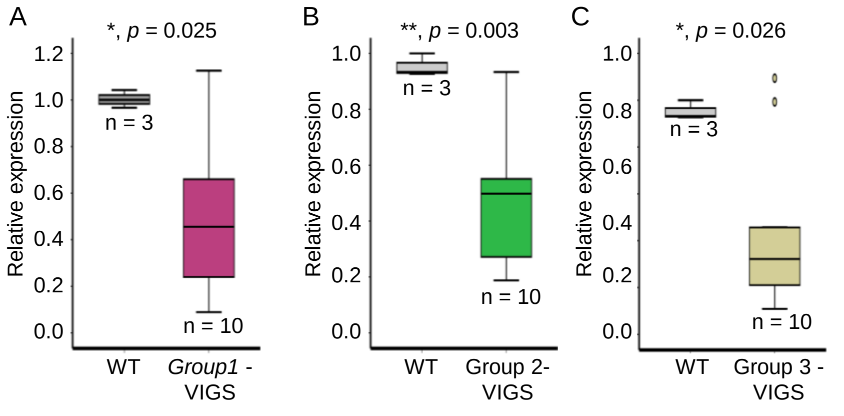

Supplementary Fig. 40 The downregulation of *PfSQE* genes in VIGS analysis using each group-specific probe. Total RNAs of fruits from the wild type (WT) and three groups of VIGS transgenic plants were subjected to qRT-PCR. The fruit number (n) is indicated. Significance relative to WT was evaluated by a two-tailed Student' s *t* test; *, $0.01 < p < 0.05$; **, $p < 0.01$

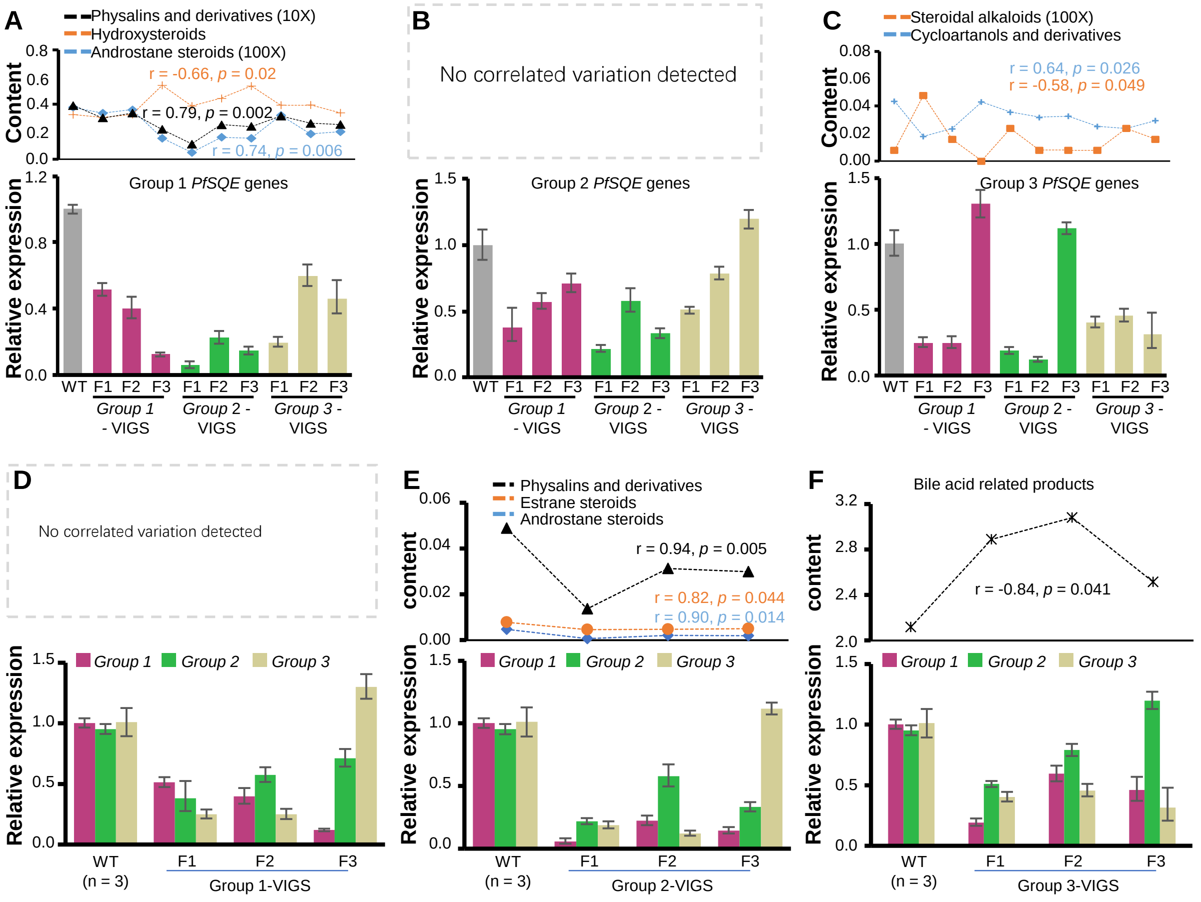

Supplementary Fig. 41 Correlations between the expression of *PfSQE* genes and steroid-related derivatives content in *PfSQE*-VIGS fruits. A-C Correlations between the expression of *PfSQE* genes and steroid-related derivatives content in the three fruits (F1–F3) of each group in the *PfSQE*-VIGS analysis. **A** Group 1 gene expression and correlated steroid derivatives content. **B** No correlation was found between group 2 gene expression and steroid-related derivatives content. **C** Group 3 gene expression and correlated steroid-related derivatives content. The columns represent the expression levels of the *PfSQE* genes in the *PfSQE*-VIGS fruits of *P. floridana,* while the colored dashed lines indicate the steroid-related derivatives correlated with the indicated *PfSQE* gene expression. **D-F** Correlations between the *PfSQE* family gene expression and steroids or steroid derivatives content in VIGS-fruits. **D** No correlations were found between *PfSQE* family gene expression and steroids or steroid derivatives content in three Group 1 *PfSQE*-VIGS fruits (F1–F3). **E** Correlations between *PfSQE* family gene expression and steroids or steroid derivatives content in three Group 2 *PfSQE*-VIGS fruits (F1–F3). **F** Correlations between *PfSQE* family gene expression and steroids or steroid derivatives content in three Group 3 *PfSQE*-VIGS fruits (F1–F3). *PfSQE* family gene expression was calculated by simply adding each expression level of the three group genes. The columns represent the expression levels of the *PfSQE* genes in the *PfSQE*-VIGS fruits of *P. floridana,* while the colored dashed lines indicate the steroid-related derivatives correlated with the indicated *PfSQE* gene expression. Relative expression level in WT was set as 1.0. The fruits after 14 days VIGS treatments were subjected to both qRT-PCR and LC-MS/MS analyses.

Based on the sequence similarity, two groups (Group 1 and Group 2) of the *Physalis*-specific *SQE* genes and a Solanaceae conserved group were subjected to VIGS in *P. floridana* (Supplementary Fig. 23). Our VIGS analyses were aimed at the group-specific downregulation of *PfSQE* genes; the three groups of genes were found to be simultaneously downregulated (Supplementary Fig. 40), suggesting homologous interference. The metabolomics results revealed that the contents of androstane steroids, cycloartanols and derivatives, and physalins and derivatives were significantly reduced and were correlated with the *PfSQE* downregulation in *P. floridana*, particularly with Group 1, the *Physalis*-specifically expanded *SQE* genes. Nonetheless, considering gene downregulation of a single group or all three groups, different correlation patterns were observed (Supplementary Table 37), indicating the complexity of the *PfSQE* gene in affecting the steroid derivative variation in *Physalis*. In accordance with chemical variation among solanaceous species (Supplementary Fig. 39; Supplementary Table 36), in these VIGS analyses, only the level of physalins and derivatives was consistently reduced and correlated with the expression of the *PfSQE* genes, suggesting that the *PfSQE* family may primarily determine the level of physalins and derivatives, characteristic steroid-related species in Physaleae
